# Effects of Antidepressants on Sleep in Post-traumatic Stress Disorder: An Overview of Reviews

**DOI:** 10.2174/1570159X21666230801144328

**Published:** 2023-08-15

**Authors:** Andreas S. Lappas, Zoi A. Polyzopoulou, Nikos Christodoulou, Vasilios-Panteleimon Bozikas, Myrto T. Samara

**Affiliations:** 1 Department of Psychiatry, Medical School, General University Hospital of Larissa, University of Thessaly, Larissa, Greece;; 2 Department of Geriatric Liaison Psychiatry, Royal Gwent Hospital, Newport, United Kingdom;; 3 Department of Psychology, University of Western Macedonia, Florina, 53100, Greece;; 4 School of Medicine, University of Nottingham, Nottingham, England, United Kingdom;; 5 II Department of Psychiatry, School of Medicine, Aristotle University of Thessaloniki, Lagkada Str. 196, 56430Thessaloniki, Greece

**Keywords:** Post-traumatic stress disorder, PTSD, sleep, insomnia, somnolence, nightmares, dreams, meta-analysis

## Abstract

Antidepressants are a commonly used, easily accessible, and overall safe treatment option for post-traumatic stress disorder (PTSD). The present review aims to evaluate the efficacy and safety of antidepressants in treating sleep disturbances in patients with PTSD. PubMed and the Cochrane Library were searched (July 2022) for systematic reviews and meta-analyses on the treatment of PTSD. Moreover, PubMed and ClinicalTrials.gov were searched for individual trials investigating the antidepressant treatment of PTSD (up to September 2022), and reference lists of all possibly relevant identified studies were screened. Sleep-related outcomes, *i.e*., total sleep time, sleep quality, dreams/ nightmares, insomnia, and somnolence, were extracted independently by at least two reviewers. Meta-analytic evaluations were performed wherever possible. 39 randomised controlled trials (RCTs) were identified; data from pooled analyses, reviews, and observational studies were used for antidepressants with a weak evidence base or when their findings were deemed important. Overall, scarce data exist on the effects of antidepressants on sleep outcomes among patients with PTSD. Some evidence may support the use of amitriptyline, nefazodone, paroxetine, and sertraline for improving sleep in patients with PTSD. Τhere was a meta-analytical trend indicating improvement of nightmares with fluoxetine, less insomnia with amitriptyline and more with brofaromine, as well as more somnolence with paroxetine *vs.* placebo, respectively. However, data from more than 1 RCT with a considerable number of patients were only available for paroxetine. Evidence is insufficient to draw safe conclusions. More and better-designed RCTs, with consistent reporting of sleep-related outcomes, are needed.

## 1 INTRODUCTION

Post‐traumatic stress disorder (PTSD) is an established psychiatric disorder that results following a traumatic event or series of events. It is a relatively common disorder, with a year prevalence of 4.7% and a lifetime prevalence of 6.1% in the US [1]; however, rates of PTSD tend to vary according to the type of trauma and the population experiencing it. For example, man-made disasters and violence are more potent traumatic experiences than natural disasters or accidents [2], and PTSD is more common in post-conflict settings [3]. PTSD rates are reported considerably more frequently in women (10.4% *vs.* 5%) [4, 5]. Other predisposing factors include a past history of depression, anxiety or trauma, neuroticism, poor social support, younger age, previous marriage, poor education, and rural residency, among others [1, 6].

PTSD features hyperarousal symptoms, such as hypervigilance, symptoms of re-living the traumatic experience, such as flashbacks and intrusive thoughts of the traumatic event, and avoidance of situations that are perceived as reminiscent of the event. It is commonly accompanied by anxiety and depression, and is associated with significant disability, dysfunctionality, and high economic costs [7-9].

Notably, sleep disturbances are common and troublesome in PTSD, with patients most frequently experiencing insomnia and nightmares [10]. Sleep is an important symptom dimension in PTSD, as it may independently affect clinical progress and quality of life [11], while it has also been implicated in the causality of the disorder [12]. Corroborating evidence suggests that even other disorders featuring disturbed sleep as one of their central symptoms are linked to PTSD [13].

Clinicians very frequently resort to prescribing antidepressants in PTSD, as they offer meaningful symptomatic relief for a range of PTSD symptoms. The antidepressants’ actions and adverse effects have classically been attributed to their affinity for pre- and post-synaptic neurotransmitter receptors for serotonin, noradrenaline, dopamine, and other major neurotransmitters. The current understanding of the neurobiology of PTSD focuses on monoamine neurotransmitters and the hypothalamic-pituitary-adrenal axis, both of which are affected by antidepressants directly or indirectly [14-17].

With regards to the treatment of sleep disturbances, although benzodiazepines and Z-drugs have proven efficacy for the treatment of insomnia [18], the well-recognized concerns regarding misuse and dependency potential often lead clinicians into off-label use of other agents, such as antidepressants [19]. This is particularly important for patients with PTSD who often have high rates of substance misuse, perhaps as an attempt for self-medication [20, 21].

Nevertheless, and despite the widespread use of antidepressants in PTSD, it is still not clear how effective they are in treating sleep-related disturbances or, indeed, whether they may exacerbate them. On the other hand, recent evidence postulates that targeting sleep itself can be therapeutic for PTSD [11]. Prescribing antidepressants for PTSD, therefore, presents clinicians with a conundrum, since some antidepressants themselves might have sleep-related adverse effects. This overview of reviews attempts to clarify this question by comprehensively reviewing and appraising available evidence on the topic from a clinical perspective. We aimed to offer an evidence-based compilation of the antidepressant effects on sleep among patients with PTSD. The review could also serve as a research utility, as it effectively highlights gaps in our evidence base and prompts future research.

## 2 METHODOLOGY

### 2.1 Search Strategy and Identification of Trials

We (AL, ZP, AL, NC) searched PubMed and the Cochrane Library (last search July 2022) for systematic reviews and meta-analyses on the treatment of post-traumatic stress disorder (PTSD) and screened all included trials. Moreover, we searched for individual trials in PubMed and ClinicalTrials.gov (with the last search conducted in September 2022). Reference lists of all possibly included trials were also screened. All studies examining any antidepressant pharmacotherapy, either as monotherapy or as an add-on to other types of pharmacotherapy, in patients with an established diagnosis of PTSD were considered for inclusion, irrespective of how the diagnosis was made, study design, sample size, and publication language. We aimed to identify all trials reporting on a sleep-related outcome, irrespective of the objectives of the trial, as long as the comparisons were between an antidepressant *vs.* placebo or an antidepressant *vs.* another antidepressant. Thus, all antidepressant trials in PTSD were screened, and full texts were assessed by at least two reviewers. Disagreements were resolved by discussion with a third author (MS). We decided not to present case reports and case series with less than 10 patients if ≥ 2 Randomised Controlled Trials (RCTs) reporting on sleep-related outcomes were available, and we applied this to all antidepressants included in this study.

### 2.2 Outcomes Extracted

At least two authors (AL, ZP, MS) independently extracted all data. Study design and duration, sample size, antidepressant dose, and concomitant use of other treatments were recorded. Any relevant sleep-related efficacy outcomes were extracted, namely total sleep time, sleep quality, and dreams/nightmares, as well as sleep-related tolerability and safety outcomes, namely insomnia, somnolence, increased dream activity/nightmares, and any other parasomnias reported as side effects.

### 2.3 Statistical Analysis

Meta-analytic calculations were performed whenever possible. For dichotomous outcomes, the primary effect size measure was the relative risk (RR) with corresponding 95% confidence interval, since clinicians understand it more intuitively than odds ratios. For continuous outcomes, we preferred absolute numbers, for example, total sleep time in minutes, and presented them as mean differences (MDs), but if different scales were used, *e.g*., for the assessment of sleep quality, the effect sizes were calculated as Hedge’s g standardized mean differences (SMDs). Unreported SD values were calculated from other statistics or from the average of the other studies. As we expected considerable heterogeneity between studies, we applied the DerSimonian and Laird random-effects model throughout [22]. The degree of heterogeneity was estimated by the I^2^ statistic [23] and a chi-square test of homogeneity (α set at *p <* 0.1). Meta-analytic calculations were done with RevMan.

## 3 RESULTS

Details of 39 included RCTs are presented in Tables ****1**-**24****. 2 of these were discontinuation studies [24, 25]. The trials were published from 1988 to 2021, whereas 2 remain unpublished to date [26, 27]. RCTs had a median sample size of 75 participants per study (range 15-531). Most trials compared antidepressants only with placebo. Two studies compared sertraline with placebo and another antidepressant, namely citalopram and venlafaxine [28, 29]. Few studies did not have placebo control, *i.e*., one study compared fluoxetine with moclobemide and tianeptine [30], one fluvoxamine with reboxetine [31], one mirtazapine with sertraline [32], one nefazodone with sertraline [33], and one paroxetine with mirtazapine [34]. Most trials examined antidepressants as monotherapy apart from 7 RCTs that allowed the continuation of previously prescribed psychotropics [35, 36], or combined the antidepressant under investigation with another psychotropic drug [26, 37] or psychotherapy [38-41]. Pooled analyses, reviews, and observational studies were included in addition to RCTs whenever the evidence base was weak, or the findings related to our study outcomes were important.

Studies awaiting assessment are presented in supplement 1.

Below, we first present in detail the extracted information from all included studies. We have categorised antidepressants based on the main classes they belong to. We describe the study design, population, and findings relevant to our study outcomes for each antidepressant in the form of a narrative text, followed by a table for each drug we discuss.

We then present the results of our meta-analytic evaluation (Figs. **1**-**8**).

## 4 SELECTIVE SEROTONIN REUPTAKE INHIBITORS (SSRIS)

### 4.1 Citalopram

Citalopram is a widely used SSRI with proven efficacy and favourable tolerability for the treatment of depression [42, 43].

There is limited evidence with regards to the efficacy of citalopram for the treatment of PTSD symptoms [21, 44, 45], and there is even less evidence with regards to outcomes relevant to our study.

To our knowledge, there is no evidence from systematic reviews/meta-analyses examining the effects of citalopram on sleep disturbances among PTSD patients; there is only 1 RCT with relevant information, which we have discussed elsewhere [28] (that of sertraline), but will include here as well for the sake of completeness. There is also a limited number of observational studies and case reports. Overall, the quality of evidence is poor, and information regarding our study outcomes is very limited.

#### 4.1.1 Evidence from Randomised Controlled Trials (RCTs)

There is no RCT evidence regarding any potential therapeutic effects of citalopram on sleep disturbances in patients with PTSD. The only RCT reporting on outcomes relevant to our study is a 10-week double-blind, randomised, parallel-group, placebo-controlled clinical trial [28], which examined the effects of sertraline monotherapy (N = 32, mean dose = 134.1 mg, range = 50-200 mg daily), citalopram monotherapy (N = 25, mean dose = 36.2 mg, range = 20-50 mg daily), and placebo (N = 10) on PTSD symptoms and autonomic reactivity to trauma cues among 58 patients (18-64 years old) with a mixture of civilian and combat-related PTSD. As required, treatment with diphenhydramine for sleep problems was allowed throughout the trial. Only data for insomnia as a treatment-emergent side effect are reported: 43.5% for the sertraline group *vs.* 60% for the citalopram group *vs.* 70% for the placebo group, but no formal statistical analysis has been performed.

#### 4.1.2 Observational Studies and Case Reports

In an 8-week, open-label, flexible-dose clinical trial, English *et al.* [46] examined the effects of citalopram monotherapy (mean dose 34.44 mg, range 20-40 mg daily) on PTSD symptoms among 18 male veterans (mean age = 55, range = 49-74 years, 17 completed at least 4 weeks) with chronic combat PTSD. No information regarding the concomitant use of other psychotropic medication during the trial has been provided. Although the therapeutic effects of citalopram on sleep-related PTSD symptoms were not examined as study outcomes, data on tolerance and sleep-related treatment-emergent adverse effects are reported. The authors have reported both insomnia and somnolence/sedation as common side effects of citalopram in this cohort, but the results are not quantified. They also report that 1 patient dropped out at week 4 due to experiencing sedation, which was intolerable.

In another 8-week, fixed-dose, open-label trial, Seedat *et al.* [47] examined the effects of citalopram monotherapy (20-40 mg daily) on PTSD symptoms among 14 civilians and veterans (7 males, 2 veterans, and 11 completers included in the data analysis) with PTSD (minimum duration of symptoms = 3 months, 5 had comorbid major depression and 1 comorbid dysthymia). Concomitant use of other psychotropic medications was not allowed during the trial. Again, the therapeutic effects of citalopram on sleep-related PTSD symptoms were not examined as study outcomes. The authors report that 5 patients (45.5%) developed daytime sedation as a treatment-emergent adverse effect, but no further information relevant to our outcomes is provided.

Khouzam *et al.* [48] have reported their experience in treating 2 male Persian Gulf veterans with chronic combat PTSD (22 and 36 years old) with citalopram (20-40 mg daily). No information on comorbid psychiatric diagnoses and concurrent treatment with other psychotropics is provided. They report that the 22-year-old veteran was treated with citalopram 20 mg daily for 3 months, and although he initially experienced daytime sedation as a treatment-emergent adverse effect, the medication was switched to night-time and resulted in a marked improvement in his sleep and disappearance of trauma-related nightmares.

Table **1** below summarises the findings discussed above.

### 4.2 Escitalopram

The (S) enantiomer of citalopram has been developed and marketed as escitalopram. The racemic citalopram has a relatively inconsistent therapeutic action at the lowest doses, often requiring dose increase to exert significant antidepressant and anxiolytic action [49]. The (R) enantiomer, however, has weak antihistamine properties, and has traditionally been thought to be responsible for the dose-dependent QTc prolongation and consequent risk of arrhythmia. The benefit of isolating the S-enantiomer, therefore, seems significant from a pharmacological point of view, potentially offering a good response at lower doses through pure serotonin transporter (SERT) inhibition and without the risk of dose-dependent adverse effects [50]. Recent evidence, however, challenges this line of thought and indicates an association between S-citalopram and QTc prolongation [51, 52]. Escitalopram is considered perhaps the best-tolerated SSRI, with the fewest CYP450-mediated interactions [53].

There is no significant evidence base to support the use of escitalopram for the treatment of PTSD symptoms, as few studies have examined its efficacy and safety [21, 44, 45].

Even fewer studies report on outcomes relevant to our study, and we present these below.

In a prospective, open-label, uncontrolled 12-week trial, Robert *et al.* [54] examined the effectiveness of escitalopram (10-20 mg daily) for the treatment of PTSD symptoms among 25 male veterans (mean age = 55.6 ± 7.68 years) with severe, chronic and treatment-resistant combat PTSD (comorbid with major depression among 19 of these patients). Minimal intermittent use of hypnotics was allowed for the first 2 weeks of the trial only. Sleep quality was examined using the PSQI (Pittsburgh Sleep Quality Index) and the PSQI-A (Pittsburgh Sleep Quality Index-Addendum for PTSD) among the 24 completers. There were no significant differences found between the PSQI scores at baseline *vs.* endpoint (baseline = 12.67 ± 2.65 *vs.* endpoint (12 weeks) = 11.20 ± 3.50, *p* = 0.0616). Similarly, no significant improvement in PSQI-A scores was identified (baseline = 7.59 ± 4.15 *vs.* endpoint (12 weeks) = 7.58 ± 4.92, *p* = 0.4315). The authors have also reported on sleep-related treatment-emergent adverse effects. 3 patients (12%) experienced insomnia and 6 (24%) experienced sedation as a treatment-emergent side effect. 1 patient (4%) experienced “odd dreams,” but it is unclear if these were nightmares resembling the trauma or not.

In another prospective, open-label, uncontrolled 12-week trial, Ramaswamy *et al.* [55] examined the effects of escitalopram treatment (10-20 mg daily) on autonomic function among 11 male Iraqi veterans with combat PTSD and comorbid depression (mean age = 28, range = 19-55). Concurrent intermittent rescue sleep therapy with zolpidem was allowed during the trial. Therapeutic effects on sleep parameters relevant to our study were not examined; however, data on treatment-emergent adverse effects are available. Specifically, insomnia was identified as a treatment-emergent adverse effect among 3 patients (27.3%). One patient also experienced somnambulism, although this was thought not to be related to treatment with escitalopram.

Qi *et al.* [56] conducted an open-label trial to investigate the effectiveness and safety of escitalopram (10-40 mg daily) in the short term (3 months) and in the medium term (6 months) in treating civilians with chronic PTSD due to a variety of types of index trauma (36 completers, 18 males, mean age = 43.5 ± 13.4 years). There is no information regarding concomitant as required treatment with other medication for insomnia. Again, therapeutic effects on sleep are not studied as primary or secondary outcomes, but relevant data on treatment-emergent side effects are available. For the first 3 months, 4 patients (11.1%) experienced drowsiness as a treatment-emergent side effect, but only 1 (2.8%) continued experiencing this adverse effect at 6 months of treatment. 1 patient (2.8%) experienced insomnia during the first phase of 3 months, which did not continue during the second phase, during which no patient experienced insomnia (0%).

Table **2** below summarises the findings discussed above.

### 4.3 Fluoxetine

With little effects on norepinephrine and dopamine reuptake at therapeutic levels, fluoxetine inhibits the reuptake of serotonin (5-HT), resulting in higher concentrations of the neurotransmitter in the synaptic cleft and, eventually, greater postsynaptic neuronal activity [57].

There are several controlled and uncontrolled trials with different types of study designs, case series and case reports that examine the effectiveness of fluoxetine in the treatment of PTSD symptoms [24, 25, 30, 58-71]. There is high-quality evidence suggesting that fluoxetine is a safe and efficacious treatment for PTSD symptoms [21].

To the best of our knowledge, 6 RCTs report on sleep-related effects of fluoxetine, and these are discussed below and summarised in Table **3**.

#### 4.3.1 Randomised Controlled Trials

Based on the data from a randomised, double-blind, placebo-controlled 12-week trial examining the efficacy of fluoxetine for the treatment of PTSD conducted by Connor *et al.* [58], Meltzer-Brody *et al.* [72] analysed pre- and post-treatment scores for PTSD symptom clusters and individual items. The study population was 54 civilians with PTSD (32-44 years of age). 27 were randomised on fluoxetine 10-60 mg daily and 27 on placebo. 53 were included in the analysis (27 from the fluoxetine group and 26 from the placebo group) and two-thirds of this sample were 12-week completers. Items “trouble sleeping” and “dreams/nightmares” were examined as items of the Davidson Trauma Scale (DTS), and the Structured Interview for PTSD (SIP). On the DTS, “trouble sleeping”, but not “dreams/nightmares”, showed a significant improvement in the fluoxetine group *vs.* placebo (from 4.96 to 2.62 in the fluoxetine group *vs.* 6.25 to 4.38 in the placebo group, *p* = 0.0229). However, on the SIP, there was no significant difference between the effects of fluoxetine and placebo for both sleep-related outcomes. Based on the above, a significant improvement in self-rated insomnia symptoms was noted (DTS), but this was not captured on the clinician-rated instrument (SIP).

Barnett *et al.* [62] attempted to delineate treatment-emergent symptoms (TES) potentially associated with fluoxetine treatment of PTSD using data from 2 similarly designed randomised, double-blind, placebo-controlled 12-week trials [58, 60]. The Connor *et al.* trial [58] has been discussed above. The Hertzberg *et al.* trial [60] was of identical design to the Connor *et al.* trial, but the patient population was 12 male veterans with severe, chronic combat PTSD. Fluoxetine was prescribed at a dosage range of 10-60 mg/day in both trials. The patient-rated Severity of Symptoms Scale (SOSS) was used, and “poor sleep” was one of the items examined. The results suggested that patients from both trials tolerated fluoxetine well without pronounced activating side effects. For sleep in particular, only participants with comorbid panic disorder showed an increased tendency to experience “poor sleep” as a treatment-emergent symptom, but this was not found to be statistically significant. The authors have only reported TES incidence values for the SOSS items, which have reached statistical significance.

Davidson *et al.* [25] conducted a trial with an initial 6-month open-label phase, followed by double-blind randomised maintenance treatment with fluoxetine or placebo substitution for another 6 months. Of 123 patients initially enrolled, 62 were randomised to receive either fluoxetine (10-60 mg daily, mean = 48.6 mg) or placebo, and data from 57 of those were used for the analysis (30 in the placebo arm, and 27 in the fluoxetine arm). Fluoxetine showed a significant benefit over placebo in relapse prevention (NNT = 3.6). Although insomnia or sleep quality were not reported as study outcomes, it is reported that insomnia and nightmares were the most common adverse effects in both the fluoxetine and the placebo arms: 5/27 in the fluoxetine arm *vs.* 5/30 in the placebo arm for insomnia, and 5/27 in the fluoxetine *vs.* 6/30 in the placebo arm for nightmares.

In a multi-centered double-blind, placebo-controlled trial by Martenyi *et al.* [63], 301 patients with both combat and non-combat-related PTSD (aged 18-65 years, 81% male) were randomised to either acute 12-week fluoxetine treatment (N = 226, mean exposure 80 days, mean endpoint dose 57 mg daily) or 12-week placebo treatment (N = 75). Fluoxetine was significantly more efficacious than placebo in all outcome measures evaluating the severity of PTSD symptoms, and there were no significant safety differences. Insomnia was reported in this study as a common adverse effect in both the fluoxetine and the placebo arms: 12% (number of patients not provided) *vs.* 12% (number of patients not provided), respectively, and there were no statistically significant differences for any single adverse effect.

131 responders of the initial Martenyi *et al.*’s study [63] agreed to continue on the second 24-week relapse prevention double-blind, placebo-controlled trial [24]. 69 were randomised to receive fluoxetine (20-80 mg daily, mean dose = 53 mg daily) and 62 placebo. The fluoxetine-fluoxetine group was significantly less likely to experience relapse compared to the fluoxetine-placebo group (χ^2^ = 4.88, *P* = 0.027), and there were no statistically significant differences in the treatment emergent adverse effects between the 2 groups. Insomnia was again reported as the commonest adverse effect in the fluoxetine-fluoxetine group (15%, number of patients not provided) as well as the fluoxetine-placebo group (10%, number of patients not provided), and there were no statistically significant differences in the number of patients reporting any single adverse event between the groups.

Somnolence has also been reported as a side effect, albeit much less consistently compared to insomnia. In a large multicentre, double-blind, 12-week, placebo-controlled trial of 411 randomised patients, Martenyi *et al.* [73] reported failed efficacy of fluoxetine 20 mg and 40 mg daily compared to placebo for the treatment of PTSD symptoms among patients (aged 18-75, 71.5% women) with both combat and non-combat related PTSD. Interestingly, insomnia is not mentioned as a side effect of the medication in this large cohort of patients, but instead, somnolence has been identified as a rather common side effect: 9.2% (N = 163) for the fluoxetine 20 mg daily group *vs.* 11.9% (N = 160) for the fluoxetine 40 mg daily group *vs.* 5.7% (N = 88) for the placebo group. The differences were not statistically significant (*p* = 0.3). There are no further details with regards to the effect of somnolence on sleep quality and other sleep-related outcomes. Although the authors have not commented on this and no predictors for the development of somnolence as a side effect have been reported, a significant difference between this study cohort and all the others reported in this review is the male/female ratio, predominately females across the study groups in the Martenyi *et al.*’s trial [73] as opposed to most other trials discussed, which recruited predominately male participants. Whether this might be one of the reasons why somnolence and not insomnia was identified as a side effect in this cohort is not clear, but it would be interesting to investigate this further.

In an open-label, flexible-dose randomised comparison of fluoxetine, moclobemide, and tianeptine for the treatment of PTSD, Önder *et al.* [30] enrolled 103 patients with post-major earthquake PTSD in a 12-week trial. 38 patients were assigned to the fluoxetine group (mean dose = 27.4 ± 9.8 mg), 35 patients to moclobemide (565.7 ± 115.5 mg), and 30 to tianeptine (41.3 ± 5.8 mg) groups. No difference in terms of efficacy was identified between the groups, and all 3 drugs were found to be efficacious. Sleep quality or insomnia were not reported as study outcomes, but authors reported data on insomnia and sedation as side effects of treatment. Specifically, insomnia was reported as a side effect by 1 patient (2.6%) in the fluoxetine group *vs.* 1 (2.9%) in the moclobemide group *vs.* 0 (0%) in the tianeptine group, and sedation by no patient (0%) in the fluoxetine and (0%) moclobemide groups *vs.* 1 patient (3.3%) in the tianeptine group.

#### 4.3.2 Observational Studies

Nagy *et al.* [69] conducted a prospective, open-label, uncontrolled 10-week trial and enrolled 27 male patients with combat PTSD who received fluoxetine 20-80 mg daily. 19 patients completed at least 3 weeks and were included in the data analysis. Of those, 11 patients (58%) had comorbid panic disorder, 16 (84%) had a major depressive episode at the time of the study, and 5 patients (26%) were prescribed other treatments at the time of the study but their doses were not changed (benzodiazepines, methadone, imipramine, perphenazine, diphenhydramine). 10 patients were 10-week completers. In this trial, insomnia and distressing dreams were examined as items of the CAPS-2 (DSM-III-R) score. At the endpoint, the sub-scores for insomnia dropped significantly from an average of 6.5 (SD = 1.3) to 5.6 (SD = 1.7, F = 3.33, *p <* 0.001), but this was not the case for distressing dreams. Moreover, total sleep measures on Hamilton Depression Scale decreased significantly, with the greatest improvement reported in difficulty falling asleep. A dose-dependent effect in terms of symptomatic improvement was observed, which was felt to be more important than the duration of treatment, with doses 60-80 mg resulting in a robust overall response.

In another uncontrolled case series with a population from a clinical practice setting, Shay [67] treated 28 male Vietnam veterans with treatment-refractory combat PTSD and comorbid depression with fluoxetine 20-80 mg daily. Apart from 4 patients who discontinued the medication, all were followed up for 12-27 months. Symptomatic improvement was observed for 22 out of 26 patients who were treated for more than a month. Shay also reported insomnia as the commonest side effect of fluoxetine treatment, which was observed in 16 out of the 28 patients (57.1%). This was treated with either trazodone or doxepin, which demonstrated good effect in all but one patient, and insomnia was not reported as a reason for discontinuation of fluoxetine in any of the cases.

A summary of the findings discussed above is presented in Table **3** below.

### 4.4 Fluvoxamine

Fluvoxamine is a specific and potent serotonin reuptake inhibitor (SSRI) with antidepressant and anxiolytic properties [74]. Only a few studies have reported on its effectiveness in the treatment of PTSD symptoms and included insomnia and sleep quality outcomes either as study outcomes or as treatment-emergent adverse effects [74-76].

#### 4.4.1 Randomised Controlled Trials

In an 8-week, randomised, double-blind, fixed-dose trial comparing fluvoxamine (150 mg daily, N = 20) and reboxetine (8 mg daily, N = 20) in terms of their efficacy in reducing motor-vehicle accident-related PTSD symptoms among 40 patients recruited from outpatient clinics, Spivak *et al.* [31] reported mild to moderate sedation as a side effect of fluvoxamine treatment (mild sedation, N = 1; moderate sedation, N = 1), and none of the patients in the fluvoxamine group reported insomnia. Both fluvoxamine and reboxetine were shown to be efficacious in reducing PTSD symptoms, but insomnia and sleep quality measures were not examined as study outcomes.

#### 4.4.2 Observational Studies, Case Series, and Case Reports

A prospective, uncontrolled, open-label 10-week trial [76, 77] examined the effectiveness of fluvoxamine monotherapy (mean dose 150 mg/day; range 100-250 mg/day) in male Vietnam combat veterans (n = 21, 45-65 years of age) with sleep disturbances due to chronic PTSD. 9 participants also met the criteria for major depression and 19 met the DSM-IV criteria for lifetime alcohol or substance misuse. Concurrent psychotherapeutic intervention did not change during the trial, and participants on concurrent treatment with other psychotropic medications prescribed for bedtime sedation (chloral hydrate or trazodone, N = 3, 14.3%) were excluded from the analysis for sleep quality, with data from 18 patients used in the analysis for sleep outcomes. Fluvoxamine was effective in all three clusters of PTSD symptoms (intrusion, avoidance, and hyperarousal symptoms) and also in subjective sleep quality, with significant improvement in self-reported sleep maintenance insomnia (item of the Impact of Event Scale-Revised - IES-R, effect size = 1.04, *p <* 0.01) and clinician-rated “troubled sleep” (item of the Stress Response Rating Scale - SRRS, effect size = 1.08, *p <* 0.01), but no significant differences in sleep onset insomnia (item of the IES-R) were observed. Furthermore, a significant improvement in self-reported nightmares resembling the trauma was identified (item of the IES-R, effect size = 1.11, *p <* 0.01), although scores for the SRRS item “bad dreams” did not change significantly. The authors reported excessive sleepiness as very low at baseline and it did not change during the trial [76, 77].

In another 12-week, open-label pilot study, De Boer *et al.* [74] examined a higher dosage of fluvoxamine monotherapy (300 mg/day, n = 24) in male veterans with chronic, resistant PTSD [74], and supported fluvoxamine’s effectiveness in the treatment of PTSD symptoms, including insomnia (as part of a self-rated PTSD symptom scale), nightmares, anxiety, intrusive recollections, guilt feelings, and tiredness [74]. Insomnia was among the symptoms that showed greater improvement. A subscale of the Dutch Subjective Sleep Quality of the last night was used to assess sleep quality, and there were no overall significant changes at 4 weeks and at endpoint (12 weeks). However, it is interesting that five out of the eleven 12-week completers insisted on continuing with fluvoxamine treatment after the end of the trial, and they all reported a marked improvement in their sleep quality. The drop-out rate was high (13 patients), but only one participant dropped out due to the deterioration of sleep quality. It is important to note that all psychotropics apart from benzodiazepines were discontinued 2 weeks prior to the trial, and it is not mentioned whether any changes were made to the concurrent benzodiazepine treatment during the trial. The authors also report that a proportion of patients (number not given) had a long history of psychoactive drug use, especially benzodiazepines, without a satisfactory outcome, which might imply benzodiazepine dependency at the time of the trial.

In contrast to the aforementioned results, Davidson *et al.* [75] reported high rates of insomnia as a side effect of fluvoxamine (46%, self-rated Medication Effects Scale) in an uncontrolled, open-label study among 15 civilians (9 included in the data analysis, 18-70 years of age) with non-combat related PTSD, prescribed fluvoxamine monotherapy (50-200 mg daily) over a period of 8 weeks. One patient dropped out due to insomnia. Insomnia and sleep quality measures were not specifically examined in terms of any potential therapeutic effect of fluvoxamine on these. Overall, fluvoxamine was reported to be effective in reducing PTSD symptoms (64.2% responders among completers).

Escalona *et al.* [78] also reported high rates of insomnia in a 14-week open-label clinical trial of fluvoxamine (mean dose = 150 mg, range = 100-300 mg daily) among a population of 15 Veterans with chronic combat PTSD. Temazepam and/or chloral hydrate were used as required for the treatment of insomnia, and it is reported that 73% of the participants utilised these drugs for the duration of the trial.

It is reported that fluvoxamine is not used as often as other psychotropic medications for the treatment of PTSD due to its side-effect profile relative to its efficacy [79]. It is true that in some of the aforementioned studies, the drop-out rates were considerable [74]; however, factors, such as motivation to engage in treatment and hopelessness, should be considered among chronic patients with severe PTSD, where comorbidity with major depression is high, failed treatment attempts and polypharmacy are common. Overall, among the aforementioned trials, fluvoxamine seemed reasonably well tolerated and in the only study examining a head-to-head comparison [31], the drop-out rate for reboxetine due to side effects was higher compared to the fluvoxamine group, but the difference was not statistically significant [21].

In summary, only 2 open-label studies [74, 76] report specifically on the therapeutic effects of fluvoxamine on insomnia and sleep quality among patients with PTSD, and they agree that fluvoxamine is effective in treating insomnia and improving the sleep quality. However, the quality of this evidence is poor, and potential confounding effects of concurrent benzodiazepine use are not fully accounted for [74]. Among studies that report on the side effect profile of fluvoxamine among PTSD patients, both insomnia and sedation have been identified (Table **4**).

### 4.5 Paroxetine

Paroxetine is a Selective Serotonin Reuptake Inhibitor (SSRI). It is one of the antidepressants with the best evidence to support its use for the treatment of PTSD symptoms [21, 44]. It is an FDA-approved treatment of PTSD, and it is one of the few licensed antidepressants for the treatment of PTSD in the United Kingdom, endorsed by the NICE guidelines [81]. Due to the robust evidence-base, and to remain relevant to the outcomes of this review, we only report results of studies that have examined sleep quality and disturbances among PTSD patients.

#### 4.5.1 Results From Pooled Analyses and Evidence From Systematic Reviews and Meta-analyses

In a pooled analysis of 3 similarly designed 12-week placebo-controlled clinical trials ([82, 83] SKB627 - unpublished data only [27], N = 1180, mixed civilian and combat PTSD, primarily civilian, mean age = 41, range = 18-75 years), Stein *et al.* [84] report that paroxetine 20-50 mg daily reduces sleep disturbance. This study does not provide numerical data, but cites a conference paper by Sheehan *et al.* [85], which reported results related to sleep disturbances using the same cohort of patients (data on sleep symptoms available for N = 1163, sleep items as part of Clinician-Administered PTSD Scale (CAPS-2), Montgomery-Åsberg Depression Rating Scale (MADRS), and Davidson Trauma Scale (DTS)). On both clinician and patient-rated instruments, paroxetine was found more efficacious than placebo in improving initial or maintenance insomnia (CAPS item 13, *p <* 0.001; DTS item 13, *p* = 0.002), and reduced overall sleep (MADRS item 4, *p* = 0.0021). Results were also positive for reducing severity of distressing dreams (CAPS-2 item 2, *p* = 0.053; DTS item 2, *p <* 0.01). No difference between paroxetine and placebo was identified in terms of insomnia as a treatment emergent adverse effect. However, numerical values were substantial: 11.8% for paroxetine *vs.* 11.3% for placebo.

The same studies included in the pooled analysis of Stein *et al.* [84] were also included in a pooled analysis reported in the attachment to the FDA approval letter for paroxetine (FDA20031s29lbl). In addition to the information provided by Stein *et al.* [84], the FDA attachment reports a substantial difference between paroxetine and placebo for somnolence as a treatment-emergent adverse effect: 16% for paroxetine (N = 676) *vs.* 5% for placebo (N = 504, total = 1180 patients).

In a recent meta-analysis examining effects of antidepressants on sleep quality among patients with PTSD [86], paroxetine was not found to be efficacious in improving sleep quality and reducing sleep disturbances, with similar acceptability to other antidepressants (sertraline and mirtazapine) and placebo. However, it is worth mentioning that only one small, unpublished clinical trial was included in the meta-analysis [87], which was terminated due to recruitment difficulties. Therefore, no firm, clinically relevant conclusions could be drawn from this meta-analysis.

#### 4.5.2 Results From Published and Unpublished Randomised Controlled Trials

In terms of formal assessment of sleep quality and sleep symptoms with validated scales, only one registered trial [87] was identified to have examined the following outcomes: CAPS-Recurrent Distressing Dreams item and PSQI. This was a double-blind, randomised, placebo-controlled parallel group 12-week clinical trial of prazosin (initial dose of 1 mg 4 times a day and titrated up to a maximum dose of 30 mg/day) *vs.* paroxetine 20 mg daily *vs.* placebo, among 59 participants (25 completers) with combat PTSD (18-50 years old).

The available study results are summarised in Table **5** below.

Although no statistical analysis is reported, numerical advantages of paroxetine over both prazosin and placebo can be observed. This study does not report on insomnia, somnolence or sleep disturbances, and parasomnias as treatment-emergent or withdrawal side effects.

All other identified RCTs do not report on sleep symptoms as primary or secondary study outcomes; they, however, report on sleep disturbances as treatment-emergent adverse effects.

In a 10-week randomised, placebo-controlled trial, Schneier *et al.* [38] recruited adult survivors of the World Trade Centre attack of September 11, 2001, with PTSD. They were randomly assigned to 10 weeks of treatment with Prolonged Exposure Therapy (10 sessions) plus paroxetine (N = 19, 49.1 ± 8 years old, 8 females, mean dose (SD) = 32.2 mg/day (13.4 mg)), or Prolonged Exposure Therapy plus placebo (N = 18, 51.5 ± 8 years old, 12 females). After week 10, Prolonged Exposure Therapy was discontinued, and participants were offered an additional 12 weeks of continued randomised pharmacotherapy. Only concurrent use of zolpidem for the treatment of insomnia was allowed during the randomisation phases. Insomnia was reported as a treatment-emergent side effect in one patient in the paroxetine group (5.26%) *vs.* 2 patients in the placebo group (11.11%) [88]. Somnolence was reported as a treatment-emergent adverse effect among 2 patients (10.53%) in the paroxetine group *vs.* 3 patients (16.67%) in the placebo group. No further information relevant to our study outcomes has been reported and no formal statistical analysis of the differences between the 2 groups in terms of treatment-emergent adverse effects was reported.

In another study of a relatively similar design, *i.e*., a 10-week randomised, placebo-controlled trial, Simon *et al.* [39] recruited adult outpatients with PTSD due to the following types of index trauma: abuse (physical/sexual), exposure to war (14%), physical accident or medical trauma (intent-to-treat sample N = 23). Following 8 sessions of individual Prolonged Exposure Therapy, they were randomly assigned to 10 weeks of treatment with an additional 5 sessions of Prolonged Exposure Therapy plus paroxetine (N = 9, 47.8 ± 11.4 years old, 44% females, mean dose (SD) = 45.8 mg/day (16.5 mg)), or Prolonged Exposure Therapy plus placebo (N = 14, 44.2 ± 15.9 years old, 64% females). Concurrent treatment with sedative and hypnotic medication (trazodone, zolpidem, zaleplon) was allowed but dosage remained stable during the randomisation phase. Sleep disturbance and drowsiness were among the most common treatment-emergent side effects for both paroxetine and placebo, and the numerical data reported are noteworthy for both paroxetine and placebo: 67% in the paroxetine group *vs.* 77% in the placebo group for drowsiness, and 89% in the paroxetine group *vs.* 85% in the placebo group for sleep disturbances.

Seo *et al.* [34] conducted a 10-week randomised trial comparing mirtazapine (N = 20, 38.1 ± 7.6 years old, 13 females, 43.93 ± 14.96 mg daily) and paroxetine (N = 20, 36.7 ± 9.1 years old, 15 females, 38.89 ± 10.23 mg daily) monotherapies among adults with civilian PTSD. No concurrent treatment with other psychotropic medications was allowed during the trial. They reported insomnia as a treatment-emergent side effect among 2 patients (10%) in the paroxetine group *vs.* no patient (0%) in the mirtazapine group. No further information relevant to our outcomes has been reported.

In an older but large 12-week randomised controlled trial [83] across 37 centres in the USA and Canada, paroxetine 20-50 mg daily (N = 151, mean age = 41.1, range = 19-69 years old, 33.8% males, mean dose 27.6 mg/day) was examined against placebo (N = 156, mean age = 39.8, range = 18-78 years old, 33.8% males), with 307 chronic and severe PTSD patients constituting the intent-to-treat population. Concurrent treatment with chloral hydrate for acute insomnia was allowed in doses up to 1000 mg daily for up to 3 nights per week during the first week of the randomised phase. Somnolence was reported as a common treatment-emergent adverse effect, observed among 17.2% of patients in the paroxetine group *vs.* 3.8% of patients in the placebo group. No other information relevant to our study outcomes has been reported.

#### 4.5.3 Important Results From Observational Studies

In a 10-week, open-label, prospective cohort study, Tucker *et al.* [89] assessed the effects of paroxetine (flexible dosage, 20-50 mg daily) monotherapy on subjective symptoms, autonomic reactivity, and diurnal salivary cortisol among 22 patients with PTSD and comorbid depression *vs.* 21 patients with depression alone *vs.* 20 healthy controls previously exposed to significant trauma. Previous antidepressant treatment was discontinued at least 2 weeks prior to enrolment (4 weeks for fluoxetine). Occasional use of diphenhydramine as a hypnagogic for at least 2 weeks was allowed. A variable of interest of this review was the hours of sleep (HSLEEP). Whilst HSLEEP scores did not differentiate comorbid PTSD and depression from depression alone, at the endpoint, HSLEEP improved significantly in the comorbid PTSD and depression group (N = 22, 68% of the population improved, *p* = 0.0003), and a relatively weaker improvement was observed in the patients with depression alone (N = 10, 60% improved, *p* = 0.0368); however, the number of patients with depression agreeing to switch to treatment with paroxetine was relatively limited (10 out of 21 patients in total), which may have limited the strength of the statistical analysis.

A summary of the results is presented in Table **6** below.

### 4.6 Sertraline

Sertraline is a commonly prescribed SSRI. There is robust evidence to support its efficacy in the treatment of depression [43]. Tolerability and side effect profile are also favourable compared to other antidepressants, including other SSRIs [44]. In clinical practice, it is traditionally the first choice for the treatment of depression in patients with comorbid heart disease due to the popular SADHART-CHF Trial results, proving its safety when prescribed for the treatment of depression among patients with heart failure [90].

In terms of treatment of PTSD symptoms, sertraline is among the antidepressants with the most robust evidence base for its efficacy and safety [21]. It is FDA-approved for the treatment of PTSD [91]; it is licensed in the UK for the treatment of PTSD, and endorsed by the NICE guidance [81]. However, paroxetine, fluoxetine, and other classes of antidepressants, for example, mirtazapine and phenelzine have been found superior in terms of efficacy for the treatment PTSD symptoms [45, 67].

It is interesting to note that mirtazapine is a sedative antidepressant, and phenelzine has been shown to reduce sleep disturbances among patients with depression, potentially due to its potent REM-suppressing effect [92]. REM sleep fragmentation and autonomic imbalance are associated with the development and persistence of PTSD symptoms [93]. In the sections of tricyclic antidepressants (TCAs) and phenelzine below, we present evidence supporting that improvement in sleep items on PTSD symptom scores is an important determinant of overall symptomatic improvement. Based on the above, it would be interesting to hypothesise that the presumed greater efficacy of some of the antidepressants above compared to sertraline could be attributed, perhaps at least partially, to their effects on sleep disturbances.

Although our study design does not allow an explicit and definitive exploration of the aforementioned hypothesis, we have endeavoured to present below all available evidence regarding the effects of sertraline on sleep outcomes among patients with PTSD, which may offer some insights and suggestions for further research.

#### 4.6.1 Results From Pooled Analyses and Evidence From Systematic Reviews and Meta-analyses

In a pooled analysis of two randomised controlled trials [94, 95], Davidson *et al.* [96] examined the effectiveness of sertraline (mean dose = 133.3-146.3 mg, range = 50-200 mg daily) *vs.* placebo on individual PTSD symptoms in a sample of 385 patients (predominately females with > 60% physical or sexual assault index trauma, N = 191 sertraline group and N = 194 placebo group). Insomnia was examined as part of the hyperarousal item scores, and it was found that there was a significantly steeper decline in scores signifying improvement over time in the sertraline group compared to placebo, with a relatively weak effect (F = 5.05, *p <* 0.05), but no statistically significant differences in scores between baseline and any individual point in time (weekly from baseline to endpoint at week 12) were identified. For nightmares (DTS re-experiencing item scores), there was no statistically significant difference between the groups at any point during the studies (F = 1.30, *p >* 0.05). No further data relevant to our study outcomes are discussed in this study.

In a recent network meta-analysis examining the effects of antidepressants on sleep quality among patients with PTSD [86], sertraline was the only antidepressant that was found possibly efficacious in reducing sleep disturbances, but not in improving sleep quality, with similar acceptability to other antidepressants (mirtazapine and paroxetine) and placebo. However, it is worth mentioning that the certainty of evidence is low, as a relatively small number of patients were included in the meta-analysis of 4 RCTs [33, 37, 95, 97].

#### 4.6.2 Evidence from Randomised Controlled Trials

##### 4.6.2.1 Studies Reporting on the Therapeutic Effects of Sertraline in Treating PTSD-related Sleep Disturbances and Improving Sleep Quality with or Without Data on Sleep-related Safety Outcomes

In a multicentre, 12-week double-blind, randomised, placebo-controlled clinical trial, Davidson *et al.* [95] examined the efficacy and safety of sertraline monotherapy (mean dose = 146.3 mg, range = 50-200 mg daily) in the treatment of PTSD symptoms among 208 patients with a variety of civilian and combat-related chronic PTSD (mostly females, mean age = 37.6 ± 11.1 years in the sertraline group (N = 100) *vs.* 36.6 ± 10.1 years in the placebo group (N = 108)). It is not clearly reported whether the concomitant use of rescue treatment for insomnia was allowed during the trial. Sleep quality was examined as a study outcome, using the Pittsburgh Sleep Quality Index (PSQI). Although the overall PSQI score did improve in the sertraline group, this improvement was not significantly greater than the improvement observed in the placebo group (t = 0.75, *p* = 0.45). In terms of sleep disturbances as treatment-emergent side effects, the incidence of insomnia was higher in the sertraline group *vs.* the placebo group (35% *vs.* 22%, *p* = 0.04), which was not observed for drowsiness (17% in the sertraline group *vs.* 11% in the placebo group, *p* = 0.24) and vivid dreams (10% in the sertraline group *vs.* 4% in the placebo group, *p* = 0.10).

Friedman *et al.* [97] also examined the efficacy and safety of sertraline monotherapy (N = 86, 68 males, 70% combat PTSD, mean dose = 135 mg, range = 50-200 mg daily) against placebo (N = 83, 67 males, 72% combat PTSD) among 169 outpatients using a 12-week double-blind, randomised clinical trial design. Concurrent treatment with chloral hydrate for insomnia was allowed during the trial. The PSQI was used to assess the therapeutic effects on sleep quality, and there were no significant differences observed between sertraline and placebo (ΔPSQI baseline *vs.* endpoint = -0.9 ± 0.4 in the sertraline group *vs.* -1.6 ± 0.4 in the placebo group, *p >* 0.05). Furthermore, there were no significant differences in insomnia as a treatment-emergent side effect between the groups (14% for the sertraline group *vs.* 9.6% for the placebo group, *p >* 0.05). Lastly, drowsiness also did not exhibit significant difference between the sertraline and the placebo groups (14% *vs.* 8.4% respectively, *p >* 0.05).

A study by McRae *et al.* [33] compared sertraline with nefazodone, a known sedative antidepressant. This was a double-blind, randomised, parallel-group 12-week clinical trial, comparing the efficacy, safety, and tolerability of sertraline (mean dose = 153 mg, max 200 mg daily) and nefazodone (mean dose = 463 mg, max 600 mg daily) monotherapy among 37 civilians with chronic PTSD (26 had at least one post-randomisation assessment, 23 completers at 12 weeks). There was no placebo arm. No concurrent rescue medication for insomnia was allowed during the trial. The PSQI was used to assess sleep quality as a secondary outcome. A significant improvement in PSQI score was identified for both sertraline and nefazodone, and no significant differences between the groups were identified. For the sertraline group (N = 13), the PSQI score dropped from 12.33 (SD = 4.9) at baseline to 7.00 (SD = 5.5) at the endpoint. For the nefazodone group (N = 13), the PSQI score dropped from 11.92 (SD = 5.9) at baseline to 6.83 (SD = 4.5) at the endpoint. Data on insomnia as a treatment-emergent side effect are also provided, with no significant differences between the 2 groups: 16.7% for the sertraline group (N = 18) *vs.* 21.1% for the nefazodone group (N = 19), *p >* 0.05. Furthermore, data on nightmares as treatment-emergent side effects also demonstrate no significant differences between the two groups, although numerical values tended to favour nefazodone (5.3% for the nefazodone group (N = 19) *vs.* 11.1% for the sertraline group (N = 18), *p >* 0.05). Interestingly, drowsiness as a treatment-emergent side effect did not differ significantly between the two groups as well: 27.8% for the sertraline group (N = 18) *vs.* 26.3% for the nefazodone group (N = 19), *p >* 0.05.

The Prolonged Exposure and Sertraline Trial (PROGrESS) [40] was a 24-week single-blind, randomised, parallel-group clinical trial with the following arms: (i) 24 weeks of sertraline + enhanced medication management (EMM), (ii) up to 13 sessions of prolonged exposure therapy (PET) + 24 weeks of sertraline and (iii) up to 13 sessions of PET + 24 weeks of placebo. The mean dose of sertraline was 171.6 mg daily (range 50-200 mg daily). 223 veterans (mean age = 34.5 ± 8.3 years, 87% males) with combat-related PTSD (69.1% comorbid major depression) were recruited in total, and 207 were included in the intent-to-treat analysis. Concurrent treatment with antidepressants or antipsychotics, benzodiazepines, prazosin, or sleep agents, such as zolpidem, was allowed during the trial if the dosage was stable for 2 weeks by the time of the trial commencement. For sleep outcomes, data are only available for a subgroup of 149 participants (sertraline + PET: N = 51; placebo + PET: N = 42; sertraline + EMM: N = 56), reported in another publication [40], which investigated the persistence of individual symptoms throughout treatment and at endpoint (week 24). They reported that difficulty sleeping (clinician-administered PTSD scale for DMS-IV, cluster D, symptom 1) and nightmares (clinician-administered PTSD scale for DMS-IV, cluster B, symptom 2) were among the most persistent symptoms at week 24 (76.6% and 63.7% of the total population of 149 patients, respectively). Importantly, they reported no significant differences between interventions in terms of the proportion of patients reporting persistent difficulty sleeping and nightmares, although numerical values tended to favour pharmacological intervention (sertraline), especially as an add-on to PET. Difficulty sleeping persisted at 24 weeks for 77.4% of 56 patients in the sertraline + EMM group *vs.* 82.1% of 39 patients in the PET + placebo group *vs.* 71.4% of 49 patients in the sertraline + PET group (*p* = 0.49). Nightmares persisted at 24 weeks for 58.5% of 56 patients in the sertraline + EMM group *vs.* 75% of 39 patients in the PET + placebo group *vs.* 60% of 49 patients in the sertraline + PET group (*p* = 0.29).

##### 4.6.2.2 Studies that Only Report on the Safety and Tolerability of Sleep-related Outcomes, Without Reporting on Therapeutic Outcomes

In a 12-week double-blind, randomised, parallel-group, placebo-controlled clinical trial, Brady *et al.* [94] examined the efficacy of sertraline monotherapy (mean dose = 133.3 mg, range = 50-200 mg daily) *vs.* placebo among 187 outpatients with moderate to severe chronic PTSD (mean age = 40 years, mean duration of illness = 12 years, 73% females, 61.5% physical or sexual assault). Therapeutic effects on relevant sleep outcomes have not been reported. However, they reported significant differences between sertraline and placebo in terms of treatment-emergent adverse effects, including insomnia and sedation. There was a statistically significant difference found in terms of insomnia in the F group (16%) *vs.* placebo (4.3%, *p* = 0.01). There was no statistically significant difference found between the two groups in terms of sedation as a treatment-emergent side effect (12.8% for the sertraline *vs.* 9.8% for the placebo group).

In another randomised clinical trial of 6 weeks duration, Chung *et al.* [32], examined the efficacy and tolerability of sertraline (N = 49, mean age = 60.6 years, mean dose = 101.5 mg daily) and mirtazapine (N = 51, mean age = 59.1, mean dose = 34.1 mg daily) among Korean veterans with chronic, moderate to severe combat PTSD. Although therapeutic effects on sleep outcomes were not directly examined, the authors reported that zopiclone 7.5 mg daily was allowed as rescue treatment for insomnia, which was used by 2 mirtazapine cases (3.4%) *vs.* 5 sertraline cases (9.1%). The authors did not report regarding direct comparisons among the side effect profiles of the two drugs. However, they reported somnolence as a common side effect of mirtazapine (15.7%), whereas insomnia was present in 2% of participants as a treatment-emergent side effect of sertraline treatment.

In another 12-week double-blind, randomised clinical trial by Davidson *et al.* [98], the efficacy and safety of venlafaxine extended-release monotherapy (N = 179, mean dose = 164.4 mg, range = 75-300 mg) were examined against sertraline monotherapy (N = 173, mean dose = 110.2 mg, range = 50-200 mg daily) and placebo (N = 179), among 531 adult patients with combat (9%) and civilian (91%)-related PTSD. Concurrent use of zaleplon or zolpidem, once at night as required for insomnia, for up to 6 nights, and only during the 14 days after the baseline evaluation, was allowed. Again, therapeutic outcomes on sleep parameters were not directly examined. However, data on sleep disturbances as treatment-emergent side effects have been reported. Specifically, there were no significant differences reported in insomnia rates between sertraline (10.4%), venlafaxine (13.4%), and placebo (8.9%), and drowsiness rates did not differ significantly between the groups (10.4% for sertraline, 11.7% for venlafaxine, and 13.4% for placebo).

In a smaller but more recent 12-week double-blind, randomised clinical trial from China, Li *et al.* [99] examined the safety and efficacy of sertraline monotherapy (N = 36, 47.1 ± 6.1 years old, 86.1% males, 38.9% “military-related trauma”, mean dose = 135 mg, range = 50-200 mg daily) *versus* placebo (N = 36, 44.9 ± 5.8 years old, 88.9% males, 36.1% “military-related trauma”) for the treatment of 72 patients with chronic PTSD. Therapeutic effects on sleep disturbances were not reported in this study; however, the authors have provided data on sleep disturbances as treatment-emergent adverse effects. Insomnia was reported by 27.8% of participants randomised to receive sertraline *vs.* 19.4% randomised to receive placebo (*p* = 0.41). Drowsiness was reported by 25% of participants in the sertraline group *vs.* 13.9% in the placebo group. Despite the numerical values indicating a difference, this was not statistically significant (*p* = 0.24).

Data from a large, unpublished 12-week double-blind, randomised, placebo-controlled clinical trial [26] are worth reporting here due to the study design, the large sample, and the rather smaller numbers of sleep-related disturbances as treatment-emergent side effects compared to all other studies mentioned above. The study included 4 arms: (i) experimental (N = 75): flexible dose of brexpiprazole monotherapy (0.5-3 mg daily); (ii) experimental (N = 82): combination therapy of brexpiprazole (0.5-3 mg daily) with sertraline (N = 82, 50-200 mg); (iii) active comparator (N = 81): sertraline monotherapy (N = 50-200 mg); and (iv) placebo comparator (N = 83). The total study population was 336 adult patients with PTSD, with an index trauma event <15 years before the screening. Although sleep quality measures were not studied as primary or secondary outcomes, insomnia and drowsiness have been reported as treatment-emergent adverse effects. For the sertraline monotherapy *vs.* placebo analysis, the incidence of insomnia was 4.9% for the sertraline group *vs.* 3.6% in the placebo group, and drowsiness was 3.7% *vs.* 8.4%, respectively. For the combination therapy analysis, the incidence of insomnia was 2.4% in the sertraline + brexpiprazole group *vs.* 6.7% in the sertraline + placebo group, and the incidence of drowsiness was 3.7% *vs.* 6.7%, respectively.

In another small, but relatively recent randomised clinical trial among Iranian veterans of the Iran-Iraq war with chronic combat PTSD [100], 35 patients were randomised to receive either sertraline (46.5 ± 5.4 years old, mean dose = 140 mg, 50-200 mg daily, 32 completers) or placebo (44.6 ± 5.1 years old, 30 completers) for a total of 10 weeks. As required, treatment with chloral hydrate or diazepam was allowed during the trial. As with most other studies available, only data on sleep disturbances as treatment-emergent side effects have been reported in this study, and the numerical values were substantial. Insomnia was reported among 28.6% of the participants in the sertraline group *vs.* 11.4% in the placebo group (*p* = 0.092). Drowsiness was reported among 14.3% in the sertraline group *vs.* 5.7% in the placebo group (*p* = 0.375).

An unpublished 12-week double-blind, randomised, placebo-controlled clinical trial [41] examined the efficacy of sertraline (fixed dose of 150 mg daily) prescribed as an adjunct to cognitive behavioural therapy (CBT) in the treatment of PTSD and alcohol use disorder dual diagnosis. 49 veterans were recruited (21-65 years old, N = 26 sertraline + CBT *vs.* N = 23 placebo + CBT). Data on therapeutic effects on sleep outcomes were not examined. However, data on sleep disturbances as treatment-emergent side effects have been reported, but there was no formal statistical analysis provided of these results. Insomnia was reported as a treatment-emergent side effect among 23.1% of participants in the sertraline + CBT group *vs.* 13% of participants in the placebo + CBT group. Furthermore, drowsiness was reported as a treatment-emergent side effect among 7.7% of participants in the sertraline + CBT group *vs.* 13% of participants in the placebo + CBT group. Lastly, nightmares have been reported as a treatment-emergent side effect among 19.2% of participants in the sertraline + CBT group *vs.* 21.7% of participants in the placebo + CBT group.

In an older 10-week double-blind, randomised, parallel-group, placebo-controlled clinical trial, Tucker *et al.* [28] examined the effects of sertraline monotherapy (N = 32, mean dose = 134.1 mg, range = 50-200 mg daily), citalopram monotherapy (N = 25, mean dose = 36.2 mg, range = 20-50 mg daily) and placebo (N = 10) on PTSD symptoms and autonomic reactivity to trauma cues among 58 patients (18-64 years old) with a mixture of civilian and combat-related PTSD. As required, treatment with diphenhydramine for sleep problems was allowed throughout the trial. Only data for insomnia as a treatment-emergent side effect have been reported: 43.5% for the sertraline group *vs.* 60% for the citalopram group *vs.* 70% for the placebo group, but no formal statistical analysis was performed.

In another 10-week double-blind, randomised, parallel-group, placebo-controlled clinical trial [101], the safety and efficacy of sertraline monotherapy (N = 23, mean age = 41 ± 6 years, mean dose = 120 mg, range = 50-200 mg daily) were examined *vs.* placebo (N = 19, mean age = 38 ± 9 years) among 42 veterans with chronic combat PTSD. As required, treatment with chloral hydrate or temazepam for sleep problems was allowed during the trial. Only data on drowsiness as a treatment-emergent side effect have been reported: N = 6 (26.1%) for the sertraline group *vs.* N = 3 (15.8%) for the placebo group, *p* = 0.477.

Studies on children and adolescent patients with PTSD are scarce, and it is rare that they report on sleep outcomes. One 10-week double-blind, randomised, parallel-group, placebo-controlled clinical trial [102] examined sertraline monotherapy (mean dose = 104 mg, range = 50-200 mg daily) for the treatment of PTSD symptoms among 129 children and adolescents (ages 6-17 years) with PTSD due to a variety of trauma, including sexual and physical abuse/violence, confronted with traumatic news, road traffic accident, witnessing violence, natural disasters, among others. Concomitant use of diphenhydramine or chloral hydrate for sleep problems was allowed during the trial. Although the study does not report on the potential therapeutic effects of sertraline in the treatment of insomnia or sleep disturbances, it does report on sleep-related treatment-emergent adverse effects, specifically insomnia, with no significant differences between sertraline and placebo [10.4% *vs.* 12.9% respectively, risk ratio = 0.81 (0.31, 2.10)].

##### 4.6.2.3 Evidence From Observational Studies

Very limited evidence regarding the long-term effects of sertraline on sleep-related outcomes is available from observational studies. Essentially, we found no observational study examining the therapeutic effects of sertraline on PTSD-related sleep disturbances. There was, however, some information found regarding tolerance and safety outcomes relevant to our study.

In a retrospective medical chart review of the dosage, effectiveness, and safety of sertraline (12.5-150 mg daily, most common doses 25-50 mg daily, median duration of treatment = 10.6 months) on a Japanese cohort of patients with PTSD caused by various types of trauma [103], the authors reported somnolence to be among the most common adverse events (N = 11, 9%), and one of the most common causes of discontinuation of treatment (3 patients, 2.45%).

##### 4.6.2.4 Summary of Results

Table **7** below presents a summary of the aforementioned findings among studies examining either therapeutic effects or adverse effects of sertraline on sleep outcomes in PTSD.

## 5 SEROTONIN AND NOREPINEPHRINE REUP-TAKE INHIBITORS (SNRIS)

### 5.1 Duloxetine

Duloxetine inhibits both the serotonin transporter (SERT) and the norepinephrine transporter (NET). Its noradrenergic actions are thought to contribute to its efficacy for somatic painful symptoms, and its pro-dopaminergic action in the prefrontal cortex is possibly responsible for its efficacy in treating cognitive symptoms of depression [104].

In a naturalistic open-label 8-week trial, duloxetine monotherapy (60-120 mg/day, prescribed after discontinuation of previous antidepressant treatment following a washout period of up to 6 days, without concurrent psychotherapeutic intervention during the trial) was found effective in treating PTSD symptoms, including nightmares and depressive symptoms in 21 veterans with treatment-refractory PTSD comorbid with major depression. 20 patients completed the study. However, 11 participants (55%) developed sleep disturbances, 13 (65%) experienced increased dream activity, and 10 (50%) experienced sleepiness/sedation, which were thought to be side effects of the treatment with duloxetine [105]. Insomnia and sleep quality measures were not specifically examined as study outcomes, and no further specific information is reported regarding the nature or the degree of the aforementioned side effects.

In a prospective open-label 12-week trial of duloxetine treatment (following discontinuation of any previous antidepressant treatment and a washout period of 1-2 weeks, mean dose 81 mg/day; range 30-120 mg/day) in 20 veterans with treatment-refractory PTSD, effectiveness was reported not only for PTSD symptoms but also for improvement of sleep quality [106]. The baseline mean score in the Pittsburgh Sleep Quality Index (PSQI) was 15.60 ± 0.71, whereas the corresponding endpoint mean score was 11.50 ± 0.87, and the improvement was statistically significant (*p <* 0.001). An important caveat, however, is that apart from duloxetine, patients were allowed to use lorazepam up to 3 mg/day for insomnia, agitation, and anxiety for the first two weeks. It is also reported that patients did not find duloxetine to have a sedative effect. It is, therefore, difficult to draw firm conclusions with regards to the effectiveness of duloxetine in improving sleep quality or treating insomnia. Side effects, such as insomnia, impairment of sleep quality, or sleepiness, were also not reported in this study.

Deterioration of PTSD symptoms in a dose-dependent fashion as a result of prescription of duloxetine was only identified in a case report [107], although significant psychiatric comorbidity (bipolar disorder, major depressive episode) and psychotropic polypharmacy were considered to limit the generalisability of the reported findings. Deneys and Ahearn [107], and another case report demonstrating the safety and effectiveness of a combination of duloxetine 60 mg daily, olanzapine 15 mg daily, and ECT in the treatment of comorbid major depression in a PTSD patient [108], did not report on the effects of treatment on insomnia or sleep quality.

In conclusion, there is direct [106] and indirect [105] evidence that duloxetine may be effective in treating insomnia and improving sleep quality in patients with PTSD, but the quality of the evidence is rather poor and no reliable conclusions can be drawn. Further studies are required with a design focused on insomnia and sleep quality indices among the study outcomes.

### 5.2 Venlafaxine

Venlafaxine blocks both the serotonin transporter (SERT) and the norepinephrine transporter (NET). The theoretical benefit of the dual monoamine mechanism may be supported by the evidence of dose-dependent unipolar antidepressant efficacy, possibly due to a dose-dependent increase in NET inhibition [109].

Among the non-SSRI antidepressants, venlafaxine is among the ones with the most robust evidence base in terms of its efficacy and safety for the treatment of PTSD symptoms [21, 44, 45].

However, as far as our study outcomes are concerned, there is limited literature examining the therapeutic effects of venlafaxine on sleep disturbances among patients with PTSD.

#### 5.2.1 Evidence From Pooled Analyses of RCTs and Systematic Reviews and Meta-analyses

In a pooled analysis of 2 placebo-controlled RCTs of venlafaxine’s extended-release (ER) [29, 98], Stein *et al.* [110] examined the onset of activity and time to response on individual CAPS-SX_17_ (17-item clinician-administered PTSD scale) items among N = 687 patients with PTSD. The mean dose of venlafaxine ER was 223.1 mg daily (range = 75-300 mg daily). The venlafaxine group comprised 340 patients (37.3% males), and a total of 340 patients (41.5% males) were included in the placebo group. Types of trauma included combat (39% and 38% among patients in the venlafaxine and the placebo groups, respectively), sexual assault/abuse, non-sexual assault, and unexpected death among others. Initial and maintenance insomnia, as well as nightmares, were examined as CAPS-SX_17_ items. There were no significant differences reported between the venlafaxine and the placebo groups at the endpoint (12 weeks) for both initial/maintenance insomnia (*p* = 0.076) and nightmares (*p* = 0.143). No data on sleep disturbances as treatment-emergent adverse effects have been provided.

#### 5.2.2 Evidence From Randomised Clinical Trials

In a multicentre, randomised, double-blind, placebo-controlled 24-week clinical trial, Davidson *et al.* [98] compared the efficacy of venlafaxine ER monotherapy (mean dose 221.5; range 75-300 mg/day; N = 161, 42.2 ± 12.09 years old, 44.7% males) and placebo (N = 168, 40.5 ± 13.01 years old, 47% males) in a sample of 329 patients with PTSD (diverse index trauma types, N = 20 combat in both groups). No information regarding the concomitant use of as-required treatment for sleep is provided. The authors report no data on the therapeutic effects of venlafaxine on sleep symptoms and sleep quality. However, they provide data on relevant sleep-related adverse effects. Insomnia has been reported as a treatment-emergent side effect among 12 patients (7.5%) in the venlafaxine ER group *vs.* 17 patients (10.1%) in the placebo group. 9 patients (5.6%) in the venlafaxine ER group and 9 patients (5.4%) in the placebo group reported somnolence as a treatment-emergent side effect. No formal statistical analysis was provided, and the numerical values did not appear to be significantly different between the groups.

In a 12-week double-blind, randomised clinical trial, Davidson *et al.* [29] examined the efficacy and safety of venlafaxine ER monotherapy (N = 179, mean dose = 164.4 mg, range = 75-300 mg) against sertraline monotherapy (N = 173, mean dose = 110.2 mg, range = 50-200 mg daily) and placebo (N = 179) among 531 adult patients with combat (9%) and civilian (91%)-related PTSD. Concurrent use of zaleplon or zolpidem, once at night as required for insomnia, for up to 6 nights, and only during the 14 days after the baseline evaluation, was allowed. Therapeutic outcomes on sleep parameters were not directly examined. However, data on sleep disturbances as treatment-emergent side effects have been reported. Specifically, there were no significant differences found in terms of insomnia between sertraline (10.4%), venlafaxine (13.4%), and placebo (8.9%), and drowsiness did not differ significantly between the groups (10.4% for sertraline, 11.7% for venlafaxine, and 13.4% for placebo).

Table **9** below summarises the aforementioned findings.

## 6 SEROTONIN ANTAGONIST AND REUPTAKE INHIBITORS (SARIS)

### 6.1 Nefazodone

Nefazodone is a serotonin antagonist and reuptake inhibitor (SARI) with robust 5-HT2A receptor antagonist actions and weaker 5-HT2C receptor antagonism and SERT inhibition. It has been used for the treatment of depression, panic disorder and aggressive behaviour [111]. However, it has been associated with liver toxicity, which commonly manifests as asymptomatic and transient elevation of serum aminotransferase levels. However, in some cases, nefazodone treatment can precipitate acute liver failure (incidence 1 per 250,000 to 300,000 patient-years of exposure) with associated mortality [111, 112]. Due to the aforementioned safety concerns, it is not widely used in clinical practice anymore, and has been withdrawn from the market in many countries, including the Netherlands and the United Kingdom, but it is still available in the United States for the treatment of refractory depression with a black box warning for hepatotoxicity [111].

Despite the safety concerns mentioned above, there is relatively more evidence regarding our study outcomes for nefazodone compared to other antidepressants. Furthermore, unlike SSRIs, nefazodone is unlikely to induce activation and associated insomnia and anxiety [113], and it has been shown to have a favourable beneficial effect on sleep compared to SSRIs and TCAs in sleep laboratory studies among patients with depression, demonstrated as decreased arousal and increased stage 2 sleep, but without suppression of REM sleep [114, 115]. Based on the above, we will discuss this drug and present the available evidence below.

#### 6.1.1 Pooled Analyses and Evidence From Systematic Reviews and Meta-analyses

In a pooled analysis of 6 observational trials [113], 105 outpatients with chronic, civilian, and combat (71.4%) PTSD treated with nefazodone mean doses of 272-583 mg daily (range = 50-600 mg daily) were included, of whom 92 entered the intent-to-treat analysis. In terms of sleep outcomes, “sleep troubles” as a component of cluster-D PTSD symptoms was examined, and a significant improvement was identified between baseline and endpoint (mean difference = 0.80 ± 1.51; t = 5.10; *p* = 0.0001). Furthermore, data on sleep items in the Hamilton Depression Scale (HDS) were available for 41 patients and were analysed. A statistically significant difference between baseline and endpoint HDS-sleep items scores was identified (mean difference = 2.24 ± 2.36; t = 6.11; df = 40; *p* = 0.0001), indicative of sleep improvement. In addition, the authors report unpublished data from a 10-week trial of nefazodone 100-600 mg daily including 10 patients with civilian PTSD [113], which demonstrated an improvement in sleep duration from 3.90h at baseline to 6.70 h at endpoint (*p <* 0.001). Lastly, nightmares were examined as an individual symptom belonging to Cluster-B PTSD symptoms, and a significant improvement was identified between baseline and endpoint (mean difference = 0.79 ± 1.36; t = 5.60; *p* = 0.0001). No data on insomnia, drowsiness/somnolence/sedation/hypersomnia as treatment-emergent side effects have been provided.

In a recent network meta-analysis examining the effects of antidepressants on sleep quality among patients with PTSD [86], nefazodone was not found to be efficacious in improving sleep quality and reducing sleep disturbances, with similar tolerability to sertraline. However, it is worth mentioning that only one small clinical trial was included in the meta-analysis [33], which was assessed as having high risk of bias. Therefore, no firm, clinically relevant conclusions can be drawn from this work.

#### 6.1.2 Evidence From Randomised Controlled Trials

To our knowledge, data on sleep outcomes are only available from one randomised controlled trial [33]. This was a double-blind, randomised, parallel-group 12-week clinical trial, comparing the efficacy, safety, and tolerability of nefazodone (mean dose = 463 mg, max 600 mg daily) and sertraline (mean dose = 153 mg, max 200 mg daily) monotherapy among 37 patients with chronic civilian PTSD (26 had at least one post-randomisation assessment, 23 completers at 12 weeks). There was no placebo arm. No concomitant rescue medication for sleep was allowed during the trial. The Pittsburgh Sleep Quality Index (PSQI) was used to assess sleep quality as a secondary outcome. A significant improvement in PSQI score was identified for both sertraline and nefazodone, and no significant differences between the groups were identified. For the nefazodone group (N = 13), the PSQI score dropped from 11.92 (SD = 5.9) at baseline to 6.83 (SD = 4.5) at the endpoint. For the sertraline group (N = 13), the PSQI score dropped from 12.33 (SD = 4.9) at baseline to 7.00 (SD = 5.5) at the endpoint. Data on incident insomnia as a treatment-emergent side effect have also been provided, with no significant differences between the 2 groups: 21.1% for the nefazodone group (N = 19) *vs.* 16.7% for the sertraline group (N = 18), *p >* 0.05. Furthermore, data on nightmares as treatment-emergent side effects also demonstrated no significant differences between the two groups, although numerical values tended to favour nefazodone (5.3% for the nefazodone group (N = 19) *vs.* 11.1% for the sertraline group (N = 18), *p >* 0.05). Interestingly, incident drowsiness as a treatment-emergent side effect did not differ significantly between the two groups as well: 26.3% for the nefazodone group (N = 19) *vs.* 27.8% for the sertraline group (N = 18), *p >* 0.05.

#### 6.1.3 Evidence From Observational Studies

Several observational trials have focused on outcomes relevant to our study and they report specifically on sleep quality indices.

In an open-label pilot study, Davidson *et al.* [116] examined the effectiveness of nefazodone (mean dose = 386 mg, max = 600 mg daily) among 17 civilians (45.7 ± 17.1 years-old, 4 males) with chronic PTSD in an outpatient setting (10 completers). Information on concurrent treatment with other psychotropic medications has not been provided, but the authors excluded patients who were unable to stop benzodiazepines, which might imply that concomitant use of benzodiazepines was not allowed. In terms of sleep outcomes, the effect of nefazodone was examined using two items extracted from the SIP (Structured Interview for PTSD). On the item of general sleep disturbance, there was a 61.5% response rate at 2 weeks, with a small increase to 62.5% at 12 weeks. The response rate was defined as ≥50% drop in the score. On the item used to examine nightmares, there was a 58.3% response at week 2 and, interestingly, the response rate was reduced to 50% at week 12 among completers. Adverse effects were examined at the endpoint based on the difference in intensity compared to the baseline. Drowsiness has been reported as a treatment-emergent side effect among 31.3% of completers.

Gillin *et al.* [117] conducted a 12-week open-label trial of nefazodone monotherapy (mean dose = 441 mg, max = 600 mg daily) to examine the effects of the drug on polysomnographic sleep measures, sleep quality, nightmares, and other PTSD symptoms, among 12 male veterans with chronic combat PTSD comorbid with major depressive disorder (all but 1 patient met DSM-IV criteria for MDD). No information on the concurrent use of rescue sleep medication has been provided, but the authors stated that all patients were drug-free for a period of at least 2 weeks prior to enrolment. Polysomnographic measures, including sleep latency, total sleep time, sleep efficiency, and REM indices, did not change significantly between baseline and endpoint. However, there were significant improvements in the following sleep outcomes: sleep quality, measured using the PSQI (graphs but no exact numerical values are provided, Wilcoxon S = -37.5, *p* = 0.006; mixed model: t = -3.6, df = 32, *p* = 0.001); the number of nights with nightmares per week (graphs but no exact numerical values are provided, Wilcoxon S = -25, *p* = 0.03; mixed model: t = -2.19, df = 108, *p* = 0.03); and number of nightmares in the past week (graphs but no exact numerical values are provided, Wilcoxon S = -25, *p* = 0.02; mixed model: t = -2.14, df = 106, *p* = 0.03). Although no detailed data on sleep-related treatment-emergent side effects have been provided, the authors reported one patient to develop hypersomnia (reported sleeping 14-18 hours/day) while on nefazodone 400 mg daily, which improved significantly when the dose was reduced to 200 mg daily. Other patients also reported transient daytime sedation requiring dose reduction. However, the authors report that no effect was observed on the Epworth scale (a subjective measure of weekly daytime somnolence) for the entirety of the trial completers.

In another 12-week, open-label trial of nefazodone monotherapy (mean dose 490 mg, range = 300- 600 mg daily) among 10 patients (mean age = 46, range = 31-51 years old) with moderate to heavy symptoms of combat PTSD, followed by a 4-week follow-up (N = 9), Hertzberg *et al.* [118] examined the effectiveness of nefazodone for PTSD symptoms and included sleep outcomes. The concomitant use of other psychotropic medications, including sleep rescue treatment, was not allowed. Total sleep time was one of the study outcomes, and significant improvement was demonstrated between baseline and endpoint at 12 weeks (6.8 ± 1.4h at 12 weeks *vs.* 4.4 ± 0.9h at baseline, effect size = 1.73, F = 30.08, *p* = 0.0004), which was maintained at the 16-week follow-up (N = 9, 6.7 ± 1.6h, effect size = 1.27, F = 14.47, *p* = 0.005 *vs.* baseline). Sleep quality was examined using the PSQI score and significant improvements were identified: mean PSQI score = 13.2 ± 2.8 at baseline *vs.* 7.7 ± 4.3 at week 12 (effect size = 1.44, F = 20.65, *p* = 0.001), with a slight further improvement at the 16-week follow-up (mean PSQI score = 6.7 ± 5.2, effect size = 1.27, F = 14.47, *p* = 0.004 *vs.* baseline). No data on sleep-related treatment-emergent side effects have been provided.

The same authors also conducted a 3-4 year follow-up study on the same cohort [119]. All 10 patients continued with nefazodone and completed the 3-4-year follow-up. 6 patients maintained their daily dosage, but for 3, the entire daily dose was shifted to night-time to improve sleep. For 4 patients, nefazodone dose was increased to 600 mg daily (maximum dose) and was also shifted to night-time. Since the completion of the 12-week trial, the majority of patients had concurrent psychotherapy, and only 3 were maintained on pharmacotherapy. 6 patients were maintained on nefazodone monotherapy. The remaining 4, were also prescribed add-on pharmacotherapy, including clonazepam (1 patient), hydroxyzine (1 patient), bupropion (1 patient), carbamazepine, and venlafaxine (1 patient). Compliance with nefazodone was reported to be high, and 9 patients expressed the desire to remain on maintenance treatment in the long term. Improvements in PSQI score were maintained with good effect sizes compared to baseline (N = 10, mean PSQI score = 13.2 ± 2.8 at baseline *vs.* 9.2 ± 3.3 at 3-4 years follow-up, effect size = 1.13, F = 12.81, *p* = 0.0059), but numerical values indicated a slight deterioration compared to the 12-week and 16-week endpoints (presented above). Similarly, improvements in total sleep time were maintained at 3-4-year follow-up, but again, a slight deterioration was observed compared to the 12-week endpoint (N = 10, 5.8 ± 1.1 h at 3-4 years follow-up *vs.* 4.4 ± 0.9 h at baseline, effect size = 0.94, F = 8.85, *p* = 0.0156). No subgroup analysis examining the effects of psychotherapy or additional pharmacotherapy was preformed, perhaps due to the small number of participants.

Another study reporting on sleep-related outcomes examined the effects of nefazodone monotherapy (mean dose 272.5 mg, range = 125-500 mg daily) on total sleep time and dream-related measures among 15 patients with chronic PTSD (13 male veterans with combat PTSD, comorbid major depression in the majority, 11 completers), using a 6-week open-label, uncontrolled clinical trial design [120]. The concurrent use of other psychotropic medication was not allowed during the trial. The total sleep time (assessed *via* a self-reported morning diary) was found to be significantly improved at week 1 compared to baseline (372.5 ± 82.6 minutes at week 1 *vs.* 309.1 ± 161.4 at baseline, *p <* 0.01), with numerical data indicating some deterioration but still improved total sleep time compared to baseline at week 6 (343.4 ± 88.8 minutes, no formal statistical analysis data are provided). Furthermore, some improvement in dream-related distress was found at both weeks 1 and 6, but this did not reach statistical significance, probably because of the limited number of participants returning responses with relevant data on this outcome (N = 7 baseline, N = 8 at 1 week, N = 5 at 6 weeks). Lastly, dream similarity (assessed using a Likert scale to capture the similarity between the dream content and the traumatic experience) was significantly decreased between baseline and week 6 [2.3 ± 1.7 at baseline (N = 7) *vs.* 0 at 6 weeks (N = 5), *p* < 0.0^1^]. Sedation was reported as a potentially treatment-emergent side effect among the reasons for dropping out of the study, but the number of participants experiencing this has not been reported.

In a 12-week open-label clinical trial, Neylan *et al.* [121] examined the effects of nefazodone monotherapy (mean dose = 570 mg, range = 500-600 mg daily) on PTSD symptoms and sleep quality in a population of 10 male Vietnam combat veterans with chronic PTSD (54.1 ± 5.3 years old). No concurrent use of other psychotropic medication was allowed during the trial. Objective sleep quality was assessed with ambulatory polysomnography and subjective sleep quality was assessed using the PSQI. Furthermore, the effect on nightmares was assessed using an item of the IES-R (Impact of Event Scale-Revised) capturing trauma-specific dreams. In contrast to the Gillin *et al.*’s study mentioned above [117], there were significant improvements reported in objective sleep quality. The total sleep time assessed *via* polysomnography was reported to be significantly improved at 12 weeks compared to baseline (324 ± 75 minutes at baseline *vs.* 460 ± 56 minutes at 12 weeks, effect size = 1.95, t = -5.5, *p* = 0.001). Sleep maintenance was also reported to be significantly improved: 71.3 ± 15.6% at baseline *vs.* 91.7 ± 7.6% at week 12 (effect size = 1.11, t = -3.2, *p* = 0.016). Furthermore, there was an improvement reported in the duration of stage 2 sleep from 165 ± 48 minutes at baseline to 256 ± 101 minutes at week 12 (effect size = 1.17, t = -3.3, *p* = 0.013). There were also significant effects on quantitative delta sleep analysis [period amplitude analysis (PAA) measures], including higher integrated amplitude (effect size = 2.14, t = -6.1, *p* = 0.001), higher time in band (effect size = 1.87, t = -5.3, *p* = 0.001), higher number of half waves (effect size = 1.46, t = -4.1, *p* = 0.004), higher integrated amplitude of delta in NREM3 (effect size = 1.22, t = -3.2, *p* = 0.018) and in NREM4 (effect size = 1.17, t = -2.9, *p* = 0.035). In terms of subjective sleep quality, there was a significant improvement observed in the total PSQI score at 12 weeks compared to baseline (14.3 ± 2.9 at baseline *vs.* 10.9 ± 2.7 at 12 weeks, effect size = 1.17, t = 3.7, *p* = 0.005). Lastly, there was also a significant improvement reported in the IES-R nightmare item score, which dropped from 3.5 ± 1.2 at baseline to 2.1 ± 0.9 at 12 weeks (effect size = 1.43, t = 4.3, *p* = 0.003). Data on treatment-emergent adverse effects have not been reported.

Zisook *et al.* [122] conducted an open-label design study with a duration of 12 weeks to examine the effects of nefazodone monotherapy (mean dose = 424 mg, range = 100-600 mg daily) on depressive and PTSD symptoms among 19 male veterans with chronic combat PTSD, refractory to treatment with at least 3 full trials of other antidepressants. Sleep quality was assessed using the PSQI and data on the PSQI sub-scores for 17 patients have beenprovided. Mean hours of sleep per night were reported to be increased significantly at the end-point compared to baseline, from 5h to 5.9h (t = 4.20, df = 15, *p <* 0.01). There was also an improvement reported in initial insomnia (“being able to fall asleep”, t = 3.07, df = 15, *p <* 0.01). Measures of maintenance insomnia also improved significantly (t = 2.72, df = 15, *p <* 0.05). The number of distressing dreams/nightmares also showed a significant reduction at the endpoint compared to the baseline (t = 2.49, df = 15, *p <* 0.05). Lastly, self-reported overall quality of sleep was also found to be significantly improved (t = 3.31, df = 15, *p <* 0.01). The authors also reported on treatment-emergent adverse effects. Drowsiness was reported as a common adverse effect (N = 7, 37%).

Table **10** below summarises findings from all types of studies described in detail above.

### 6.2 Trazodone

From a pharmacological point of view, trazodone is the prototype serotonin antagonist and reuptake inhibitor (SARI). It blocks 5-HT2A and 5-HT2C receptors, as well as serotonin reuptake. It has also been shown to act as an antagonist at 5-HT1D, 5-HT2B, and 5-HT7 receptors. It is a potent antagonist at the α_1B_, α_1A_, α_2C_, and α_2B_ receptors, H_1_ histamine receptors, and also has agonist actions at 5-HT1A receptors [123]. Due to its high affinity for the α1 subtypes and 5-HT2A receptors clinically translating to a robust sedative-hypnotic effect, low-dose trazodone is a popular treatment for insomnia either as monotherapy, or as an add-on to SSRIs/SNRIs, especially when the latter have precipitated deterioration or emergence of insomnia as an adverse effect or residual insomnia remains problematic and is not adequately responding to the aforementioned agents [124, 125].

There is very limited evidence to support the use of trazodone for the treatment of PTSD symptoms [21]. However, the majority of available studies report on outcomes relevant to our review, albeit the quality of evidence is very low. All studies are observational and reporting of the results is of suboptimal quality in many cases.

In a 3-month open-label uncontrolled trial, Ashford *et al.* [126] examined the effects of trazodone (25-500 mg daily) on sleep parameters (subjective, non-standardised ratings of sleep quality, dreams and nightmares) among 57 individuals exposed to war trauma (N = 29 with PTSD diagnosis, N = 20 under age of 60 with PTSD diagnosis, 19 returned results, and N = 10 over the age of 60 with PTSD diagnosis who returned results). They mention that a total of 28 (93%) PTSD patients benefited from the drug, reporting improved initial insomnia, better sleep, or fewer nightmares. The authors reported improvement among 19 (100%) of the younger PTSD patients (below the age of 60) in terms of sleep quality (better sleep) and complete resolution of nightmares among 7 of the younger patients (37%) with the rest reporting an improvement of 75% (nightmares are “at least 75% better”). Among the older patients, 33% reported complete resolution of nightmares and 33% reported better sleep quality. It is worth noting that concomitant use of benzodiazepines and perhaps other psychotropic medication is implied but not reported in detail or quantified. The authors also report on sleep-related adverse effects, but these are not quantified for the patients with PTSD diagnosis specifically.

In another open-label trial, Hertzberg *et al.* [127] recruited 6 veterans (mean age = 46, range = 43-48) with chronic combat PTSD and comorbid major depression, who were treated with trazodone (mean dose = 300 mg daily, range = 50-400 mg daily). The design involved a quasi-experimental control arm, resembling a waiting list condition. Patients were divided into 2 groups (N = 3 each). Group 1 (N = 3) participants were commenced on trazodone at baseline and post-treatment evaluation was completed at 4 months. Group 2 participants (N = 3) were commenced on trazodone 2 months after the baseline evaluation (untreated for the first 2 months, quasi-waiting list condition), and post-treatment assessments were completed 4 months later (time lag of 2 months between the 2 groups). During the 2-month time lag (baseline to the first 2 months), monthly evaluation of both the treated (group 1) and the untreated participants (group 2) took place. Concomitant use of other psychotropic medication was not allowed for the whole duration of the trial. Sleep quality was examined using the PSQI and data on total sleep time are also available. During the first 2 months, no significant improvements in PSQI scores have been reported for group 2 (N = 3, untreated, quasi-waiting list condition, PSQI_baseline_ = 12.7 *vs.* PSQI_1-month_ = 12.3 *vs.* PSQI_2-months_ = 12.3, no formal statistical analysis reported). Similarly, no significant improvement in total sleep time was identified for the untreated group 2 for the first 2 months (quasi-waiting list condition, total sleep time = 4h at baseline *vs.* 3.5h at 1 month *vs.* 3.2h at 2 months; no formal statistical analysis has been reported). To evaluate the effects of treatment, data from the total number of participants during the time they received treatment (baseline to the endpoint at 4 months of treatment) were analysed together. A significant improvement in PSQI scores was identified (N = 6, PSQI_baseline_ = 12.8 ± 1.8 *vs.* PSQI_endpoint (4 months)_ = 7.8 ± 3.1; no formal statistical analysis was reported). Furthermore, a significant improvement in total sleep time has been reported (N = 6, 3.8 ± 1h at baseline *vs.* 6.0 ± 1.4h at endpoint (4 months); no formal statistical analysis was provided). The authors report that sleep symptoms were the first to improve within the first 2-3 months for all participants, and a continuous improvement was identified throughout the treatment period between baseline and endpoint. In addition, 2 participants from group 1 and 3 from group 2 were evaluated 1 month and 3 months after the endpoint to assess the maintenance of benefit at follow-up. Data from all 5 participants were analysed together. Improvements in PSQI scores tended to persist (N = 5, PSQI_follow-up_ = 8.8 ± 4.6, no formal statistical analysis comparing baseline, endpoint, and follow-up), and improvement in total sleep time was maintained (N = 5, total sleep time at follow-up = 5.7 ± 1.6 h, no formal statistical analysis comparing baseline, endpoint, and follow-up). It is worth noting that depressive symptoms were also evaluated throughout the study using the Beck’s Depression Inventory (BDI), and no significant improvement in depressive symptoms was identified. The authors report that the improvement of PTSD symptoms observed, including sleep disturbances, may not be attributable to the antidepressant effects of trazodone. The reliability and generalisability of this conclusion, however, are clinically questionable due to the very limited number of participants and the lack of formal statistical analysis. Lastly, the authors reported trazodone to be well-tolerated, but they did not provide any information regarding specific treatment-emergent adverse effects.

In another study, Warner *et al.* [128] examined the effects of adjunctive trazodone treatment (mean dose 212 mg/day, most patients used 50-200 mg daily, range = 25-600 mg daily) on sleep disturbances among 74 male veterans (N = 60 completers, mean age = 50, range = 28-60 years) with chronic combat PTSD (92% comorbid major depression), who completed an 8-week inpatient treatment program. 97% were on other antidepressants (fluoxetine, paroxetine, sertraline nefazodone), 28% were on valproic acid, 13% on benzodiazepines, and 10% on antipsychotics (olanzapine, risperidone). Effects on frequency and intensity of nightmares and insomnia (helpfulness with sleep), but also sleep-related treatment-emergent adverse effects, were examined retrospectively, using a questionnaire, which was empirically developed by the authors. 100% (N = 60) of the completers reported that trazodone helped with overall sleep, 92% (N = 55) reported that it helped with initial insomnia, and 78% (N = 47) reported that it was helpful with maintenance insomnia. Trazodone was used for the treatment of nightmares, specifically among 55 patients. 73% of them (N = 40) reported moderate to significant improvement in nightmares. The authors also conducted the statistical analysis of these data. They performed Pearson correlation tests with 3 variables: dose, helpfulness with nightmare (NM help) rating, and helpfulness with sleep (SLP help) rating. They identified significant correlations between NM help and SLP help correlation (r = 0.57, *p <* .005, N = 55), but no significant correlations were identified between dose and either NM help (r = -0.01, N = 55) or SLP help (r = 0.16, N = 60). The frequency of nightmares (nights per week) was compared pre- and post-treatment with trazodone, and a significant reduction was identified (mean frequency pre-treatment = 3.3 ± 1.7 *vs.* 1.3 ± 1.4 post-treatment, t = 9.7, df = 54 and *p <* 0.005). In terms of treatment-emergent adverse effects, the authors reported that 14 patients had to discontinue trazodone due to intolerable adverse effects. 36% (N = 5) discontinued due to daytime sedation and 6.7% (N = 1) discontinued due to vivid nightmares, although it was not reported whether these dreams had any resemblance to the index trauma. For the 60 completers, the authors reported 26 (43.3%) to experience daytime drowsiness, which was found to be tolerable.

Table **11** below summarises the aforementioned findings.

## 7 NORADRENERGIC REUPTAKE INHIBITORS (NARIs)

### 7.1 Reboxetine

Reboxetine is a selective noradrenaline reuptake inhibitor (NaRI), and it was the first drug reported in this class of antidepressants [129]. It is not commonly used in clinical practice, perhaps due to evidence suggesting that it is among the least efficacious drugs for the treatment of depression [43].

There is paucity of evidence regarding reboxetine’s efficacy and safety for the treatment of PTSD symptoms with only one head-to-head trial comparing reboxetine and fluvoxamine [80]. This trial did not examine the therapeutic effects on sleep quality or other sleep-related outcomes, but it did provide data on treatment-emergent side effects, which are relevant to our study outcomes.

The trial conducted by Spivak *et al.* [80] was an 8-week, randomised, double-blind, fixed-dose trial comparing fluvoxamine (150 mg daily, N = 20, 17 completers) and reboxetine (8 mg daily, N = 20, 11 completers) in terms of their efficacy in reducing motor-vehicle accident-related PTSD symptoms among 40 patients recruited from outpatient clinics. The authors reported increased sleep duration as a mild treatment-emergent adverse effect of reboxetine in 1 patient (9.1%), reduced sleep duration as a moderate treatment-emergent adverse effect of reboxetine in 4 patients (36.4%), and insomnia as one of the reasons for dropping out of the trial, although they did not mention the number of patients dropping out because they developed insomnia. There was no head-to-head comparison provided of side effect profiles with a formal statistical analysis. It is, however, worth mentioning that fluvoxamine was reported to be overall better tolerated than reboxetine, including in terms of sleep-related adverse effects.

Table **12** below summarises the findings discussed above.

## 8 TRICYCLIC ANTIDEPRESSANTS (TCAs)

### 8.1 Amitriptyline

Amitriptyline is a TCA known to inhibit serotonin and norepinephrine reuptake and to act as an antagonist at H1 (histaminic), α1 (adrenergic), and muscarinic cholinergic receptors as well as blocking voltage-sensitive sodium channels [130]. Low-dose amitriptyline is commonly used for the treatment of sleep disturbances in clinical practice, especially in primary care, and it is perceived by prescribers as an effective long-term option for the treatment of primary and secondary insomnia [131]. However, robust evidence to support this line of practice is lacking [132].

The evidence regarding the use of amitriptyline for the treatment of insomnia and improving sleep quality in patients with PTSD is presented below (Table **13**).

#### 8.1.1 Evidence From Randomised Controlled Trials

Only one randomised, double-blind, placebo-controlled 8-week trial studied the efficacy of amitriptyline in treating PTSD symptoms in a sample of 46 veterans with chronic PTSD [133]. 40 (87%) of 46 patients completed the minimum 4 weeks of treatment that was required for inclusion in the efficacy analysis. 35 patients (76%) completed 6 weeks of treatment and 33 (71%) completed 8 weeks of treatment. Although insomnia and sleep quality were not examined as primary or secondary outcomes, a multivariate analysis of the data obtained from this trial [134] identified insomnia as among the symptoms taken from the Hamilton Depression/Hamilton Anxiety Scales, which were found to be drug-responsive [changed scores for amitriptyline *vs.* placebo = 1.31 *vs.* 0.38 (t = 3.16, df = 53, *p* = 0.002)]. The reported dropouts due to tolerance issues were limited (2 earlier than 4 weeks, 1 at weeks 4-6 in the amitriptyline group), with a total of 3 dropouts due to adverse effects. Insomnia was reported as a treatment-emergent side effect among 4% of participants in the amitriptyline group *vs.* 22% in the placebo group. Drowsiness was reported as a treatment-emergent adverse effect among 67% of participants in the amitriptyline group *vs.* 50% in the placebo group.

#### 8.1.2 Observational Studies and Case Reports

In a small observational study, Falcon *et al.* [135] used clinical data from a mixed inpatient and outpatient cohort of 20 veterans with combat PTSD. They identified 10 patients who were treated with amitriptyline 150-250 mg daily for 6-8 weeks and reported improvement of nightmares along with other intrusion symptoms, although they did not report on outcomes, such as insomnia and sleep quality.

In another small, retrospective cohort study of the medical records in a specialist outpatient clinic, Bleich *et al.* [136] reported moderate to good response of a mean dose of amitriptyline 139 mg daily for 2-24 weeks (mean = 6.5 months) in terms of improvement of sleep impairment in 13 out of a total of 14 Israeli veterans with severe combat PTSD treated with amitriptyline, although no validated rating scales were used. Some of the patients in this study were also receiving either dynamic or supportive psychotherapy, and it is not clear how many patients on amitriptyline were receiving this at the time of the study, but the authors reported improvement in psychotherapeutic outcomes attributable to concurrent pharmacotherapy. Lastly, one patient on amitriptyline was also receiving augmentation therapy with methotrimeprazine (50-75 mg/day).

Başoǧlu *et al.* [137] reported a case of a 29 year-old man with severe insomnia among other symptoms of severe torture-related PTSD, who had refused psychotherapy and was treated with amitriptyline monotherapy 150 mg daily and followed up for 8 months. A marked improvement in insomnia (although not quantified using specific sleep quality outcomes), along with all clusters of PTSD symptoms, was reported, including the disappearance of nightmares, in a continuous fashion over the first 6-8 weeks of treatment, and recurrence of severe insomnia was reported following a 2-week discontinuation of treatment 6 months after the treatment had been started, which disappeared quickly after re-trial of amitriptyline and improvement was maintained in the long-term.

Table **13** below provides a summary of the findings discussed above.

### 8.2 Clomipramine

Clomipramine is a tricyclic antidepressant (TCA). It acts as a strong serotonin re-uptake inhibitor, with a stronger affinity for the serotonin transporter (SERT) compared to most other TCAs and some SSRIs. Its active metabolite desmethyl clomipramine has more noradrenergic activity. α1 receptor blockage and β receptor down-regulation have also been reported, as well as the potential blockade of sodium channels [138].

There is very limited evidence related to the use of clomipramine in PTSD. Sleep quality or insomnia have not been specifically examined as treatment outcomes.

In a small, retrospective cohort study of the medical records in a specialist outpatient clinic, Bleich *et al.* [136] examined 5 different TCAs for the treatment of PTSD among Israeli veterans with severe combat PTSD. Only 2 patients treated with clomipramine were identified. One patient had a poor response, and one exhibited a moderate response to clomipramine 150 mg daily (duration of treatment not specified, but ≥ 2 weeks). The authors reported sleep to be among the symptoms with the greatest improvement in patients showing moderate response to treatment, although no validated rating scales were used. Some of the patients were also receiving either dynamic or supportive psychotherapy, and it is not clear how many patients on clomipramine were receiving this at the time of the study, but the authors reported improvement in psychotherapeutic outcomes, which may be attributed to concurrent pharmacotherapy overall.

In another small cohort study, Chen [139] examined clomipramine (100-150 mg/day) in a sample of seven Vietnam veterans who were inpatients and reported improvement in their intrusive and obsessive symptoms. However, there was no reference provided to insomnia or sleep quality outcomes.

Both studies did not report on insomnia, deterioration of sleep quality parameters, or sleepiness as side effects of clomipramine.

All in all, there was essentially no evidence to support the prescription of clomipramine for the treatment of insomnia or improvement of sleep quality in patients with PTSD (Table **14**).

### 8.3 Desipramine

Desipramine is probably more selective for NET inhibition and has more notable NRI action as well as a lower affinity for muscarinic receptors and fewer interactions with other drugs (even when co-prescribed with SSRIs or MAOIs) compared to other TCAs. These characteristics make the drug pharmacologically more desirable compared to other TCAs [138]. However, desipramine is not commonly used in clinical practice as it is associated with greater than average cardiac toxicity and pro-convulsant effects [140]. Desipramine is an REM sleep suppressor and it is used off-label for the treatment of REM sleep behaviour disorder [140].

There is a paucity of literature examining the effects of desipramine on PTSD symptoms, and even fewer studies have examined the effects of the drug on sleep among patients with PTSD.

We have only identified a small observational study, [135] utilising clinical data from a mixed inpatient and outpatient cohort of 20 veterans with combat PTSD. The authors have identified 7 patients who have been treated with desipramine 200-250 mg daily for 6-8 weeks and reported improvement of nightmares along with other intrusion symptoms; however, they did not report on any other of our study outcomes (Table **15**).

### 8.4 Doxepin

Among tricyclic antidepressants (TCAs), doxepin has the highest affinity for the histamine-1 (H1) receptor, and at low, sub-therapeutic antidepressant doses, it is a relatively selective H1 antagonist [141]. It is, therefore, a drug that has clinical utility for the treatment of insomnia [124]. At higher doses (150-300 mg daily), serotonin and norepinephrine reuptake and anticholinergic properties are clinically significant, and it acts as a typical TCA in terms of receptor profile, with significant sedative properties.

There is very limited evidence supporting the use of doxepin for the treatment of PTSD. However, a significant proportion of the limited published literature has examined sleep-related outcomes. All studies are old, observational, with a very small number of participants, and suboptimal reporting of outcomes. We discuss this evidence below.

In a small, retrospective cohort study of the medical records of patients treated in a specialist outpatient clinic, Bleich *et al.* [136] reported moderate to good response to a mean dose of doxepin of 100 mg daily for 2-24 weeks (mean = 6.5 months) in terms of improvement of sleep impairment in 4 out of a total of 7 Israeli veterans with severe combat PTSD, although no validated rating scales were used. Some of the patients were also receiving either dynamic or supportive psychotherapy, and it is not clear how many patients on doxepin were receiving this at the time of the study, but the authors have reported improvement in psychotherapeutic outcomes attributable to concurrent pharmacotherapy. Lastly, concurrent treatment with antipsychotics (methotrimeprazine, thioridazine, sulpiride, and fluphenazine among others) has been reported, but it is not clear whether any patients on doxepin were receiving any of these agents.

In a retrospective review of clinical notes of 12 patients one year after receiving a diagnosis of PTSD (survivors of Cambodian concentration camps), Boehnlein *et al.* [142] report that 6 patients received treatment with doxepin (50-150 mg daily, 4 monotherapy, 2 on combination with other TCAs and phenelzine), which resulted in normalisation or improvement of sleep patterns and insomnia among 83% of these patients, and disappearance or significant improvement of nightmares among 67% of these patients. It is worth noting that all patients for whom improvement was reported had a comorbid diagnosis of depression, and it is reported that in the authors’ opinion, there was a significant improvement in the depressive symptoms as well as intrusion symptoms of PTSD. Furthermore, it has to be underlined that concomitant use of benzodiazepines and propranolol is reported, but in the authors’ opinion, these drugs seem to be ineffective.

In another small observational study, Falcon *et al.* [135] used clinical data from a mixed inpatient and outpatient cohort of 20 veterans with combat PTSD. They identified only 1 patient who was treated with doxepin 100 mg daily for 6-8 weeks and reported improvement in nightmares along with other intrusion symptoms.

In an uncontrolled, open-label trial [143], 18 World War II outpatient veterans with PTSD received doxepin 25-100 mg daily, and a good effect on their sleep was reported, although the lack of clarity regarding the trial duration, no use of validated scales, and concomitant use of benzodiazepines complicate the interpretation of this finding. We were unable to locate the full text of this study, and we hereby present the data as reported by Davidson *et al.* [133].

Table **16** below summarises the aforementioned findings.

### 8.5 Imipramine

Imipramine is a typical TCA in terms of mechanism of action. Relevant pharmacological properties of TCAs have been discussed elsewhere.

We identified no systematic reviews/meta-analyses or randomised controlled trials that have examined the therapeutic or adverse effects of Imipramine on sleep-related outcomes among patients with PTSD.

However, there is some evidence from observational uncontrolled studies, albeit most of them are old, underpowered, and with suboptimal reporting of outcomes. Hence, the quality of evidence presented below is overall rather poor.

In a retrospective review of clinical notes of 12 patients one year after receiving a diagnosis of PTSD (survivors of Cambodian concentration camps), Boehnlein *et al.* [142] report that 5 patients received treatment with imipramine (75-150 mg daily, N = 3 monotherapy), which resulted in the normalisation or improvement of sleep patterns and insomnia among 75% of patients, and disappearance or significant improvement of nightmares among 80% of patients. Interestingly, 3 of the 4 patients who improved were on imipramine monotherapy, 1 was on combination therapy with imipramine 150 mg and phenelzine 60 mg daily, and the one who did not improve was on a combination of imipramine 150 mg, amitriptyline 100 mg, doxepin 150 mg, and phenelzine 60 mg daily, indicating a rather treatment-refractory symptomatology. It is worth noting that all patients for whom improvement was reported had a comorbid diagnosis of depression, and it is reported that in the authors’ opinion, there was a significant improvement in the depressive symptoms as well as intrusion symptoms of PTSD. Furthermore, it has to be underlined that concomitant use of benzodiazepines and propranolol is reported, but in the authors’ opinion, these drugs seem to be ineffective.

In a small observational study, Falcon *et al.* [135] used clinical data from a mixed inpatient and outpatient cohort of 20 veterans with combat PTSD. They identified 2 patients who were treated with imipramine 150-200 mg daily for 6-8 weeks and reported improvement of nightmares along with other intrusion symptoms, with numerical values tending to favour amitriptyline over imipramine. However, they did not report on outcomes, such as insomnia and sleep quality.

Burstein [144, 145] report experience in treating patients with PTSD in a private outpatient clinic setting. The first series [144] examined the effects of imipramine treatment (200-300 mg daily) for a duration of 4 months, among 5 patients, and the author reports that all 5 participants experienced “deepening” of sleep, which was considered an improvement, within the first 5 days and for the duration of the treatment. The second case series [145] describes 15 cases (10 completers, civilian PTSD) treated with imipramine (mean dose = 260 mg, range = 50-350 mg daily) for 2-3 weeks (mean interval 17.6 days). Insomnia and nightmares were among the Impact of Event Scale (IES) items that were assessed before and after treatment, and they have been reported as the items with the greatest improvement. Scores for the item of initial/maintenance insomnia dropped from a mean of 4.8 at baseline to a mean of 2.0 at the endpoint (*p* = 0.001). Scores for the item of nightmares resembling the traumatic event dropped from a mean of 3.7 at baseline to a mean of 1.7 at the endpoint (*p <* 0.01). It is worth noting that there is no information regarding any concurrent treatment with other psychotropic medications, although the author reported that benzodiazepine treatment in this cohort had failed in the past. There was also no concrete information regarding psychiatric comorbidity. However, for the second case series, the duration of treatment was short for substantial antidepressant therapeutic effects to manifest and the author acknowledged that there was a “strong” improvement in depressive symptoms among 4 cases, moderate among 3, and mild among 3 cases.

Kinzie and Leung [146] reported their clinical experience of successful treatment of 68 refractory to treatment severely traumatised Cambodian refugee patients with PTSD and comorbid major depression with the combination imipramine (50-150 mg daily) and clonidine (0.1-0.6 mg daily). They conducted a prospective, open-label pilot study recruiting 12 patients from this cohort (11 completers) who received imipramine 50-150 mg daily as first-line for 1-2 months, which was then augmented with clonidine 0.1-0.6 mg daily if there was no adequate treatment response. 2 patients received imipramine monotherapy and 9 the combination of imipramine and clonidine. They were all followed up for 12-19 months. The authors provide data related to our study outcomes only for the 9 patients who received the combination therapy. They report that for the period of imipramine monotherapy (1-2 months), 2 of these patients reported improvement in their sleep disorder (22.2%) and 3 reported improvement in their nightmares (33.3%). After augmentation with clonidine, and over the following 12-19 months, there was an improvement in sleep among 6 patients (66.6%), resolution of the sleep disorder for 5 patients (55.5%), and improvement of nightmares among 7 patients (77.7%). It is again worth mentioning that there is no information with regards to any concurrent treatment with other psychotropic medication or psychotherapy. It is also worth noting that the study design did not allow an estimation of the effect of the natural relapsing-remitting course of depression and PTSD on the treatment outcomes. Lastly, the authors did not explore the possibility of slow responders among their study population, who would need more than 1-2 months of imipramine monotherapy to achieve the study’s therapeutic outcomes.

Table **17** below summarises the findings discussed above.

### 8.6 Tianeptine

Tianeptine is an atypical tricyclic antidepressant (TCA). It is used in European countries, but it is not FDA-approved for the treatment of depression. It does not seem to alter serotonin re-uptake or the density of serotonin receptors typically associated with antidepressant action [147]. It has anxiolytic effects, and relatively less sedative and anticholinergic effects compared to other TCAs. It has recently been shown to act as a weak agonist at the Mu opioid receptors [148]. It is unclear how it exerts its antidepressant effects; there is, however, evidence suggesting that it stimulates dopamine release in the limbic system (possibly *via* its Mu agonistic properties). It is a glutamatergic system modulator, especially in the hippocampus [149], and a modulator of the neuro-endocrine response to stress *via* the downregulation of glucocorticoid receptors [150]. Clinically, it has been associated with the improvement of both typical and somatic symptoms of depression and anxiety [147, 151].

There is very limited evidence regarding the use of tianeptine for the treatment of PTSD [21]; hence, firm conclusions cannot be drawn.

To our knowledge, there is no evidence from systematic reviews and meta-analyses relevant to our study outcomes, and we were only able to find one clinical trial including patients with PTSD that has examined outcomes relevant to this review. We have discussed this study previously, but we present it here as well for the sake of completeness.

This was a prospective, open-label, flexible-dose, randomised study with a 12-week duration comparing fluoxetine (N = 38, mean dose 27.4 mg/day; range 20-40 mg/day), moclobemide (N = 35, mean dose 565.7; range 450-900 mg/day) and tianeptine (N = 30, mean dose 41.3; range 37.5-50 mg/day) monotherapy in a sample of 103 patients that had experienced the 1990 Marmara earthquake and developed PTSD as a consequence of this [30]. Participants were enrolled in the trial between the 4^th^ and 12^th^ month after the disaster. Results suggested all of the above drugs to be efficacious in the treatment of PTSD symptoms, but there was no statistically significant difference found between them [30]. Sleep quality measures and therapeutic effects on insomnia have not been reported as study outcomes. However, insomnia has been examined as a treatment-emergent adverse effect between the groups: 2.6% (1 patient) in the fluoxetine group *vs.* 2.9% (1 patient) in the moclobemide group *vs.* 0% (0 patients) in the tianeptine group. No formal statistical analysis was performed. Furthermore, data on treatment-emergent sedation have been reported: 0% (N = 0) for the fluoxetine and the moclobemide group *vs.* 3.3% (1 patient) in the tianeptine group. No data on parasomnias have been reported.

Table **18** below presents the results of this study.

## 9 NORADRENERGIC AND SPECIFIC SEROTONERGIC ANTIDEPRESSANT (NASSA)

### 9.1 Mirtazapine

Mirtazapine, unlike other antidepressants, does not block any monoamine transporter. It however affects multiple neurotransmitter systems, with known 5-HT2A, 5-HT2C, 5-HT3, α2 adrenergic, and H1 histamine antagonism [152]. Mirtazapine is commonly used at low doses (15 mg daily) in clinical practice for its sedative and sleep-aiding properties, probably related to its H1 and 5-HT2A antagonism [124]. Mirtazapine has been found efficacious in improving PTSD symptoms [21, 44]. However, a recent network meta-analysis of randomised controlled trials found no benefit over placebo of mirtazapine in terms of its efficacy in improving sleep quality among PTSD patients, but no firm clinical conclusions can be drawn from this work due to the very limited amount of studies included [86].

Mirtazapine has been examined both as monotherapy [32, 153, 154] and add-on therapy [37] in the treatment of PTSD symptoms. Below, data from the aforementioned studies relevant to our study outcomes are presented.

#### 9.1.1 Evidence from Systematic Reviews and Meta-analyses

In a recent network meta-analysis examining the effects of antidepressants on sleep quality among patients with PTSD [86], mirtazapine was not found to be efficacious in reducing sleep disturbances or improving sleep quality, with similar acceptability to other antidepressants (sertraline and paroxetine) and placebo. However, it is worth mentioning that the certainty of evidence was low.

#### 9.1.2 Evidence from Randomised Controlled Trials - Mirtazapine Monotherapy

Davis *et al.* [154] conducted an 8-week randomised, double-blind, placebo-controlled trial followed by an 8-week open-label phase of mirtazapine monotherapy (mean dose 38.5 mg/day; range 15-45 mg/day) among 78 veterans (5 females, aged 22-56 years, N = 39 for mirtazapine, N = 39 for placebo, N = 52 entered the open phase) with predominately combat-related PTSD, recruited from outpatient settings. 59% had a comorbid depressive disorder. Panic disorder and agoraphobia were present in 20.5% and 25.6% of the participants, respectively. The Pittsburgh Sleep Quality Index (PSQI) was examined as a secondary outcome. There were no statistically significant differences at the endpoint between the mirtazapine and the placebo group in terms of improvement of PSQI scores (N = 61, ΔPSQI = -2.7 (4.3) *vs.* -3.7 (4.2), *p* = 0.414) in the double-blind phase, as well as at the end of the 8-week open-label phase compared to the end of the 8-week placebo-controlled phase (ΔPSQI = -1.2 (3.7), *p* = 0.649). It is worth noting that low-dose sedative hypnotics (trazodone, lorazepam, and temazepam) were used throughout the trial as rescue medication, which has probably limited the ability to detect significant differences in the PSQI score change. Moreover, there was a significant difference reported in the number of participants requiring sleep rescue medication between the mirtazapine and the placebo group during the double-blind phase: 11 (28.2%) participants in the placebo group *vs.* 3 participants (7.7%) in the mirtazapine group (*p* = 0.018). Insomnia was not reported as a side effect of mirtazapine in this study, but the authors reported sedation as a treatment-emergent side effect of mirtazapine among 4 patients in the mirtazapine group *vs.* 0 in the placebo group throughout the double-blinded phase, and among 3 out of the 52 patients who entered the open-label phase. Parasomnias were not examined as study outcomes, but it is reported that during the placebo-controlled phase, 3 patients experienced parasomnia (sleep disturbances/vivid dreams/nightmares) as a treatment-emergent side effect in the mirtazapine group *vs.* 2 in the placebo group, and 2 patients experienced parasomnia (nightmares/vivid dreams) during the open-label phase. No drop-outs due to parasomnias have been reported in the placebo-controlled phase, and 1 drop-out due to vivid dreams (among other side effects that this individual experienced) has been reported during the open-label phase.

Mirtazapine monotherapy (mean dose 34.1 mg/day; range 15-50 mg/day, N = 51, mean age = 59.1 years) has also been compared to sertraline monotherapy (mean dose 101.5; range 50-150 mg/day, N = 49, mean age = 60.6 years) in a 6-week randomised, open-label trial in a sample of veterans with chronic PTSD (males, 14-82% comorbid major depression or dysthymia, inpatients and outpatients) [32]. Insomnia and sleep quality measures were not studied as primary or secondary outcomes. However, data on insomnia and somnolence as treatment-emergent side effects have been reported. Somnolence has been found to be a common side effect in the mirtazapine group (15.7%, number not provided), whereas it has not been reported as a side effect in the sertraline group (number not provided). Furthermore, insomnia has been reported as a common side effect in the sertraline group (2%, number not provided), and it has not been reported as a common side effect of mirtazapine (number not provided). Lastly, it is worth emphasising that zopiclone 7.5 mg daily has been used intermittently as a rescue treatment for insomnia during the trial, and the authors have reported zopiclone to be used by 2 participants in the mirtazapine group *vs.* 5 participants in the sertraline group.

In an 8-week, randomised, double-blind, placebo-controlled clinical trial, Davidson *et al.* [155] examined the efficacy of mirtazapine monotherapy (dose range 15-45 mg daily) in the treatment of PTSD symptoms among 29 adult patients with PTSD due to a variety of trauma (4 with combat PTSD, 20 completers). Sleep measures were not examined as study outcomes, but the authors reported on treatment-emergent adverse effects. Specifically, 1 patient in the mirtazapine group developed sedation as a treatment-emergent side effect along with other adverse effects, and dropped out of the study. Sedation has not been reported as an adverse effect of placebo.

#### 9.1.3 Evidence from Randomised Controlled Trials - Mirtazapine as Add-on Treatment

Schneier *et al.* [37] conducted a 24-week, randomised, double-blind, placebo-controlled trial comparing the efficacy of mirtazapine (mean dose 32.5 mg/day; range 15-45 mg/day, N = 18) as an add-on to sertraline (mean dose 118.1; range 25-200 mg/day) with the combination of sertraline (mean dose 122.2 mg/day; range 25-200 mg/day, N = 18) and placebo in a sample of civilians with chronic PTSD (aged 18-75, 13 males). Sleep quality was one of the study’s secondary outcomes, and it was examined using the Pittsburgh Sleep Quality Index (PSQI) and the Pittsburgh Sleep Quality Index Addendum for PTSD (PSQI-A). For both measures, there were numerical advantages observed for the combined mirtazapine-sertraline treatment group, but these were not statistically significant. In terms of insomnia as a treatment-emergent side effect, there were no significant differences found between the two groups (N = 2 (15.4%) in the sertraline plus mirtazapine group *vs.* N = 2 (14.3%) in the sertraline plus placebo group, *p* = 1.00). Lastly, there were also no significant differences observed between the groups in terms of somnolence as a treatment-emergent side effect (N = 6 (46.2%) in the sertraline plus mirtazapine group *vs.* N = 5 (35.7%) in the sertraline plus placebo group, *p* = 0.70).

#### 9.1.4 Evidence from Observational Studies - Mirtazapine Monotherapy

In a small, 8-week, pilot, open-label study (N = 6, 1 male, mean age = 40.5 ± 15.8 years), mirtazapine monotherapy (mean dose 40 mg/day; range 15-45 mg/day) was efficacious in 50% of civilians with severe, chronic non-combat related PTSD with the comorbid major depressive disorder [156]. Sleep quality and insomnia-specific therapeutic outcomes have not been reported; however, the authors reported that an equal proportion of patients developed initial insomnia (N = 1) and sedation (N = 1) as a treatment-emergent side effect of mirtazapine. It is worth emphasising that in 2 patients, the concomitant use of a variety of sedative drugs was reported, including antihistamines and opiates; however, it was not reported whether these were prescribed for their sedative effect or for another therapeutic indication.

#### 9.1.5 Evidence from Observational Studies - Mirtazapine as Add-on Treatment

In a letter to the editor, Lewis [153] reported on the experience of the clinical effectiveness of mirtazapine in the treatment of nightmares and insomnia among more than 300 refugees at several neighbourhood health centres in the Chicago metropolitan area. The description of trauma in this cohort is as follows: physical and psychological torture, including the constant threat of death; being detained in prison camps or concentration camps; experiencing violence during wartime, and witnessing deaths as combatants or civilians; repeated sexual assaults by government and paramilitary officials. The author reported that mirtazapine was primarily used as an add-on therapy to an SSRI, and it was effective for approximately 75% of patients in this cohort, with marked improvement of nightmares and insomnia when mirtazapine was introduced. No further quantitative information has been provided.

Table **19** below summarises the findings discussed above.

## 10 MONOAMINE OXIDASE INHIBITORS (MAOIs)

### 10.1 Brofaromine

Brofaromine is a short-acting, selective, and reversible inhibitor of monoaminoxidase A (RIMA). This is a class of antidepressants acting on multi-neurotransmitter systems, including inhibition of the breakdown of serotonin, dopamine, and norepinephrine [157]. The benefit of selective and reversible inhibition of MAO-A is the lower risk of hypertensive crisis compared to earlier MAOIs, whilst antidepressant efficacy is comparable to earlier MAOIs and other antidepressants [158]. Despite their favourable safety profile, RIMAs are not as widely prescribed as earlier MAOIs, and existing evidence is therefore relatively limited [158].

Evidence regarding the efficacy of brofaromine for the treatment of PTSD symptoms is scarce. There are only 2 RCTs [159, 160], and both have not included insomnia and sleep quality as primary or secondary outcomes. Baker *et al.* [160] also did not report on insomnia, sleep quality, or sleepiness as adverse effects.

Katz *et al.* [159] conducted a randomised, double-blind, placebo-controlled 14-week trial. 45 out of the 64 PTSD patients recruited from an outpatient setting completed the trial (N = 22 in the brofaromine arm and N = 23 in the placebo arm). Brofaromine (50-150 mg daily) was found to be efficacious in the reduction of PTSD symptoms (DSM-III CAPS ≥ 36 at baseline). Insomnia and sleep quality were not examined as primary or secondary outcomes. However, insomnia was reported as a side effect in 34.3% of the participants receiving brofaromine *vs.* 12.1% in the placebo arm, but no formal statistical comparison was conducted. Furthermore, the authors reported that insomnia, being one of the parameters constituting the total CAPS score, was the only significant limiting factor for clinical improvement, which was quantified as a reduction of the CAPS score in this study.

In conclusion, there is essentially no evidence to support the efficacy of brofaromine for the treatment of insomnia or the improvement of sleep quality in patients with PTSD. There is only very limited evidence; on the contrary, brofaromine may be associated with increased rates of insomnia, which could be a significant limiting factor to the overall clinical improvement of PTSD symptoms.

Table **20** below summarises the aforementioned findings.

### 10.2 Moclobemide

Moclobemide is a reversible inhibitor of monoamine oxidase-A (MAOI). Due to the reversible inhibition of MAO-A, risks related to the hypertensive crisis with concurrent dietary tyramine intake are significantly lower [161]. Interestingly, treatment with MAOIs has traditionally been advocated for patients with atypical symptoms of depression, notably hypersomnia [162], thus implicating a stimulating effect. Evidence regarding the outcomes relevant to our study is presented in detail below.

To our knowledge, there are only two open-label studies that have examined the efficacy of moclobemide (300-900 mg/day) in PTSD symptoms [30, 163].

Α prospective, open-label, flexible-dose, randomised study with a 12-week duration compared fluoxetine (N = 38, mean dose 27.4 mg/day; range 20-40 mg/day), moclobemide (N = 35, mean dose 565.7; range 450-900 mg/day) and tianeptine (N = 30, mean dose 41.3; range 37.5-50 mg/day) monotherapy in a sample of 103 patients that had experienced the 1990 Marmara earthquake and developed PTSD as a consequence of this [30]. Participants were enrolled in the trial between the 4^th^ and 12^th^ month after the disaster. Results suggested that all of the above drugs were efficacious in the treatment of PTSD symptoms, but there was no statistically significant difference between them [30]. Sleep quality indices and therapeutic effects on insomnia have not been reported as study outcomes. However, insomnia has been examined as a treatment-emergent adverse effect between the groups: 2.6% (1 patient) in the fluoxetine group *vs.* 2.9% (1 patient) in the moclobemide group *vs.* 0% (0 patients) in the tianeptine group. Also, no formal statistical analysis has been performed. Furthermore, data on treatment-emergent sedation have been reported: 0% (N = 0) for the fluoxetine and the moclobemide group *vs.* 3.3% (1 patient) in the tianeptine group. No data on parasomnias have been reported.

Neal *et al.* [163] conducted a prospective, open-label, 12-week pilot uncontrolled study that examined the effectiveness of moclobemide monotherapy (300-600 mg/day) in a sample of 20 civilians, victims, and veterans with PTSD (N = 18 completers). The authors reported moclobemide monotherapy to be effective in reducing PTSD symptoms. In terms of outcomes relevant to our study, they reported that at a dosage of 300-600 mg/day, moclobemide was efficacious in inducing a reduction in difficulty sleeping, measured as a part of the Computerised Clinician-administered PTSD Scale 1-Revised (CC-R-1, pre-treatment score = 2.5 ± 0.86 *vs.* post-treatment score = 1.33 ± 0.86, effect size = 1.14 (0.55-1.71), *p <* 0.001). Furthermore, they reported a significant reduction in nightmares (CC-R-1 pre-treatment score = 2.06 ± 1.21 *vs.* post-treatment score = 1.22 ± 1.06, effect size = 1.56 (0.79-2.35), *p <* 0.014). It is worth mentioning that participants were allowed concurrent use of benzodiazepines, but the dose was not changed for a period of 4 weeks prior to the trial initiation and up to the end of the trial. No significant differences were found in total CC-R-1 between benzodiazepine users (N = 4) *vs.* no benzodiazepine use (N = 14, df = 3.48, F = 0.25, *p* = 0.86). However, formal statistical analysis examining the differences in sleep outcomes between these 2 subgroups was not performed. Insomnia and somnolence have not been reported as treatment-emerging side effects in this trial.

Moclobemide was found to be well tolerated among patients with PTSD included in the studies above [30, 163].

Table **21** presents a summary of the results discussed above.

### 10.3 Phenelzine

Phenelzine is a first-generation, potent, non-selective, and irreversible inhibitor of monoaminoxidase (MAOI) [164]. Due to its non-selective and irreversible inhibition, there are significant safety concerns, including hypertensive crisis when there is concomitant dietary tyramine intake [165]. It has traditionally been used for the treatment of atypical depression or depression refractory to treatment with safer agents, and it is FDA-approved for the treatment of treatment-resistant depression, panic disorder, and social anxiety disorder [164, 165].

Phenelzine is not used clinically as a first-line pharmacotherapy for PTSD. Interestingly, however, there is evidence to suggest that it may be superior in terms of efficacy for the treatment of PTSD symptoms compared to the first-line and FDA-approved treatment options, such as sertraline [44, 45].

Phenelzine has been shown to reduce sleep disturbances among patients with depression, potentially due to its potent REM-suppressing effect [92], and it may even abolish dream activity at doses above 60 mg daily [166]. REM sleep fragmentation and autonomic imbalance are associated with the development and persistence of PTSD symptoms [93]. Although there is no robust evidence to support a hypothesis that the potential superiority of phenelzine in terms of efficacy is attributable to its potent REM suppression effects, it is an interesting hypothesis.

Below, we have summarised existing evidence on the therapeutic and adverse effects of phenelzine on sleep disturbances among patients with PTSD. Most studies are rather old; there is no evidence from systematic reviews/meta-analyses, and there is only 1 RCT reporting relevant results. Also, results from observations studies and case series are sub-optimally reported. The quality of evidence is overall rather low.

#### 10.3.1 Results from Randomised Controlled Trials

In a 12-week double-blind, randomised cross-over clinical trial, Shestatzky *et al.* [167] randomised 13 patients to receive either phenelzine (45-75 mg daily) or placebo for 5 weeks, who then crossed over to the alternate treatment for another 5 weeks, after a 2-week washout period of placebo treatment. Only 10 patients (26-50 years old) completed at least 4 weeks of treatment with both interventions, all of whom had a diagnosis of PTSD (DSM-III) with a mixture of combat and civilian-related index trauma (4 combat). 2 patients had a comorbid diagnosis of generalised anxiety disorder and 2 had a comorbid diagnosis of major depression. Concurrent treatment with any psychotropic medication was not allowed and all patients received supportive psychotherapy only for the duration of the trial. Sleep disturbances and nightmares (dreams of the event) were examined using the Post-Traumatic Stress Disorder Scale (PTSD-Scale), consisting of the 12 items constituting the DSM-III criteria for PTSD. Interestingly, there were no significant differences observed between phenelzine and placebo for sleep disturbances (mean improvement for phenelzine = 0.8 *vs.* 0.5 for placebo at 4 weeks and overall improvement of 1.1 at week 4 of phase 2, no formal statistical analysis reported). Similarly, there were no significant differences observed between the 2 groups for nightmares of the event (mean improvement for phenelzine = 0.6 *vs.* 0.7 for placebo at 4 weeks and overall improvement of 1.1 at week 4 of phase 2, no formal statistical analysis reported). The authors report that an extremely high number of participants would be required to disprove the null hypothesis (2000 participants for a difference at *p <* 0.05 with a power of 0.8 on the PTSD scale). No data on sleep-related treatment-emergent adverse effects have been reported in this study.

#### 10.3.2 Results from Observational Studies and Case Series/Reports

A number of observational studies, case series, and case reports have examined sleep outcomes among patients with PTSD.

A pilot, 6-week open prospective, uncontrolled trial of phenelzine monotherapy [168] (mean dose = 45 mg, range = 45-60 mg daily) among 11 patients (10 completed 4 weeks) with chronic PTSD (concomitant use of other psychotropics was not allowed) demonstrated an average 41% improvement in scores for “sleep disturbance” and 42% improvement in scores for “recurrent dreams” after 4 weeks of treatment. Interestingly though, the same study identified intensification of pre-existing sleep disturbance among the most troublesome treatment-emergent side effects, occurring in 4 patients (36.7%). One patient (9.1%) also reported sedation.

Another prospective, uncontrolled open-label trial of at least 4 weeks duration [169] examined the effects of phenelzine monotherapy (mean dose = 60 mg, range = 30-90 mg daily) on PTSD symptoms among 25 Israeli combat veterans with combat PTSD (22 completers of at least 4 weeks of treatment, 20-44 years old, 4 having comorbid panic disorder, 1 having comorbid major depression, 6 having comorbid dysthymia, and 2 having comorbid generalised anxiety disorder). The authors reported sleep disturbance as the most severe symptom and the one that was most consistently improved (average score change of 36.4%, *p* = 0.01). There was also a moderate improvement reported in traumatic events (average score improvement = 18.2%, *p* = 0.05). Insomnia was also examined as an item of the HAM-D scale. 35.7% improvement in average initial insomnia scores and 27.3% improvement in average maintenance insomnia scores were reported, but no improvement in early morning awakening has been reported. Insomnia was not reported as a treatment-emergent adverse effect, but drowsiness was reported among 3 participants, who dropped out due to this and other adverse effects. It is worth noting that whether concurrent use of other psychotropic medications (as required treatment for sleep) was allowed or not has also not been reported.

In another open-label, uncontrolled 8-week trial of phenelzine monotherapy [170] (dose = 1 mg/kg), it was reported that 60% of the 10 included outpatient Vietnam veterans with combat PTSD experienced significant improvement in intrusion symptoms, including nightmares (Zung scale), and that improvement was rapid and maintained at 8 weeks.

In a series of 5 cases, Hogben *et al.* [171] reported their experience of treating combat PTSD with phenelzine (45-75 mg daily), either as monotherapy or as add-on treatment to previously prescribed medication (benzodiazepines, TCAs, chlorpromazine among others) that failed to control the symptoms. They reported a remission of nightmares of the traumatic event among 100% of the patients (all 5), which was maintained in follow-up reviews (up to 18 months).

A series of 3 cases [166] and another 2 case reports [172, 173] were in agreement with the findings presented above, and reported either reduced frequency and intensity or disappearance of traumatic nightmares, along with improvement in other intrusion symptoms, using phenelzine 60-105 mg daily to treat veterans being refractory to treatment of combat PTSD. It is, however, worth noting that no information has been provided with regards to concurrent treatment with other psychotropic agents for all the aforementioned cases.

Table **22** below summarises the aforementioned findings.

## 11 OTHER ANTIDEPRESSANTS

### 11.1 Bupropion

Bupropion belongs to the class of norepinephrine-dopamine reuptake inhibitors (NARDs). It is a weak inhibitor of the dopamine transporter (DAT) and the norepinephrine transporter (NET), and it also blocks nicotinic acetylcholine receptors [174].

The efficacy of bupropion on a variety of outcomes among patients with PTSD has been examined in two RCTs [35, 36], one open-label trial [175], and two case reports [176, 177].

Becker *et al.* [35] conducted an 8-week randomised, double-blind, placebo-controlled variable dose trial to examine the efficacy of bupropion (100-300 mg daily) on PTSD symptom reduction, sleep quality, and depressive symptoms, among outpatients (34-62 years of age) with PTSD (79% veterans, 79% men) who were deemed to be stable in their mental state prior to study enrolment. 28 patients were included in the intent-to-treat analysis (N = 18 for bupropion, N = 10 for placebo). Only 23 participants completed week 8 of the trial. In the bupropion arm, 12 patients were also on another antidepressant and 4 were on an atypical antipsychotic, the dose of which was kept stable during the trial. On the placebo arm, 6 patients were on another antidepressant at the time of the study. The Pittsburgh Sleep Quality Index (PSQI) was used to assess sleep quality and it was one of the study’s primary outcomes. Although there was no significant group differential effect in favour of bupropion *versus* placebo for PTSD symptoms reduction, depressive symptoms and overall sleep quality (total PSQI) among 8-week completers (N = 23), there was a significant group effect for a single subcategory of the PSQI, namely daytime dysfunction (intent-to-treat analysis, F (1,23) = 4.38; *p* < 0.05). Examination of effect sizes compared to baseline demonstrated a moderate effect of bupropion on reduced sleep latency and large effects on improving global sleep quality and daytime dysfunction. It is important that the limitations of this study are highlighted, and these include: i) the small sample size; ii) slow titration of medication (maximum dose of 300 mg daily not prescribed until week 4 and total duration of the trial being 8 weeks); and iii) large proportion of patients on other psychotropic medication; hence, the efficacy of bupropion as monotherapy could not be reliably assessed.

In another small, 12-week randomised, double-blind, placebo-controlled trial, Hertzberg *et al.* [36] enrolled 15 veterans with chronic combat PTSD (47-58 years of age) and examined the efficacy of bupropion (150-300 mg daily) as a smoking cessation intervention as their primary outcome. Among other outcomes examining symptomatic improvement, none of which yielded statistically significant results, sleep quality was examined using the Pittsburgh Sleep Quality Index (PSQI). 7 patients in the bupropion group (300 mg daily) completed the 12 weeks, and no significant improvement in their overall PSQI scores was identified. Data on the subcategories of the PSQI have not been provided. Apart from the very small number of participants, concurrent prescription of other psychotropics in 9 out of 10 patients randomised to the bupropion group was a limitation that affected this trial as well.

In an open-label 6-week trial, Cañive *et al.* [175] enrolled 17 male veterans with chronic PTSD (46-71 years of age), who were either antidepressant-naive, had complained of side effects, or had failed previous adequate trials of other antidepressants. They were prescribed bupropion 200-400 mg/day, and outcomes included validated measures of symptoms intensity and clinical global impression (CGI-I). Insomnia and sleep quality were not examined as study outcomes. However, loss of sleep has been reported as one of the reasons for bupropion discontinuation in three patients, but further details regarding the nature or the degree of this side effect have not been provided.

Insomnia or deterioration of sleep quality have not been reported as side effects of bupropion in the 2 RCTs [35, 36], but they have been reported in the Cañive *et al.*’s open trial [175] without being quantified.

In summary, there is no evidence to support the use of bupropion for the treatment of insomnia in PTSD. Instead, there is very limited, poor-quality evidence to support that it can be a side effect of the drug among patients with PTSD. Further studies will be required to inform clinical practice (Table **23**).

### 11.2 Vilazodone

Vilazodone combines the actions of a partial agonist at the 5-HT21A receptor with serotonin reuptake inhibition (SPARI, Serotonin Partial Agonist Reuptake Inhibitor) [178]. In theory, the immediate 5-HT21A partial agonism and the SERT inhibition are synergistic, resulting in a faster and more robust antidepressant effect compared to SERT inhibition alone, with better tolerability.

In clinical practice, vilazodone is not used commonly, although it is approved for the treatment of major depression in the USA [179], and it is supported by evidence for its safety and tolerability [178].

To our knowledge, only one clinical trial has examined the efficacy and safety of vilazodone for the treatment of PTSD [180, 181].

In the unpublished version of the study [180], the use of the PSQI for the assessment of sleep quality has been reported, but there are no available results. In the published version [181], PSQI has not been reported among the outcome measures. We were unable to locate the PSQI data of this study elsewhere. However, there are data on sleep quality evaluated using other measures and data on sleep-related adverse effects; hence, we will discuss this trial.

This study [181] was a randomised, double-blind, placebo-controlled, 12-week trial examining the efficacy and safety of vilazodone monotherapy (N = 29, 10-40 mg daily, slow titration, max 40 mg at week 3, 25 completers) *vs.* placebo (N = 30, 22 completers) for the treatment of PTSD symptoms among 59 male outpatient veterans with chronic combat-related PTSD, comorbid with at least mild depression (18-55 years old). Concomitant treatment with zolpidem as required for sleep was permitted during the trial. Sleep symptoms were assessed using items 2 and 13 of the CAPS (Clinician-Administered PTSD Scale, DSM-IV), item 13 of the PSS-SR (PTSD Symptom Scale-Self-Report, DSM-IV), and item 16 of the BDI-II (Beck Depression Inventory-II). No significant differences between the vilazodone and the placebo group were identified for all the above sleep measures (*p >* 0.1). However, each group showed significant reductions in CAPS-item13, PSS-SR-item 13, and BDI-II item 16 from baseline to endpoint (week 12). In terms of safety and tolerability sleep outcomes, the authors reported that sleep-related treatment emergent adverse effects were among the most commonly reported, but they did not provide further information either in the published [181] or the unpublished version of the trial [180].

Table **24** below summarises the aforementioned findings.

### 11.3 Vortioxetine

There is a limited number of studies examining the use of vortioxetine for the treatment of PSTD [182-184].

None of the available studies report on outcomes relevant to our review. We will, therefore, not discuss this drug further.

## 12 META-ANALYSIS RESULTS

We completed meta-analytic evaluations for specific efficacy and safety outcomes wherever data from controlled studies were available and it was technically possible to do so.

### 12.1 Sleep-related Efficacy Outcomes

#### 12.1.1 Sleep Quality

**Fig. (1):**
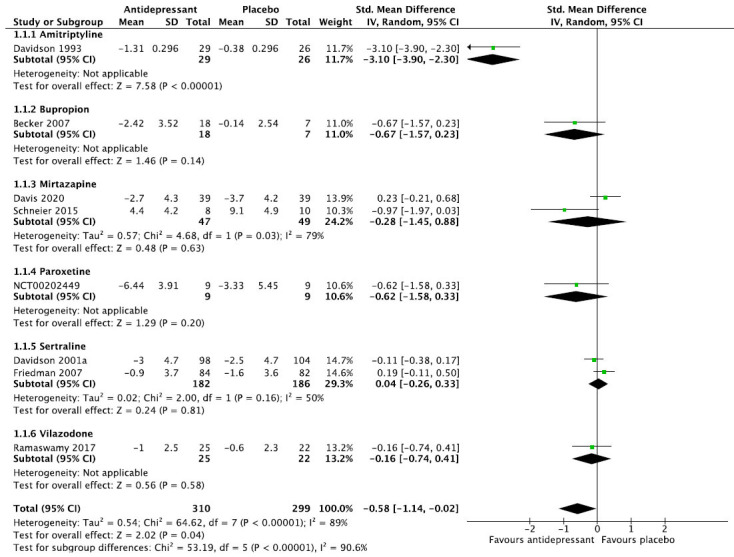
Standardised mean difference for efficacy outcome ‘sleep quality’ of all antidepressants *versus* placebo. **Abbreviations:** N = Number, Std. = Standardised, IV = Inverse variance, CI = Confidence interval.

Amitriptyline was the only antidepressant showing a benefit over placebo in terms of improving sleep quality, but the result was based on only one, rather old and small study [134] (Fig. **1**, SMD = -3.10, 95% CI -3.90 to -2.30, 1RCT, N = 55). The remaining antidepressants, namely bupropion, mirtazapine, paroxetine, sertraline, and vilazodone, did not differ significantly from the placebo, but few data were available per drug (Fig. **1**). Thus, no definitive interpretation has been made.

#### 12.1.2 Distressing Dreams

**Fig. (2):**
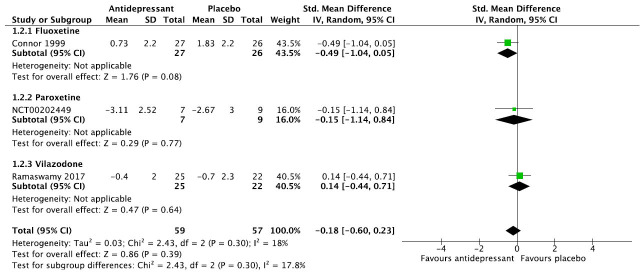
Standardised mean difference for efficacy outcome ‘distressing dreams’ of all antidepressants *versus* placebo. **Abbreviations:** N = Number, Std. = Standardised, IV = Inverse variance, CI = Confidence interval.

Only 3 studies (N = 116 in total) reporting on the potential therapeutic effect of antidepressants on distressing dreams (otherwise reported as nightmares resembling trauma) were eligible for meta-analytic evaluation [58, 87, 181]. There was a trend in favour of fluoxetine over placebo (Fig. **2**, SMD = -0.49, 95% CI -1.04 to 0.05, 1RCT, N = 53), whereas paroxetine and vilazodone did not show any favourable effect. Again, only one small RCT was available per drug, and thus, the results have been inconclusive.

### 12.2 Sleep-related Treatment-emergent Adverse Effects

#### 12.2.1 Insomnia

Fig. (**3**) presents the risk ratios of antidepressants *versus* placebo for insomnia as an adverse effect. There was a trend in favour of amitriptyline (RR = 0.17, 95% CI 0.02 to 1.33, 1 RCT, N = 46), but it was based on only one old and small study [134]. All other antidepressants did not differ significantly from the placebo. Especially for SSRIs, this result was rather robust since data from 17 RCTs and 3172 participants were available (Fig. **4**).

#### 12.2.2 Somnolence

Fig. (**5**) presents the risk ratios of antidepressants *versus* placebo for somnolence/drowsiness as an adverse effect. There was a trend that paroxetine could cause more somnolence than placebo (RR = 1.96, 95% CI 0.89 to 4.29, 5 RCTs, N = 1240), but heterogeneity was high (I^2^ = 80%, *p <* 0.001); thus, caution is warranted in terms of results interpretation. Heterogeneity was caused by the Simon *et al.*’s study [39], which found high rates of drowsiness in both antidepressant (66.7%) and placebo (78.6%) groups that differed from rates as presented in all other studies (antidepressants 4% to 26%; placebo 4% to 16%). When this study was excluded, paroxetine was clearly worse than placebo (RR = 2.82, 95% CI = 1.70 to 4.68) and heterogeneity was importantly reduced (I^2^ = 29%, *p* = 0.24; Fig. **6**). All other antidepressants did not differ significantly from the placebo. As for the subgroup of SSRIs, this class of antidepressants seems to cause more somnolence than placebo (Fig. **7**), but this result might be an effect of paroxetine, rather than a class effect.

#### 12.2.3 Nightmares/Vivid Dreams

Fig. (**8**) presents the risk ratios of antidepressants *versus* placebo for nightmares/vivid dreams as a treatment-emergent adverse effect. Only 6 RCTs reported data on three antidepressants, namely mirtazapine, paroxetine, and sertraline, and no difference was identified either for individual drugs or meta-analytically.

## 13 DISCUSSION

In this overview of reviews, we have attempted to examine the therapeutic and adverse effects of antidepressants on sleep disturbances in patients with PTSD.

Antidepressants are safe and effective therapeutic options for the treatment of symptoms of PTSD [21], and the clinical reality is that limited access to psychotherapeutic interventions often results in long-term treatment with antidepressants before patients commence evidence-based psychotherapy. Furthermore, psychotherapy is associated with significant drop-out rates [185], and pharmacotherapy is often necessary in the real world of clinical practice. Due to the recent advances in the understanding of the biological mechanisms involved in PTSD, both from a neuroscience [186, 187] and from a clinical perspective [20], there is increasing acceptance of the school of thought advocating that pharmacotherapy is of importance in the treatment of PTSD [21].

Sleep disturbances are present in 70-90% of patients with PTSD [188], which are a risk factor for the development of PTSD post-trauma [189], associated with chronicity of PTSD symptoms, but also related to poorer response to treatment [190].

In clinical practice, benzodiazepines and z-drugs are commonly used for the treatment of insomnia, either as an isolated symptom or as a symptom of a psychiatric disorder. However, well-recognised concerns regarding their misuse and dependency potential have prompted further research into the off-label use of other agents, including antidepressants [19]. This is particularly relevant for patients with PTSD, as it is known that comorbidity with substance misuse is high, and perhaps represents an attempt at self-medication [20, 21].

However, data for non-benzodiazepine drugs are scarce, and no firm conclusions can be drawn from the existing evidence in terms of their safety and efficacy for the management of insomnia in adults [18, 19]. Even α-adrenergic blocker treatment (prazosin), which is known to improve distressing dreams (nightmares resembling trauma), was not found to improve sleep quality in patients with PTSD in a recent meta-analysis [191].

Therefore, the question as to whether the commonest and most easily accessible therapeutic intervention for PTSD (*i.e*., antidepressant drugs) has significant therapeutic and/or adverse effects on sleep disturbances among patients with PTSD is clinically very relevant and an important one to answer.

Our findings, thus, indicate that there is not enough evidence to allow a reliable and conclusive answer to the aforementioned clinical question, and highlight a number of important general observations with regards to the available published literature:

1. There is essentially no conclusive evidence from systematic reviews and meta-analyses exploring the therapeutic and adverse effects of antidepressants in sleep-related PTSD symptoms.
2. The vast majority of randomised controlled trials have not examined sleep disturbances as primary or secondary outcomes, and do not report on the therapeutic effects of antidepressants on sleep disturbances.
3. Overall, the majority of the available studies clearly supporting the use of antidepressants for the treatment of sleep-related PTSD symptoms are rather old, observational, poorly reported, and also often poorly designed and underpowered.
4. Validated measures of sleep quality and other sleep parameters and sleep-related symptoms are not often used, even among studies that report on the therapeutic effects of antidepressants on sleep disturbances. This is especially true for the studies that report a clear therapeutic benefit.
5. Even when validated measures are used, these are not focused on PTSD-specific sleep-related symptoms, and outcomes are loosely defined. For example, a very limited number of studies use PTSD-specific measures, such as the PSQI-A [192], or differentiate between nightmares resembling the index trauma and other types of nightmares and vivid dreams.
6. Furthermore, in most studies reporting improvement of sleep disturbances with antidepressants, the majority of the study populations had a comorbid diagnosis of major depression and often other psychiatric comorbidities, and the use of non-specific measures and/or lack of subgroup analyses did not allow for exploration of the differential effects on PTSD-specific sleep disturbances.
7. Although there is a greater number of studies reporting on safety outcomes and sleep-related adverse effects compared to the number of studies reporting on therapeutic effects, terminology and definitions of outcomes are again inconsistent, which leads to discrepancies between reported outcomes. For example, terms, like sedation, somnolence, hypersomnia, sleepiness, and drowsiness are used among studies, and definitions are not reported in detail. In the vast majority of studies, the way of reporting indicates that these terms are used to describe the same symptom from a clinical perspective.
8. In general, most studies have allowed the concomitant use of as-required treatment with benzodiazepines or other sedatives/hypnotics for the duration of the trial, and relevant information has not been provided. Although the general observation is that studies designed to examine sleep-related outcomes tended to limit this to the first week or 2 weeks, or completely avoided concurrent rescue treatment for insomnia, the number of these studies is limited and data are insufficient to allow for a formal exploration of this in a subgroup analysis of a meta-analytic evaluation.
9. Similarly, there is insufficient data to allow for a meaningful comparison between the effects of antidepressants as monotherapy *vs.* as add-on treatment to other pharmacological and non-pharmacological interventions.

Overall, we found that antidepressants with the greater amount of evidence (albeit very limited and of low overall quality) suggesting that they might represent an alternative treatment option for sleep disturbances among patients with PTSD were amitriptyline (Fig. **1**, Table **13**), paroxetine (Table **6**, 1 pooled analysis of RCTs [84, 85]), sertraline (Table **7**, 1 network meta-analysis [86], 1 pooled analysis of RCTs [96]) and nefazodone (Table **10**, 1 pooled analysis of observational trials [113], but no corroborating RCT or meta-analytic evidence [33, 86]).

It is worth noting that most studies have included patients with chronic PTSD, and in many cases, mainly male veterans with combat PTSD have been recruited. It is reported that females respond to pharmacotherapy better than males, and pharmacotherapy is generally less effective in chronic illness [98]. Therefore, the findings of studies including only (or a substantial proportion of) male veterans may not apply to patients with PTSD due to other types of trauma, milder symptoms, or with a shorter duration of illness, and might explain our observation that antidepressants, in general, are not greatly effective in treating sleep disturbances among patients with PTSD. It is also worth noting that none of these studies report on subgroup analyses based on gender, trauma type, chronicity, *etc*., which limits our ability to explore these associations in our meta-analytic evaluation.

Our general observation is that treatment outcomes tended to be worse among older patients compared to younger patients, but a very limited number of studies reported specifically on this [126]. The known association between poorer response to treatment and chronicity of symptoms [190] might be underpinning this association. Due to the lack of available data, we were technically unable to explore this further and quantify it with meta-analytic evaluations. Better reporting of potential age-related differences in future studies would perhaps allow for further exploration of this association.

Interestingly, however, a significant number of the studies demonstrating a beneficial effect of antidepressants on sleep-related outcomes included male veterans with chronic PTSD. Some of these studies report that sleep symptoms were among the first to show improvement and this played a significant role in the overall improvement of PTSD symptoms and quality of life. This is not surprising, as there is a known association between sleep and quality of life [193]. However, these studies are rather old, methodological, and reporting problems were evident in many, and all of them were underpowered. Therefore, safe and generalisable clinical conclusions cannot be drawn based on these results.

Nevertheless, we did find and include 1 trial involving children and adolescent population [102], but the authors did not report on the therapeutic effects of the antidepressant examined (sertraline) on sleep outcomes, and the sleep-related adverse effect profile was not markedly different in this population compared to adult populations. Further studies on children and adolescent populations reporting on sleep-related outcomes are required as the current evidence base is negligible.

We ought to mention here that most studies have examined the short-term effects of treatment with very few reporting follow-up results in the medium and the long term. Older observational studies with the limitations discussed above have reported maintenance of therapeutic effect for amitriptyline (Table **13**) and nefazodone (Table **10**) from 6.5 months to 4 years after treatment initiation.

It is plausible to hypothesise that sedative antidepressants would be a superior treatment option in terms of sleep-related symptoms compared to SSRIs, for example, which are known as a class to impair sleep continuity and increase awakenings as well as decrease sleep efficiency, while most TCAs are known to do the opposite [194]. This line of thought is also in keeping with the common clinical practice of prescribing, driven by common sense and evidence extrapolated from studies examining diverse patient populations in terms of diagnosis [124].

Our findings suggest that studies using sedative antidepressants were overall more likely to report on sleep-related therapeutic effects and demonstrate a beneficial effect. It has to be underlined, however, that the quality of this evidence is overall very low, and overall lower compared to the evidence that indicates that SSRIs, particularly paroxetine and sertraline, may have beneficial effects on sleep-related symptoms.

Our meta-analytic evaluation indicates that the only drug for which a significant benefit over placebo was identified was amitriptyline with a large effect size (Fig. **1**, N = 55, SMD = -3.10, 95% CI -3.90 to -2.30). However, the result was based on only one, rather old and small study [134]. Older trials might show exaggerated results due to sub-optimal randomisation or blinding quality and publication bias [195]. On the other hand, there is a hypothesis that modern, industry-sponsored trials may lead to increased placebo response rates, and therefore, lower effect sizes [19].

Amitriptyline is a commonly prescribed non-benzodiazepine drug for the treatment of insomnia with reported benefits in a variety of disorders [124]. TCAs, and especially amitriptyline and imipramine, have been found to increase the length of the sleep cycle and improve the maintenance of insomnia in patients with PTSD [79], which may explain our finding. Unfortunately, the lack of data availability did not allow for a more detailed evaluation of parameters that could potentially result in the improvement of sleep quality. We also did not find any controlled study examining the effects of imipramine on relevant outcomes among patients with PTSD.

Based on the above, and with the obvious limitation of the scarcity of available data, our findings seem to support the common practice of the use of amitriptyline as a treatment of sleep disturbances, and indicate that it might be a useful treatment option for the clinician when sleep quality is a major problem.

Interestingly, no significant benefit over placebo has been identified for mirtazapine, another commonly prescribed sedative antidepressant, and our findings do not support the current practice of prescribing, although the evidence is scarce, and further original research is needed to allow for firm conclusions to be drawn.

Distressing dreams (also reported as nightmares resembling trauma) are among the most common (50-70%) and disturbing symptoms of PTSD [190]. Despite their significance, most studies do not report on this symptom specifically. As nightmares are primarily a REM sleep phenomenon [196], it would be plausible to hypothesise that potent REM suppressors, such as phenelzine, would be a good treatment option, perhaps superior to SSRIs, for example, which are known to suppress REM sleep, but to a lesser extent, and relevant clinical outcomes are reported to be relatively less robust compared to phenelzine [194]. Our findings suggest that the very limited existing evidence does not indicate a class effect, and antidepressants overall do not seem to have a favourable effect over placebo. However, it is worth mentioning that there was a trend observed in favour of fluoxetine over placebo (Fig. **2**, SMD = -0.49, 95% CI -1.04 to 0.05, 1 RCT, N = 53). We could only find 1 old RCT with data on the effects of phenelzine on distressing dreams [167], which reports no difference *vs.* placebo, but these data were not usable for the meta-analysis as no standard deviation values were reported. However, all observational studies using phenelzine have reported positive results (Table **22**). More and better designed clinical trials are required in order to explore the potential therapeutic effects of antidepressants on distressing dreams, and a comparison between phenelzine and SSRIs would be interesting.

It is perhaps worth mentioning that a deterioration of distressing dreams is reported with duloxetine [107], but this evidence comes from a case report of a patient with significant psychiatric multi-comorbidity (bipolar disorder, major depressive episode); psychotropic polypharmacy was another important limitation. Therefore, these results have to be considered carefully and the aforementioned caveats limit the generalisability of the reported findings.

**Fig. (3):**
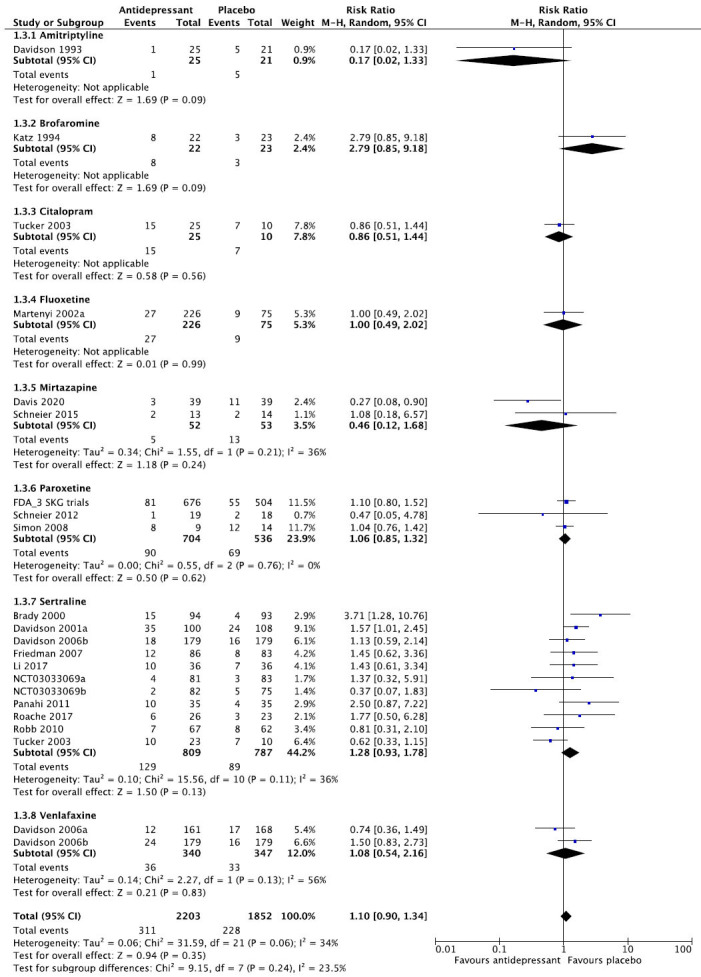
Response ratio for safety outcome ‘insomnia’ of all antidepressants *versus* placebo. **Abbreviations:** N = Number, M-H = Maentel-Haenszel, CI = Confidence interval.

**Fig. (4):**
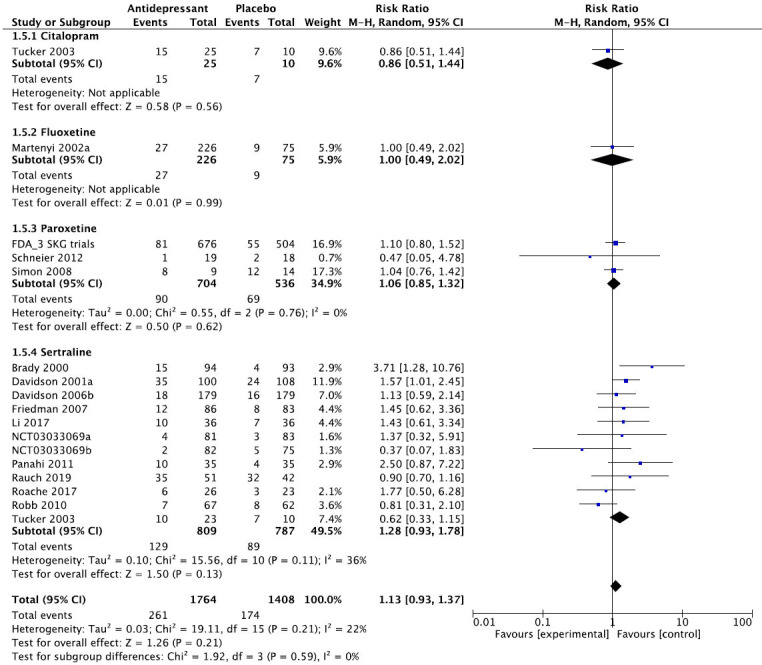
Response ratio for safety outcome ‘insomnia’ of all SSRIs *versus* placebo. **Abbreviations:** SSRIs = Selective serotonin reuptake inhibitors, N = Number, M-H = Maentel-Haenszel, CI = Confidence interval.

Our meta-analytic evaluation shows no significant effects of the comparisons between amitriptyline, venlafaxine, mirtazapine, and commonly prescribed SSRIs *vs.* placebo in terms of insomnia (Figs. **3** and **4**) and vivid dreams or nightmares (Fig. **8**). For the majority of individual comparisons, our findings were based on a limited number of patients from a small number of trials, or in some cases, even a single study, making most of the results inconclusive, with the exception of sertraline and paroxetine, for which larger numbers were available and their results have been considered more robust (Figs. **4** and **8**).

We identified a trend indicating less treatment-emergent insomnia for amitriptyline compared to placebo (Fig. **3**). The number of patients was low (1 RCT, N = 46), which possibly explains the fact that this finding did not reach statistical significance. This trend is not surprising and corroborates our finding that amitriptyline might improve sleep quality.

Less data are available for older and less commonly prescribed antidepressants. However, we identified no notable adverse effects on the sleep outcomes examined, with possibly the exception of brofaromine (Table **20**). There is limited evidence from a randomised controlled trial [159] that brofaromine may be associated with increased rates of insomnia, which were presented in this study as a significant limiting factor to the overall clinical improvement of PTSD symptoms.

**Fig. (5):**
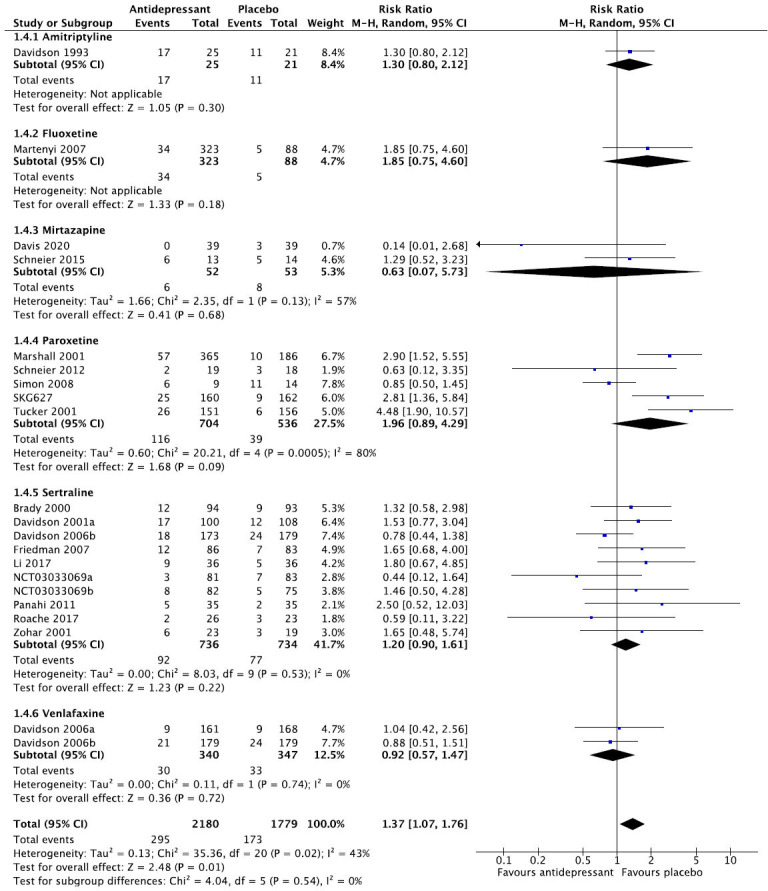
Response ratio for safety outcome ‘somnolence’ of all antidepressants *versus* placebo. **Abbreviations:** N = Number, M-H = Maentel-Haenszel, CI = Confidence interval.

**Fig. (6):**
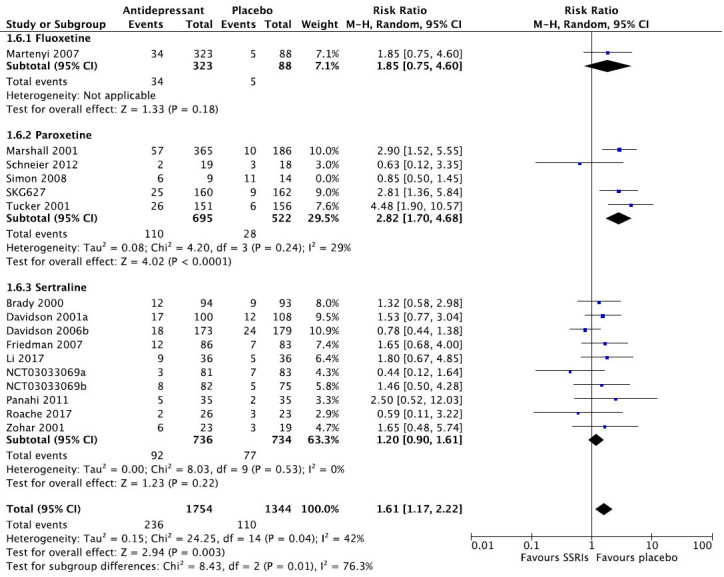
Response ratio for safety outcome ‘somnolence’ of all SSRIs *versus* placebo without the Simon’s (2008) study. **Abbreviations:** SSRIs = Selective serotonin reuptake inhibitors, N = Number, M-H = Maentel-Haenszel, CI = Confidence interval.

**Fig. (7):**
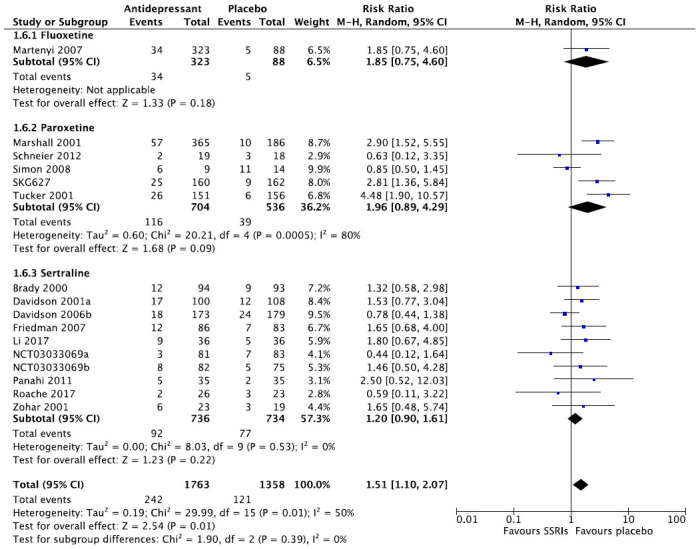
Response ratio for safety outcome ‘somnolence’ of all SSRIs *versus* placebo. **Abbreviations:** SSRIs = Selective serotonin reuptake inhibitors, N = Number, M-H = Maentel-Haenszel, CI = Confidence interval.

A noteworthy finding is that somnolence (drowsiness, hypersomnia, sleepiness, and sedation) was significantly more commonly reported for antidepressants overall (Fig. **5**, 19 RCTs, N = 3959, RR = 1.37, 95% CI 1.07 to 1.76), and for SSRIs when examined as a subgroup (Fig. **7**, 15 RCTs, N = 3121, RR = 1.51, 95% CI 1.10 to 2.07). Nevertheless, it seems that this finding is driven by paroxetine and it is unclear whether a class effect exists. Paroxetine was shown to cause more somnolence than placebo although not significantly (5 RCTs, N = 1240, RR = 1.96, 95% CI 0.89 to 4.29,), but heterogeneity was high (I^2^ = 80%, *p <* 0.001), and thus, caution is warranted in terms of results interpretation. As discussed previously, heterogeneity was caused by Simon *et al.*’s study [39], which found high rates of drowsiness in both antidepressant (66.7%) and placebo (78.6%) groups that differed from rates as presented in all other studies (antidepressants 4% to 26%; placebo 4% to 16%). When this study was excluded, paroxetine was clearly worse than placebo (RR = 2.82, 95% CI = 1.70 to 4.68) and heterogeneity was importantly reduced (I^2^ = 29%, *p* = 0.24; Fig. **6**). It is reported in the literature that sedation and hypersomnia are more commonly observed with paroxetine compared to other SSRIs [197, 198], and that somnolence is a commonly reported adverse effect, especially in the short-term, slightly more commonly reported than insomnia [199]. This is perhaps due to the fact that paroxetine is the most anticholinergic of all SSRIs [200].

## 14 STUDY LIMITATIONS

Our study has several limitations. First and foremost, data on sleep-related efficacy and safety outcomes are scarce for antidepressants, which significantly impacts the value of the meta-analytic evaluations presented, and in general, limits the generalisability and clinical utility of the findings of our study.

Secondly, we ought to mention some methodological limitations. Being an overview of reviews, this study did not follow a systematic review process in terms of search and reporting methods, including formal assessment of the quality of evidence and publication bias. However, the search methods employed were robust, and it is unlikely that significant data were missed, especially data that would be usable for a meta-analytic evaluation. Moreover, we limited our scope to antidepressant drugs, and did not examine other pharmacological treatments, such as a-adrenergic blockers, benzodiazepines, z-drugs, antiepileptics, antihistamines, antipsychotics, b-blockers, opioid antagonists and buspirone, to mention a few, some of which have significant supporting evidence for the treatment of sleep-disorders in PTSD.

## CONCLUSION

There is a limited number of studies examining the therapeutic effects of antidepressants on sleep outcomes among patients with PTSD. There is no conclusive meta-analytic evidence; most randomised controlled trials have not examined sleep symptoms as primary or secondary outcomes, and most studies clearly reporting a therapeutic benefit are rather old, largely observational, underpowered, and often sub-optimally designed and reported.

Antidepressants with some evidence suggesting that they might be used to treat sleep disturbances among patients with PTSD were amitriptyline, paroxetine, sertraline, and nefazodone. Only short-term efficacy data are available for the aforementioned SSRIs, and there is a limited number of older, observational, and sub-optimally designed and reported studies indicating maintenance of therapeutic benefit for 6.5 months to 4 years for amitriptyline and nefazodone, respectively. Especially for nefazodone, only observational data support its use.

Our meta-analytic evaluation identified only amitriptyline to be associated with an improvement of sleep quality significantly greater than placebo, but the result was based on only one, rather old and small study [134], which warrants caution in the interpretation of this finding and limits its generalisability. We also identified that amitriptyline tended to cause less treatment-emergent insomnia compared to placebo, although this finding did not reach statistical significance. Based on the above, and with the obvious limitation of the scarcity of available data, our findings seem to support the common practice of the use of amitriptyline as a treatment of sleep disturbances, and indicate that it might be a useful treatment option for the clinician when sleep quality is a major problem.

Our meta-analysis identified a trend in favour of fluoxetine over placebo for the treatment of distressing dreams (nightmares resembling the trauma), which is the only noteworthy efficacy outcome, apart from amitriptyline's effect on sleep quality mentioned above. Again, this finding was based on only 1 RCT with a limited number of patients (N = 53).

**Fig. (8):**
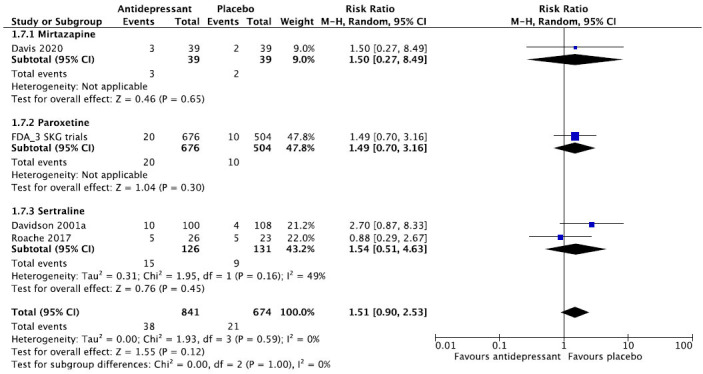
Response ratio for safety outcome ‘nightmares/vivid dreams’ of all antidepressants *versus* placebo. **Abbreviations:** N = Number, M-H = Maentel-Haenszel, CI = Confidence interval.

Our meta-analytic evaluation shows no significant effects of the comparisons between amitriptyline, venlafaxine, mirtazapine, and commonly prescribed SSRIs *vs.* placebo in terms of insomnia (Figs. **3** and **4**) and vivid dreams or nightmares (Fig. **8**). For the majority of individual comparisons, our findings were based on a limited number of patients from a small number of trials, or in some cases, even a single study, making most of the results inconclusive, with the exception of sertraline and paroxetine, for which larger numbers were available and whose results have been considered more robust (Figs. **4** and **8**). Paroxetine might be the only antidepressant causing more somnolence compared to placebo.

There is very limited evidence (1 RCT, N = 45) indicating that brofaromine may be associated with increased rates of insomnia, which can be a significant limiting factor to the overall clinical improvement of PTSD symptoms. Duloxetine was also found to be associated with the worsening of distressing dreams (nightmares resembling the trauma), but the quality of this evidence is very low (case report with psychiatric multi-morbidity and psychotropic polypharmacy), so this finding is not trustworthy and should not be used to guide clinical decision making.

As a guide for future research, we propose that:

1. Sleep-related PTSD symptoms should be specifically examined as study outcomes and reported as such in controlled trials.
2. Validated and PTSD-specific measures, such as the PSQI-A, should be used to assess response to treatment.
3. Concurrent treatment with other sedative psychotropic medication should be eliminated whenever possible, and if not, subgroup analyses should be performed and reported.
4. Terminology used to describe sleep-related symptoms (for example, nightmares resembling the trauma) and adverse events should be consistent, and more clarity on reporting relevant efficacy and safety outcomes is required in terms of definitions and methods used.
5. Designs should allow for reporting on subgroup analyses of response to treatment based on gender, trauma type, duration of illness, concurrent pharmacotherapy and psychotherapy, and/or psychosocial intervention, psychiatric comorbidity, especially major depression and substance misuse.
6. More and better-designed follow-up studies are needed to assess the effects on sleep of antidepressant treatment in the medium and the long term.
7. As an augmentation of treatment is common in clinical practice, more studies evaluating the therapeutic and adverse effects of antidepressants as add-on therapeutic agents for the treatment of sleep-related symptoms are required.
8. Designs that would allow for the identification of associations between clinical response and polysomnographic or actigraphy parameters would be very useful and may offer a better understanding of the mechanism of action of effective treatments, thereby deepening our understanding of the pathophysiology of sleep disorders in PTSD.
9. More better-designed studies, but also systematic reviews, meta-analyses, and network meta-analyses would be required to strengthen the evidence base and guide clinical practice.

## AUTHORS’ CONTRIBUTIONS

AL, NC, VPB, and MS designed the study. AL, ZP, and MS managed the literature search and extracted the data. AL and MS undertook the statistical analysis. AL, ZP, NC, VPB, and MS interpreted the data. AL and MS wrote the first draft of the manuscript. All authors contributed to and have approved the final manuscript.

## Figures and Tables

**Table 1:** Therapeutic and adverse effects of citalopram on sleep-related outcomes in patients with PTSD.

**First Author/** **Year**	**Study ** **Design**	**Population**	**Concurrent Intermittent Rescue Sleep Therapy**	**Dose (Range)**	**Outcomes**					
					**Efficacy**			**Safety**		
					**Total Sleep Time/** **Insomnia**	**Sleep Quality**	**Distressing Dreams/** **Nightmares**	**Insomnia**	**Somnolence**	**Vivid ** **Dreams/ Nightmares**
**Randomised Controlled Trials**					*Compared to Placebo*					
Tucker *et al.*, 2003 [28]	10-week double-blind, randomised, parallel-group clinical trial, monotherapy	N = 58 (cital = 25; sert = 23; pbo 10)	Allowed	Citalopram: 36.2 mg (20-50 mg) Sertraline: 134.1 mg (50-200 mg)	n.i.	n.i.	n.i.	= Cit 60% *vs.* sert 43.5% *vs.* pbo 70%	n.i.	n.i.
**B. Observational Studies**					*Compared to Baseline*					
English *et al.*, 2006 [46]	8-week open-label flexible-dose clinical trial, monotherapy	N = 18 (15 completers)	n.i.	Citalopram: 34.4 mg (20-40 mg)	n.i.	n.i.	n.i.	“Common side effects include insomnia” - not quantified	“Commonly associated side effects include somnolence” - not quantified, 1 drop out	n.i.
Seedat *et al.*, 2000 [47]	8-week open-label flexible-dose clinical trial, monotherapy	N = 14 (11 completers)	Not allowed	Citalopram: n.i. (20-40 mg)	n.i.	n.i.	n.i.	n.i.	45.5%	n.i.
Khouzam *et al.*, 2001 [48]	Case report	N = 2, follow-up 3-4 months	n.i.	Citalopram: n.i. (20-40 mg)	n.i.	50% (N = 1, marked improvement in his sleep at 20 mg daily after 3 months)	50% (N = 1, disappearance of trauma-related nightmares at 20 mg daily after 3 months)	n.i.	50% (N = 1 daytime sedation, dose moved to night-time and tolerated)	n.i.

**Abbreviations:** Sert = Sertraline, Pbo = Placebo, Cital = Citalopram, n.i. = no information, N = number of participants, mg = milligrams.

**Note:** For controlled trials: = no difference.

**Table 2:** Therapeutic and adverse effects of escitalopram on sleep-related outcomes in patients with PTSD.

**First ** **Author/** **Year**	**Study Design**	**Population**	**Concurrent Intermittent Rescue Sleep Therapy**	**Dose ** **(Range)**	**Outcomes**					
					**Efficacy**			**Safety**		
					**Total Sleep Time/** **Insomnia**	**Sleep ** **Quality**	**Vivid Dreams/** **Nightmares**	**Insomnia**	**Somnolence**	**Vivid Dreams/** **Nightmares**
**Observational Studies**					*Compared to baseline*					
Robert *et al.*, 2006 [54]	12-week open prospective trial, monotherapy	N = 25 (20 completers)	Not allowed	Escitalopram: n.i. (10-20 mg)	n.i.	= (PSQI, PSQI-A)	n.i.	12%	24%	4% odd dreams
Ramaswamy *et al.*, 2015 [55]	12-week open prospective trial, monotherapy	N = 11	Zolpidem	Escitalopram: n.i. (10-20 mg)	n.i.	n.i.	n.i.	27.3%	n.i.	n.i.
Qi *et al.*, 2017 [56]	3 and 6-month open clinical trial, monotherapy	N = 45 (36 completers)	n.i.	Escitalopram: n.i. (10-40 mg)	n.i.	n.i.	n.i.	2.8% (3 months) 0% (6 months)	11.1% (3 months) 2.8% (6 months)	n.i.

**Abbreviations:** n.i. = no information, N = number of participants, mg = milligrams, PSQI = Pittsburgh Sleep Quality Index, PSQI-A = Pittsburgh Sleep Quality Index-Addendum for PTSD.

**Note:** For observational studies: = no difference from baseline.

**Table 3:** Therapeutic and adverse effects of fluoxetine on sleep-related outcomes in patients with PTSD.

**First ** **Author/** **Year**	**Study Design**	**Population**	**Concurrent Intermittent Rescue Sleep ** **Therapy**	**Dose (Range)**	**Outcomes**					
					**Efficacy**			**Safety**		
					**Total Sleep ** **Time/** **Insomnia**	**Sleep ** **Quality**	**Vivid Dreams/** **Nightmares**	**Insomnia**	**Somnolence**	**Vivid Dreams/** **Nightmares**
**Randomised Controlled Trials**					-			-		
Martenyi *et al.*, 2002 [24]	24-week double-blind, randomised discontinuation trial following Martenyi 2002a, monotherapy	N = 131 (fluo 69; pbo 62)	n.i.	Fluoxetine: 53 mg (20-80 mg)	n.i.	n.i.	n.i.	= Fluo 15% *vs.* pbo 10%	n.i.	n.i.
Davidson *et al.*, 2005 [25]	Open-label treatment for 6 months, followed by a double-blind randomised trial of maintenance treatment for another 6 months, monotherapy	N = 62 (fluo 30; pbo 32)	n.i.	Fluoxetine: 48.6 mg (10-60 mg)	n.i.	n.i.	n.i.	= Fluo 16.7% *vs.* pbo 15.6%	n.i.	= Fluo 16.7% *vs.* pbo 18.8%
Onder *et al.* 2006 [30]	12-week open label randomised trial of fluoxetine, moclobemide, and tianeptine	N = 103 (fluo 38; mocl 35; tian 30)	Not allowed	Fluoxetine: (20-40 mg); Moclobemide (450-900 mg); tianeptine (37.5-50 mg)	n.i.	n.i.	n.i.	= Fluo 2.6%; mocl 2.9%; tian 0%	= Fluo 0%; mocl 0%; tian 3.3%	n.i.
Connor *et al.*, 1999 [58]	12-week double-blind, randomised clinical trial, monotherapy	N = 54 (fluo 27; pbo 27)	n.i.	Fluoxetine: 40 mg (10-60 mg)	n.i.	+ *(self-report)* = *(clinician-rated)*	=	n.i.	n.i.	n.i.
Martenyi *et al.*, 2002 [63]	12-week randomised clinical trial, monotherapy	N = 301 (fluo 226; pbo 75)	n.i.	Fluoxetine: 57 mg (20-80 mg)	n.i.	n.i.	n.i.	= Fluo 12% *vs.* pbo 12%	n.i.	n.i.
Martenyi *et al.*, 2007 [73]	12-week double-blind, randomised clinical trial, monotherapy	N = 411 (20 mg fluo 163; 40 mg fluo 160; pbo 88)	Chloral hydrate for insomnia	Fluoxetine: 20 mg & 40 mg	n.i.	=	n.i.	n.i.	= Fluo 10.5% *vs.* pbo 5.7%	n.i.
**Observational Studies**					*Compared to baseline*					
Shay *et al.*, 1991 [67]	An uncontrolled series arising from clinical practice; except for four patients who discontinued the drug, all have taken fluoxetine more than 12 months	N = 28 male veterans, also depressed	Allowed	Fluoxetine: n.i. (20-80 mg)	n.i.	n.i.	n.i.	57.1%	n.i.	n.i.
Nagy *et al.*, 1993 [69]	10-week open-label flexible-dose clinical trial of fluoxetine	N = 27 (19 completed at least 3 weeks and were analysed, 10 completers)	Allowed	Fluoxetine: n.i. (20-80 mg)	n.i.	+ (insomnia and difficulty falling asleep)	=	n.i.	n.i.	n.i.

**Abbreviations:** n.i. = no information, N = number of participants, mg = milligrams, fluo = fluoxetine, pbo = placebo, tian = tianeptine, mocl = moclobemide.

**Note:** For controlled trials: + antidepressant better than placebo/other antidepressant, = no difference.

For observational studies: + improvement compared to baseline, = no difference from baseline.

**Table 4:** Therapeutic and adverse effects of fluvoxamine on sleep-related outcomes in patients with PTSD.

**First ** **Author/** **Year**	**Study ** **Design**	**Population**	**Concurrent Intermittent Rescue Sleep Therapy**	**Dose ** **(Range)**	**Outcomes**					
					**Efficacy**			**Safety**		
					**Total Sleep Time/** **Insomnia**	**Sleep ** **Quality**	**Vivid Dreams/** **Nightmares**	**Insomnia**	**Somnolence**	**Vivid Dreams/** **Nightmares**
**Randomised Controlled Trials**										
Spivak *et al.*, 2006 [80]	8-week double-blind, randomised clinical trial, monotherapy	N = 40 (fluv 20; reb 20)	n.i.	Fluvoxamine: 150 mg Reboxetine: 8 mg	n.i.	n.i.	n.i.	= Fluv 0% *vs.* reb 20%	= Fluv 10% *vs.* reb 5%	n.i.
**Observational Studies**					*Compared to baseline*					
De Boer *et al.*, 1992 [74]	14-week open-label clinical trial, monotherapy	N = 24	Allowed	Fluvoxamine: n.i. (max 300 mg)	n.i.	64.7%	70.6%	n.i.	n.i.	n.i.
Davidson *et al.*, 1998 [75]	8-week open-label clinical trial	N = 15	n.i.	Fluvoxamine: 150 mg (50-300 mg)	n.i.	n.i.	n.i.	46%	n.i.	n.i.
Neylan *et al.*, 2001 [76,77]	10-week open-label clinical trial, monotherapy	N = 21	Allowed but those who received them were excluded from the analysis of sleep outcomes	Fluvoxamine: 150 mg (100-250 mg)	n.i.	85.7% (N = 18 for items trouble staying asleep and troubled sleep)	85.7% (N = 18 for item dreams about the combat trauma, but not for bad dreams)	14.3% (N = 3 used chloral hydrate or trazodone for night-time sedation)	n.i.	n.i.
Escalona *et al.*, 2002 [78]	14-week open-label clinical trial	N = 15	Allowed	Fluvoxamine: 150 mg (100-300 mg)	n.i.	n.i.	n.i.	73% (N = 11 used chloral hydrate or temazepam for night-time sedation)	n.i.	n.i.

**Abbreviations:** n.i. = no information, N = number of participants, mg = milligrams, fluv = fluvoxamine, reb = reboxetine.

**Note:** For controlled trials: = no difference.

**Table 5:** Effects of paroxetine on sleep quality; results from the NCT00202449 clinical trial [87].

**Outcome: Change in CAPS-Recurrent Distressing Dreams Item at Week 12**				
	**Paroxetine**	**Prazosin**	**Placebo**	***p*-value**
Number of participants analysed	7	9	9	n.i.
Mean (SD) (scale points)	-3.11 (2.52)	-1.29 (1.11)	-2.67 (3.0)	
**Outcome: Change in PSQI at Week 12**				
	**Paroxetine**	**Prazosin**	**Placebo**	***p*-value**
Number of participants analysed	9	6	9	n.i.
Mean (SD) (scale points)	-6.44 (3.91)	-2.33 (2.94)	-3.33 (5.45)	

**Abbreviations:** CAPS = Clinician-Administered PTSD Scale, PSQI = Pittsburgh Sleep Quality Index, SD = Standard Deviation, n.i. = no information.

**Table 6:** Therapeutic and adverse effects of paroxetine on sleep-related outcomes in patients with PTSD.

**First ** **Author/** **Year**	**Study ** **Design**	**Population**	**Concurrent Intermittent Rescue Sleep Therapy**	**Dose/** **Range**	**Outcomes**					
					**Efficacy**			**Safety**		
					**Total Sleep Time/** **Insomnia**	**Sleep ** **Quality**	**Vivid Dreams/** **Nightmares**	**Insomnia**	**Somnolence**	**Vivid Dreams/** **Nightmares**
**Pooled Analyses and Systematic Reviews and Meta-analyses**					*Compared to placebo*					
FDA data report [27] Stein *et al.*, [84] Sheehan *et al.*, [85]	Pooled analysis of 3 similarly designed 12-week placebo-controlled clinical trials	N = 1180, mixed civilian and combat PTSD	n.i.	Paroxetine: n.i. (20-50 mg)	+ On both clinician and patient-rated instruments, paroxetine was significantly more effective than placebo in reducing difficulty falling or staying asleep (CAPS item 13, *p < *.001; DTS item 13, *p *= .002)	+ On both clinician and patient-rated instruments, paroxetine was significantly more effective than placebo in reducing reduced overall sleep (MADRS item 4, *p *= .0021)	+ On both clinician and patient-rated instruments, paroxetine was significantly more effective than placebo in reducing the severity of distressing dreams (CAPS item 2, *p *= .053; DTS item 2, *p < *.001)	= Par 12% *vs.* pbo 10.9%	- Par 16% *vs.* pbo 5%	= Par 3% *vs.* pbo 2%
de Moraes Costa *et al.*, 2022 [86]	Network meta-analysis (only 1 trial with paroxetine included)	Direct data from NCT00202449			n.i.	=	=	n.i.	n.i.	n.i.
**Randomised Controlled Trials**										
Seo *et al.*, 2010 [34]	10-week open-label randomized clinical trial, monotherapy	N = 40 (par = 20; mir = 20)	No	Paroxetine: 38.89 mg (10-60 mg) Mirtazapine: 43.93 (10-60 mg)	n.i.	n.i.	n.i.	= Par 10% *vs.* mir 0%	n.i.	n.i.
Schneier *et al.*, 2012 [38]	10-week double-blind, randomized, placebo-controlled clinical trial, all participants were provided PET	N = 37 (par = 19; pbo = 18)	Zolpidem	Paroxetine: 32.2 mg (25-50 mg)	n.i.	n.i.	n.i.	= Par 5.3% *vs.* pbo 5.6%	= Par 10.5% *vs.* pbo 16.7%	n.i.
Simon *et al.*, 2008 [39]	10-week rater-blind, randomised, placebo-controlled clinical trial; all participants were provided PET	N = 23 who remained symptomatic after PET (par = 9; pbo = 14)	Trazodone, zolpidem, zaleplon	Paroxetine: 45.8 mg (12.5-62.5 mg)	n.i.	n.i.	n.i.	= Par 89% *vs.* pbo 85%	= Par 67% *vs.* pbo 77%	n.i.
Tucker *et al.*, 2001 [83]	12-week double-blind, randomised placebo-controlled clinical trial, monotherapy	N = 307 (par = 151; pbo = 156)	Chloral hydrate for the 1^st^ week	Paroxetine: 27.6 mg (20-50 mg)	n.i.	n.i.	n.i.	n.i.	- Par 17.2% *vs.* pbo 3.8%	n.i.
NCT00202449 [87]	12-week double-blind, randomised, placebo-controlled clinical trial, monotherapy	N = 59 (par = 20; pbo = 21; praz = 18)	n.i.	Paroxetine: 20 mg Prazosin: 4-30 mg	n.i.	= (Table 5)	= (Table 5)	n.i.	n.i.	n.i.
**Observational Studies**					*Compared to baseline*					
Tucker *et al.*, 2004 [89]	10-week, open-label prospective cohort study	22 patients with PTSD and comorbid depression	Diphenhydramine for at least 2 weeks	Paroxetine: n.i. (20-50 mg)	+ Hours of sleep showed a significant increase	n.i.	n.i.	n.i.	n.i.	n.i.

**Abbreviations:** n.i. = no information, N = number of participants, mg = milligrams, pbo = placebo, PET = prolonged exposure therapy.

**Note:** For controlled trials: + antidepressant better than placebo/other antidepressant, - worse than placebo/other antidepressant, = no difference.

For observational studies: + improvement compared to baseline.

**Table 7:** Therapeutic and adverse effects of sertraline on sleep-related outcomes in patients with PTSD.

**First ** **Author/** **Year**	**Study Design**	**Population**	**Concurrent Intermittent Rescue Sleep ** **Therapy**	**Dose ** **(Range)**	**Outcomes**						
					**Efficacy**			**Safety**			
					**Total Sleep Time/** **Insomnia**	**Sleep ** **Quality**	**Vivid Dreams/** **Nightmares**	**Insomnia**	**Somnolence**	**Vivid Dreams/** **Nightmares**
**Pooled Analyses and Evidence from Systematic Reviews and Meta-analyses**					*Compared to Placebo*						
de Moraes Costa, 2022 [86]	Network meta-analysis of RCTs	Direct data from Davidson *et al.*, 2001a; Friedman *et al.*, 2007; McRae *et al.*, 2004; and Schneier *et al.*, 2015			n.i.	+ (but pairwise results did not corroborate this finding)	+	n.i.	n.i.	n.i.
Davidson *et al.*, 2002 [96]	Pooled analysis of 2 RCTs [94, 95]	N = 385 (sert 191; pbo 194)	n.i.	133.3-146.3 mg (50-200 mg)	n.i.	+	=	n.i.	n.i.	n.i.
**Randomised Controlled Trials**											
NCT03033069 [26]	12-week double-blind, randomised, placebo-controlled clinical trial, monotherapy or combination	N = 164 (sert 81; pbo 83)	Not allowed	Sertraline: (50-200 mg)	n.i.	n.i.	n.i.	= Sert 4.9% *vs.* pbo 3.6%	= Sert 3.7% *vs.* pbo 8.4%	n.i.
		N = 157 (brex+sert 82; brex+pbo 75)			n.i.	n.i.	n.i.	= Sert 2.4% *vs.* pbo 6.7%	= Sert 3.7% *vs.* pbo 6.7%	n.i.
Tucker *et al.*, 2003 [28]	10-week double-blind, randomised, placebo-controlled clinical trial, monotherapy	N = 58 (sert 23; citalopram 25; pbo 10)	Allowed	Sertraline: 134.1 mg (50-200 mg) Citalopram: 36.2 mg (20-50 mg)	n.i.	n.i.	n.i.	= Sert 43.5% *vs.* cit 60% *vs.* pbo 70%	n.i.	n.i.
Davidson *et al.* 2006 [29]	12-week double-blind, randomised, placebo-controlled clinical trial, monotherapy	N = 531 (sert 173; venl 179; pbo 179)	Only for 2 weeks after baseline	Sertraline: 110.2 mg (50- 200 mg) Venlafaxine: 164.4 mg (75-300 mg)	n.i.	n.i.	n.i.	= Sert 10.4%, venl 13.4% *vs.* pbo 8.9%	= Sert 10.4%, venl 11.7% *vs.* pbo 13.4%	n.i.
Chung *et al.*, 2004 [32]	6-week open-label, randomised clinical trial, monotherapy	N = 113 (sert 55; mirt 58)	Zopiclone for insomnia	Sertraline: 101.5 mg (15-50 mg) Mirtazapine 34.1 mg (15-50 mg)	n.i.	Sert 9.1% *vs.* mirt 3.4% (use of zopiclone)	n.i.	n.i.	n.i.	n.i.
McRae *et al.*, 2004 [33]	12-week double-blind, randomised, placebo-controlled clinical trial, monotherapy	N = 37 (sert 19; nef 18)	Not allowed	Sertraline: 153 mg (max 200 mg) Nefadozone 463 mg (600 mg)	n.i.	= (improved compared to baseline but no difference between the drugs)	n.i.	= Sert 16.7% *vs.* nef 21.1%	= Sert 27.8% *vs.* nef 26.3%	n.i.
Rauch *et al.*, 2019 [40]	24-week single-blind, randomised, placebo-controlled clinical trial, add-on to PE	N = 223, data for a subgroup of 149 participants (*sert plus PET 51; pbo plus PE 42; sert only 56)*	Allowed	Sertraline plus PE: 171.6 mg (50-200 mg)	n.i.	Persistence of difficulty sleeping Sert plus PE 71.5% *vs.* pbo plus PE 82.1%	Persistence of nightmares Sert plus PE 60% *vs.* pbo plus PE 75%	n.i.	n.i.	n.i.
Roache *et al.*, 2017 [41]	12-week double-blind, randomized, placebo-controlled clinical trial, add-on to CBT	N = 49 (sert 26; pbo 23)	n.i.	Sertraline 150 mg (fixed dose)	n.i.	n.i.	n.i.	= Sert 23.1% *vs.* pbo 13%	= Sert 7.7% *vs.* pbo 13%	= Sert 19.2% *vs.* pbo 21.7%
Brady *et al.*, 2000 [94]	12-week double-blind, randomised, placebo-controlled clinical trial, monotherapy	N = 187 (sert 94; pbo 93)	n.i.	Sertraline: 133.3 mg (50- 200 mg)	n.i.	n.i.	n.i.	- (sert 16% *vs.* pbo 4.3%)	= (sert 12.8% *vs.* pbo 9.8%)	n.i.
Davidson *et al.*, 2001 [95]	12-week double-blind, randomised placebo-controlled clinical trial, monotherapy	N = 208 (sert 100; pbo 108)	n.i.	Sertraline: 146.3 mg (50-200 mg)	n.i.	= PSQI	n.i.	- Sert 35% s *vs.* pbo 22%	= Sert 17% *vs.* pbo 11%	= Sert 10% *vs.* pbo 4%
Friedman *et al.*, 2007 [97]	12-week double-blind, randomised, placebo-controlled clinical trial, monotherapy	N = 169 (sert 86; pbo 83)	Chloral hydrate for insomnia	Sertraline: 135 mg (50-200 mg)	n.i.	=	n.i.	= Sert 14% *vs.* pbo 9.6%	= Sert 14% *vs.* pbo 8.4%	n.i.
Li *et al.*, 2017 [99]	12-week double-blind, randomised, placebo-controlled clinical trial, monotherapy	N = 72 (sert 36; pbo 36)	n.i.	Sertraline: 135 mg (50-200 mg)	n.i.	n.i.	n.i.	= Sert 27.8% *vs.* pbo 19.4%	= Sert 25% *vs.* pbo 13.9%	n.i.
Panahi *et al.*, 2011 [100]	10-week double-blind, randomised, placebo-controlled clinical trial, monotherapy	N = 70 (sert 35; pbo 35)	Allowed	Sertraline: 140 mg (50-200 mg)	n.i.	n.i.	n.i.	= Sert 28.6% *vs.* pbo 11.4%	= Sert 14.3% *vs.* pbo 5.7%	n.i.
Zohar *et al.*, 2001 [101]	10-week double-blind, randomised, placebo-controlled clinical trial, monotherapy	N = 42 (sert 23; pbo 19)	Allowed	Sertraline: 120 mg (50-200 mg)	n.i.	n.i.	n.i.	n.i.	= Sert 26.1% *vs.* pbo 15.8%	n.i.
Robb *et al.*, 2010 [102]	10-week double-blind, randomised, placebo-controlled clinical trial, monotherapy in children and adolescents (ages 6-17 years)	N = 129 (sert 67; pbo 62)	Diphenhydramine or chloral hydrate for sleep	Sertraline plus 104 mg (50-200 mg)	n.i.	n.i.	n.i.	= Sert 10.4% *vs.* pbo 12.9%	n.i.	n.i.
**Observational Studies**					*Compared to Baseline*			-		
Kamo *et al.*, 2016 [103]	A retrospective medical chart review of patients with PTSD, caused by various types of trauma	N = 122	Yes	Sertraline: 12.5-150 mg	n.i.	n.i.	n.i.	n.i.	9%	n.i.

**Abbreviations:** n.i. = no information, N = number of participants, mg = milligrams, sert = sertraline, brex = brexpiprazole, pbo = placebo, PET = prolonged exposure therapy.

**Note:** For controlled trials: + antidepressant better than placebo/other antidepressant, - worse than placebo/other antidepressant, = no difference.

**Table 8:** Therapeutic and adverse effects of duloxetine on sleep-related outcomes in patients with PTSD.

**First ** **Author/** **Year**	**Study ** **Design**	**Population**	**Concurrent Intermittent Rescue Sleep ** **Therapy**	**Dose (Range)**	**Outcomes**					
					**Efficacy**			**Safety**		
					**Total Sleep Time/** **Insomnia**	**Sleep ** **Quality**	**Vivid Dreams/** **Nightmares**	**Insomnia**	**Somnolence**	**Vivid Dreams/** **Nightmares**
**Observational Studies**					*Compared to baseline*					
Walderhaug *et al.*, 2010 [105]	8-week open-label trial, monotherapy	N = 21 males with chronic treatment-refractory, combat-related PTSD	Not allowed	Duloxetine: 108 mg (30-120 mg)	n.i.	n.i.	+	55% (sleep disturbance)	50% (sleepiness/ sedation)	65% (increased dream activity without nightmares)
Villarreal *et al.*, 2010 [106]	12-week open-label trial, monotherapy	N = 20 male veterans, 15 completed	Allowed	Duloxetine: 81 mg (30-120 mg)	n.i.	+	n.i.	n.i.	n.i.	n.i.
Deneys and Ahearn, 2006 [107]	Case report	N = 1	n.i.	Duloxetine: 60 mg	n.i.	n.i.	- within the first week, the patient experienced a severe exacerbation of nightmares	n.i.	n.i.	n.i.

**Abbreviations:** n.i. = no information, N = number of participants, mg = milligrams.

**Note:** For observational studies: + improvement compared to baseline, - deterioration compared to baseline.

**Table 9:** Therapeutic and adverse effects of venlafaxine on sleep-related outcomes in patients with PTSD.

**First Author/** **Year**	**Study ** **Design**	**Population**	**Concurrent Intermittent Rescue** ** Sleep ** **Therapy**	**Dose ** **(Range)**	**Outcomes**					
					**Efficacy**			**Safety**		
					**Total Sleep Time/** **Insomnia**	**Sleep ** **Quality**	**Vivid Dreams/** **Nightmares**	**Insomnia**	**Somnolence**	**Vivid Dreams/** **Nightmares**
**Pooled Analyses and Evidence from Systematic Reviews and ** **Meta-analyses**					*Compared to placebo*					
Stein *et al.*, 2009 [110]	Pooled analysis of 2 RCTs [29,98]	N = 687 (venl 340; pbo 347)	n.i.	Venlafaxine: 223.1 mg (75-300 mg)	n.i.	= (difficulty falling or staying asleep)	= (distressing dreams)	n.i.	n.i.	n.i.
**Randomised Controlled Trials**										
Davidson *et al.*, 2006 [29]	12-week double-blind, randomised clinical trial, monotherapy	N = 531 (venl 179; sert 173; pbo 179)	Only for 2 weeks after baseline	Venlafaxine: 225 mg (75-300 mg) Sertraline: 110.2 mg (50- 200 mg)	n.i.	n.i.	n.i.	= Venl 13.4%, sert 10.4% *vs.* pbo 8.9%	= Venl 11.7%, sert 10.4% *vs.* pbo 13.4%	n.i.
Davidson *et al.*, 2006 [98]	6-month, double-blind, placebo-controlled trial, monotherapy	N = 329 (venl 161; pbo 168)	n.i.	Venlafaxine: 221.5 mg (75-300 mg)	n.i.	n.i.	n.i.	= Venl 7.5% *vs.* pbo 10.1%	= Venl 5.6% *vs.* pbo 5.4%	n.i.

**Abbreviations:** n.i. = no information, N = number of participants, mg = milligrams.

**Note:** For controlled trials: = no difference.

**Table 10:** Therapeutic and adverse effects of nefazodone on sleep-related outcomes in patients with PTSD.

**First** **Author/** **Year**	**Study ** **Design**	**Population**	**Concurrent Intermittent Rescue sleep ** **Therapy**	**Dose ** **(Range)**	**Outcomes**					
					**Efficacy**			**Safety**		
					**Total Sleep ** **Time/** **Insomnia**	**Sleep ** **Quality**	**Vivid ** **Dreams/** **Nightmares**	**Insomnia**	**Somnolence**	**Vivid Dreams/** **Nightmares**
**Pooled Analyses and Evidence from Systematic Reviews and Meta-analyses (Compared to Baseline)**										
de Moraes Costa *et al.*, 2022 [86]	Network meta-analysis (NMA) of placebo-controlled RCTs	Data from Mc Rae *et al.*, 2004	Data from Mc Rae *et al.*, 2004	Data from Mc Rae *et al.*, 2004	n.i.	=	=	n.i.	n.i.	n.i.
Hidalgo *et al.*, 1999 [113]	Pooled analysis of 6 observational trials	N = 105, mixed civilian and combat (71.4%) PTSD	n.i.	272-583 mg (50-600 mg)	+ (unpublished data only, from Tucker *et al.*, 1998)	+ (sleep troubles and HDS-sleep items)	+	n.i.	n.i.	n.i.
**Randomised Controlled Trials (Compared to Placebo)**										
Mc Rae *et al.*, 2004 [33]	12-week double-blind, randomized clinical trial, monotherapy	N = 37 (sert 19; nef 18)	Not allowed	Sertraline: 153 mg (max 200 mg) Nefazodone: 463 mg (600 mg)	n.i.	= (improved compared to baseline but no difference between the drugs)	n.i.	= Sert 16.7% *vs.* nef 21.1%	= Sert 27.8% *vs.* nef 26.3%	n.i.
**Observational Studies (Compared to Baseline)**										
Davidson *et al.*, 1998 [116]	12-week open-label clinical trial, monotherapy	N = 17 (10 completers) with chronic civilian PTSD	n.i.	Nefazodone: 386 mg (max 600 mg)	n.i.	62.5% (response rate for completers)	50% (response rate for completers)	n.i.	31.3%	n.i.
Gillin *et al.*, 2001 [117]	12-week open-label clinical trial, monotherapy	N = 12 male veterans (subgroup obtained from another study [122])	n.i.	Nefazodone: 441 mg (max 600 mg)	= (6.2 hrs/ night; polysomnographic measures did not change significantly)	+	+	n.i.	n.i. some patients reported increased daytime sedation	n.i.
Hertzberg *et al.*, 1998 [118]	12-week open-label clinical trial, monotherapy	N = 10 male veterans	Not allowed	Nefazodone: 490 mg (300-600 mg)	+ (6.8 hrs/ night)	+	n.i.	n.i.	n.i.	n.i.
Hertzberg *et al.*, 2002 [119]	Follow-up data over 3-4 years of Hertzberg’s study, 1998	N = 10 male veterans	Six patients remained only on nefazodone; the other four were prescribed other drugs	Nefazodone: (400-600 mg)	= (benefit maintained, 5.8 hrs/night)	= (benefit maintained)	n.i.	n.i.	n.i.	n.i.
Mellman *et al.*, 1999 [120]	6-week open-label clinical trial, monotherapy	N = 15 with chronic PTSD (13 male veterans)	Not allowed	Nefazodone: 272.5 mg (125-500 mg)	+ (5.7 hrs/ night)	n.i.	= (dream distress did not change significantly, but at 6 weeks, all patients reported dreams not replicating trauma anymore)	n.i.	n.i.	n.i.
Neylan *et al.*, 2003 [121]	12-week open-label clinical trial, monotherapy	N = 10 male veterans	Not allowed	Nefazodone: 570 mg (500-600 mg)	+ (7.7 hrs/ night)	+	+	n.i.	n.i.	n.i.
Zisook *et al.*, 2000 [122]	12-week open-label clinical trial, monotherapy	N = 19 male veterans (15 completers)	Not allowed	Nefazodone: 424 mg (100-600 mg)	+ (5.9 hrs/ night)	+	+	n.i.	37%	n.i.

**Abbreviations:** n.i. = no information, N = number of participants, mg = milligrams, sert = sertraline, nef = nefazodone.

**Note:** For controlled trials: + antidepressant better than placebo/other antidepressant, = no difference.

For observational studies: + improvement compared to baseline, = no difference from baseline.

**Table 11:** Therapeutic and adverse effects of trazodone on sleep-related outcomes in patients with PTSD.

**First Author/** **Year**	**Study Design**	**Population**	**Concurrent Intermittent Rescue Sleep Therapy**	**Dose ** **(Range)**	**Outcomes**							
					**Efficacy**					**Safety**		
					**Total Sleep Time/** **Insomnia**	**Sleep ** **Quality**		**Vivid Dreams/** **Nightmares**		**Insom-nia**	**Somnolence**	**Vivid Dreams/** **Nightmares**
**Observational Studies**					*Compared to Baseline*							
Ashford *et al.*, 1996 [126]	3-month open-label clinical trial	N = 57 exposed to war trauma (N = 30 with PTSD diagnosis, 29 returned results, Ν = 19 < 60 yo, N = 10 > 60yo)	Implied but not specified	Trazodone: 25-500 mg	+ (reported improved initial insomnia but not quantified)	+		+		n.i.	n.i.	n.i.
						<60yo	>60yo	<60yo	>60yo			
						100% of PTSD patients	33% of PTSD patients	37% complete resolution 63% at least 75% improvement (percentage refers to the intensity of the symptom)	33.3% complete resolution			
Hertzberg *et al.*, 1996 [127]	4-month open-label clinical trial with up to 3 months follow-up evaluation (quasi-waiting list condition but no formal statistical analysis), monotherapy	N = 6 (6 completers at 4 months, N = 5 completers of follow-up evaluation)	Not allowed	Trazodone: 300 mg (50-400 mg)	+ (mean sleep time = 3.8 ± 1h at baseline *vs.* 6.0 ± 1.4h at endpoint, maintained at follow-up; no statistical analysis)	+ (PSQI_baseline_ = 12.8 ± 1.8 *vs.* PSQI_endpoint (4 months)_ = 7.8 ± 3.1, improvement tended to persist at follow-up; no statistical analysis)		n.i.		n.i.	n.i.	n.i.
Warner *et al.*, 2001 [128]	8-week inpatient program, self-reported survey, empirically developed by the authors; add-on trazodone therapy for sleep and nightmares	N = 74 (N = 60 completers, N = 55 for whom trazodone was prescribed specifically for the treatment of nightmares)	97% on other antidepressants (fluoxetine, paroxetine, sertraline nefazodone), 28% on valproic acid, 13% on benzodiazepines, 10% on antipsychotics (olanzapine, risperidone)	Trazodone: 212 mg (25-600 mg, most patients 50-200 mg daily)	+ (N = 60) 100% reported improved overall sleep, 92% reported improved initial insomnia, 78% reported improved maintenance insomnia	n.i.		+ (N = 55) 73% reported moderate to significant improvement in nightmares Nights/week with nightmares pre-treatment = 3.3 ± 1.7 *vs.* 1.3 ± 1.4 post-treatment, *p < *0.005		n.i.	49.1% (also 5 dropouts due to daytime sedation)	1 drop out due to vivid nightmares (not clear if these had any resemblance to the index trauma)

**Abbreviations:** n.i. = no information, N = number of participants, mg = milligrams.

**Note:** For observational studies: + improvement compared to baseline.

**Table 12:** Therapeutic and adverse effects of reboxetine on sleep-related outcomes in patients with PTSD.

**First Author/** **Year**	**Study Design**	**Population**	**Concurrent Intermittent Rescue Sleep Therapy**	**Dose ** **(Range)**	**Outcomes**					
					**Efficacy**			**Safety**		
					**Total Sleep Time/** **Insomnia**	**Sleep ** **Quality**	**Vivid Dreams/** **Nightmares**	**Insomnia**	**Somnolence**	**Vivid Dreams/** **Nightmares**
**Randomised Controlled Trials**										
Spivak *et al.*, 2006 [80]	8-week double-blind, randomised clinical trial, monotherapy	N = 40 (fluvo 20; reb 20)	n.i.	Fluvoxamine: 150 mg Reboxetine: 8 mg	n.i.	n.i.	n.i.	= Fluv 0% *vs.* reb 20%	= Fluv 10% *vs.* reb 5%	n.i.

**Abbreviations:** n.i. = no information, N = number of participants, mg = milligrams, reb = reboxetine, fluvo = fluvoxamine.

**Note:** For controlled trials: = no difference.

**Table 13:** Therapeutic and adverse effects of amitriptyline on sleep-related outcomes in patients with PTSD.

**First ** **Author/** **Year**	**Study Design**	**Population**	**Concurrent Intermittent Rescue Sleep Therapy**	**Dose ** **(Range)**	**Outcomes**					
					**Efficacy/Effectiveness**			**Safety/Tolerance/Adverse Effects**		
					**Total Sleep Time/** **Insomnia**	**Sleep ** **Quality**	**Vivid Dreams/** **Nightmares**	**Insomnia**	**Sedation**	**Vivid Dreams/** **Nightmares**
**Randomised Controlled Trials**										
Davidson *et al.*, 1993 [134]	8-week double-blind, randomised clinical trial, monotherapy	N = 62 (ami 33; pbo 29)	Chloral hydrate	Amitriptyline 158.3 mg (50-300 mg)	n.i.	+	n.i.	= Ami 4% *vs.* pbo 22%	= Ami 67% *vs.* pbo 50%	n.i.
**B. Observational Studies**					*Compared to Baseline*					
Falcon *et al.*, 1985 [135]	Concurrent and retrospective chart review	N = 10 male veterans	n.i.	Amitriptyline: n.i. (150-250 mg)	n.i.	n.i.	+	n.i.	n.i.	n.i.
Bleich *et al.*, 1986 [136]	Retrospective review of clinical notes of patients referred to a PTSD unit treated for an average of 6.5 months	N = 14 received amitriptyline	n.i.	Amitriptyline: 139 mg	n.i.	+ 13 patients improved; the most prominent beneficial effects were reported on sleep and traumatic dreams	+	n.i.	n.i.	n.i.
Başoǧlu *et al.*, 1992 [137]	Case report, 8-month follow-up	N = 1, severe torture-related PTSD	no	Amitriptyline 150mg daily	+ (marked improvement)	n.i.	+ (nightmares disappeared)	n.i.	n.i.	n.i.

**Abbreviations:** n.i. = no information, N = number of participants, mg = milligrams, ami = amitriptyline, pbo = placebo.

**Note:** For controlled trials: + antidepressant better than placebo/other antidepressant, = no difference.

For observational studies: + improvement compared to baseline.

**Table 14:** Therapeutic and adverse effects of clomipramine on sleep-related outcomes in patients with PTSD.

**First Author/** **Year**	**Study Design**	**Population**	**Concurrent Intermittent Rescue Sleep Therapy**	**Dose ** **(Range)**	**Outcomes**					
					**Efficacy**			**Safety**		
					**Total Sleep Time/** **Insomnia**	**Sleep Quality**	**Vivid Dreams/** **Nightmares**	**Insomnia**	**Somnolence**	**Vivid Dreams/** **Nightmares**
**A. Observational Studies**					*Compared to Baseline*					
Bleich *et al.*, 1986 [136]	Retrospective review of clinical notes of patients referred to a PTSD unit treated for an average of 6.5 months	N = 2 received clomipramine	n.i.	Clomipramine: 150 mg	n.i.	+ 1 patient improved moderately; in those patients who responded, the most prominent beneficial effects were on sleep and traumatic dreams	+	n.i.	n.i.	n.i.

**Abbreviations:** n.i. = no information, N = number of participants, mg = milligrams.

**Note:** For observational studies: + improvement compared to baseline.

**Table 15:** Therapeutic and adverse effects of desipramine on sleep-related outcomes in patients with PTSD.

**First ** **Author/** **Year**	**Study ** **Design**	**Population**	**Concurrent Intermittent Rescue Sleep Therapy**	**Dose ** **(Range)**	**Outcomes**					
					**Efficacy**			**Safety**		
					**Total Sleep Time/Insomnia**	**Sleep ** **Quality**	**Vivid Dreams/** **Nightmares**	**Insomnia**	**Somnolence**	**Vivid Dreams/** **Nightmares**
**A. Observational Studies**					*Compared to Baseline*					
Falcon *et al.*, 1985 [135]	6-8-week, observational trial	N = 7 received desipramine	n.i.	Desipramine: (200-250 mg)	n.i.	n.i.	+	n.i.	n.i.	n.i.

**Abbreviations:** n.i. = no information, N = number of participants, mg = milligrams.

**Note:** For observational studies: + improvement compared to baseline.

**Table 16:** Therapeutic and adverse effects of doxepin on sleep-related outcomes in patients with PTSD.

**First ** **Author/** **Year**	**Study ** **Design**	**Population**	**Concurrent Intermittent Rescue Sleep Therapy**	**Dose ** **(Range)**	**Outcomes**					
					**Efficacy/Effectiveness**			**Safety**		
					**Total Sleep Time/** **Insomnia**	**Sleep ** **Quality**	**Vivid Dreams/** **Nightmares**	**Insomnia**	**Somnolence**	**Vivid Dreams/** **Nightmares**
**Observational Studies**					*Compared to Baseline*					
Falcon *et al.*, 1985 [135]	Concurrent and retrospective chart review	N = 1 male veteran	n.i.	Doxepin: 100 mg	n.i.	n.i.	+	n.i.	n.i.	n.i.
Bleich *et al.*, 1986 [136]	Retrospective review of clinical notes of patients referred to a PTSD unit treated for an average of 6.5 months	N = 7 male veterans	n.i.	Doxepin: 100 mg	n.i.	+ 4/7 patients improved at least moderately; in those patients who responded, the most prominent beneficial effects were on sleep and traumatic dreams	+	n.i.	n.i.	n.i.
Boehnlein *et al.*, 1985 [142]	Retrospective review of clinical notes of patients one year after receiving the diagnosis	N = 12 cambodian refugees, 6 received doxepin (4 monotherapy)	Allowed	Doxepin: n.i. (50-150 mg)	n.i.	+ (83% improved)	+ (67% improved)	n.i.	n.i.	n.i.
White, 1983 [143]	Open-label trial	N = 18	n.i.	Doxepin n.i. (25-100 mg)	n.i.	+ Good effect on sleep, but benzodiazepine use limits the interpretation of results	n.i.	n.i.	n.i.	n.i.

**Abbreviations:** n.i. = no information, N = number of participants, mg = milligrams.

**Note:** For observational studies: + improvement compared to baseline.

**Table 17:** Therapeutic and adverse effects of imipramine on sleep-related outcomes in patients with PTSD.

**First ** **Author/** **Year**	**Study ** **Design**	**Population**	**Concurrent Intermittent Rescue Sleep ** **Therapy**	**Dose ** **(Range)**	**Outcomes**					
					**Efficacy**			**Safety**		
					**Total ** **Sleep Time/** **Insomnia**	**Sleep ** **Quality**	**Vivid ** **Dreams/** **Nightmares**	**Insomnia**	**Somnolence**	**Vivid Dreams/** **Nightmares**
**Observational Studies**					*Compared to Baseline*					
Falcon *et al.*, 1985 [135]	Concurrent and retrospective chart review	N = 2 male veterans	n.i.	Imipramine: n.i. (150-200 mg)	n.i.	n.i.	+	n.i.	n.i.	n.i.
Boehnlein *et al.*, 1985 [142]	Retrospective review of clinical notes of patients one year after receiving the diagnosis	N = 12 Cambodian refugees, 5 received imipramine (3 monotherapy)	Allowed	Imipramine: 75-150 mg	n.i.	+ (75% improved)	+ (80% improved)	n.i.	n.i.	n.i.
Burstein *et al.*, 1983 [144]	Case reports, 4 months	N = 5	n.i.	Imipramine 200-300 mg	n.i.	+ (deepening of sleep, all responded within 5 days)	n.i.	n.i.	n.i.	n.i.
Burstein *et al.*, 1984 [145]	Case reports, 2-3 weeks	N = 15 (10 completers)	n.i.	Imipramine: 260 mg (50-350 mg)	n.i.	+	+	n.i.	n.i.	n.i.
Kinzie *et al.*, 1989 [146]	Prospective open study	N = 11 Cambodian refugees with chronic PTSD and depressive disorder	n.i.	Imipramine 50-150 mg + Clonidine 0.1-0.6 mg daily (N = 9) Or Imipramine monotherapy 50-150 mg (N = 2, data not provided for sleep outcomes, just a general comment that symptoms improved enough not to require augmentation with clonidine)	n.i.	22.2% improved (2 out of 9)	33.3% improved (3 out of 9)	n.i.	n.i.	n.i.

**Abbreviations:** n.i. = no information, N = number of participants, mg = milligrams.

**Note:** For observational studies: + improvement compared to baseline.

**Table 18:** Therapeutic and adverse effects of tianeptine on sleep-related outcomes in patients with PTSD.

**First ** **Author/** **Year**	**Study ** **Design**	**Population**	**Concurrent Intermittent Rescue Sleep ** **Therapy**	**Dose (Range)**	**Outcomes**					
					**Efficacy**			**Safety**		
					**Total Sleep Time/** **Insomnia**	**Sleep Quality**	**Vivid Dreams/** **Nightmares**	**Insomnia**	**Somnolence**	**Vivid Dreams/** **Nightmares**
**Randomised Controlled Trials**										
Önder *et al.*, 2006 [30]	12-week open-label randomised trial of fluoxetine, moclobemide, and tianeptine	N = 103 (fluo 38; mocl 35; tian 30)	Not allowed	Fluoxetine: (20-40 mg); Moclobemide (450-900 mg); tianeptine (37.5-50 mg)	n.i.	n.i.	n.i.	= Fluo 2.6%; mocl 2.9%; tian 0%	= Fluo 0%; mocl 0%; tian 3.3%	n.i.

**Abbreviations:** n.i. = no information, N = number of participants, mg = milligrams, fluo = fluoxetine, pbo = placebo, tian = tianeptine, mocl = moclobemide.

**Note:** For controlled trials: = no difference.

**Table 19:** Therapeutic and adverse effects of mirtazapine on sleep-related outcomes in patients with PTSD.

**First Author/** **Year**	**Study Design**	**Population**	**Concurrent ** **Intermittent Rescue Sleep Therapy**	**Dose ** **(Range)**	**Outcomes**					
					**Efficacy**			**Safety**		
					**Total ** **Sleep ** **Time/** **Insomnia**	**Sleep ** **Quality/** **Insomnia**	**Vivid Dreams/** **Nightmares**	**Insomnia**	**Somnolence**	**Vivid Dreams/** **Nightmares**
**Systematic Reviews and Meta-analyses**										
de Moraes Costa, 2022 [86]	Network meta-analysis of randomised controlled trials	Direct data from Schneier *et al.*, 2015			n.i.	= (compared to placebo)	+ (compared to placebo)	n.i.	n.i.	n.i.
**Randomised Controlled Trials - Mirtazapine Monotherapy**										
Chung *et al.*, 2004 [32]	6-week open-label, randomised clinical trial, monotherapy	N = 113 (sert 55; mirt 58)	Zopiclone for insomnia	Sertraline: 101.5 mg (15-50 mg) Mirtazapine 34.1 mg (15-50mg)	n.i.	Sert 9.1% *vs.* mirt 3.4% (use of zopiclone)	n.i.	n.i.	n.i.	n.i.
Davis *et al.*, 2020 [154]	8-week double-blind, randomised, placebo-controlled clinical trial, monotherapy	N = 78 (mirt = 39; pbo = 39)	Temazepam (n = 2 mirt / n = 5 pbo) Lorazepam (n = 1 mirt / n = 3 pbo) Trazodone (n = 0 mirt / n = 3 pbo)	Mirtazapine: 38.5 mg (15-45 mg)	n.i.	= (PSQI, but 28.2% required treatment for acute insomnia in Pbo *vs.* 7.7% in the Mirt group)	n.i.	+ Mir 7.7% *vs.* pbo 28.2%	= Mir 0% *vs.* pbo 7.7%	= Mir 7.7% *vs.* pbo 5.1%
Davidson *et al.*, 2003 [155]	8-week double-blind, randomised, placebo-controlled clinical trial, monotherapy	N = 26 (mirt = 17; pbo = 9)	n.i.	Mirtazapine: n.i. (15-45 mg/day)	n.i.	n.i.	n.i.	n.i.	Mir 1 participant; no information for pbo	n.i.
**Randomised Controlled Trials - Mirtazapine as add-on Treatment**										
Schneier *et al.*, 2015 [37]	24-week double-blind, randomised, placebo-controlled clinical trial, as add-on to sertraline	N = 38 (sert+mirt = 18; sert+pbo = 20)	Not allowed	Mirtazapine: 32.5 mg (30-45 mg) Sertraline: 118.1 mg for sert+mirt and 122.2 mg for sert+pbo (25-200 mg)	n.i.	= (PSQI)	n.i.	= Mir 15.4% *vs.* pbo 14.3%	= Mir 46.2% *vs.* pbo 35.7%	n.i.
**Observational Studies - Mirtazapine Monotherapy**					*Compared to baseline*					
Connor *et al.*, 1999 [156]	8-week pilot trial, monotherapy	N = 6 mixed (3 completers)	n.i.	Mirtazapine: n.i, (15-45 mg)	n.i.	n.i.	n.i.	16.7%	16.7%	n.i.
**Observational Studies - Mirtazapine as add-on Treatment**										
Lewis *et al.*, 2002 [153]	Letter to the editor, add-on therapy to an SSRI and an anxiolytic	More than 300 refugees	n.i.	Mirtazapine: n.i.	n.i.	+ (insomnia) 75% of participants	+ 75% of participants	n.i.	n.i.	n.i.

**Abbreviations:** n.i. = no information, N = number of participants, mg = milligrams, MIR = mirtazapine, pbo = placebo, SER = sertraline, PSQI = Pittsburgh Sleep Quality Index.

**Note:** For controlled trials: + antidepressant better than placebo/other antidepressant, = no difference.

For observational studies: + improvement compared to baseline.

**Table 20:** Therapeutic and adverse effects of brofaromine on sleep-related outcomes in patients with PTSD.

**First ** **Author/** **Year**	**Study Design**	**Population**	**Concurrent Intermittent Rescue Sleep Therapy**	**Dose ** **(Range)**	**Outcomes**					
					**Efficacy**			**Safety**		
					**Total Sleep Time/** **Insomnia**	**Sleep ** **Quality**	**Vivid ** **Dreams/** **Nightmares**	**Insomnia**	**Somnolence**	**Vivid Dreams/** **Nightmares**
**Randomised Controlled Trials**										
Katz *et al.*, 1994 [159]	14-week double-blind, randomised clinical trial, monotherapy	N = 64 randomised, 45 met criteria (brof 22; pbo 23)	Chloral hydrate and short-acting benzodiazepine hypnotics for sleep	Brofaromine n.i. (50-150 mg)	n.i.	n.i. “Insomnia following drug therapyrepresented the only noteworthy limitation to clinical improvement, since insomnia is also an aspect of PTSD, which is included in the CAPS”	n.i.	= Brof 34.3% *vs.* pbo 12.1%	n.i.	n.i.

**Abbreviations:** n.i. = no information, N = number of participants, mg = milligrams, brof = brofaromine, pbo = placebo CAPS = Clinician Administered PTSD Scale.

**Note:** For controlled trials: = no difference.

**Table 21:** Therapeutic and adverse effects of moclobemide on sleep-related outcomes in patients with PTSD.

**First ** **Author/** **Year**	**Study ** **Design**	**Population**	**Concurrent Intermittent Rescue Sleep Therapy**	**Dose (Range)**	**Outcomes**					
					**Efficacy**			**Safety**		
					**Total ** **Sleep Time/** **Insomnia**	**Sleep ** **Quality**	**Vivid Dreams/** **Nightmares**	**Insomnia**	**Somnolence**	**Vivid Dreams/** **Nightmares**
**Randomised Controlled Trials**										
Önder *et al.*, 2006 [30]	12-week open-label randomised trial of fluoxetine, moclobemide, and tianeptine	N = 103 (fluo 38; mocl 35; tian 30)	Not allowed	Fluoxetine: (20-40 mg); Moclobemide (450-900 mg); Tianeptine (37.5-50 mg)	n.i.	n.i.	n.i.	= Fluo 2.6%; mocl 2.9%; tian 0%	= Fluo 0%; mocl 0%; tian 3.3%	n.i.
**Observational Studies**					*Compared to Baseline*					
Neal *et al.*, 1997 [163]	12-week prospective, open-label, pilot uncontrolled study	N = 20 civilians, victims, and veterans with PTSD (N = 18 completers)	Benzodiazepines (stable dose during the trial)	Moclobemide n.i. (300-600 mg)	n.i.	+	+	n.i.	n.i.	n.i.

**Abbreviations:** n.i. = no information, N = number of participants, mg = milligrams.

**Note:** For controlled trials: = no difference.

For observational studies: + improvement compared to baseline.

**Table 22:** Therapeutic and adverse effects of phenelzine on sleep-related outcomes in patients with PTSD.

**First ** **Author/** **Year**	**Study Design**	**Population**	**Concurrent Intermittent Rescue Sleep ** **Therapy**		**Dose (Range)**	**Outcomes**					
						**Efficacy**			**Safety**		
						**Total ** **Sleep ** **Time/** **Insomnia**	**Sleep ** **Quality**	**Vivid Dreams/** **Nightmares**	**Insomnia**	**Somnolence**	**Vivid Dreams/** **Nightmares**
**A. Randomised Controlled Trials**											
Shestatzky, *et al.*, 1987 [167]	12-week double-blind, randomised cross-over placebo-controlled clinical trial, 5 weeks of each treatment phase, monotherapy	N = 13 (phen 10; pbo 10)		Not allowed	Phenelzine: (45-75 mg)	n.i.	= it was hypothesized that phenelzine would be superior to placebo and would have a specific effect on sleep disturbance. These expectations were not fulfilled	=	n.i.	n.i.	n.i.
**B. Observational Studies**						*Compared to Baseline*					
Walker, 1982 [166]	Three case reports	N = 3		n.i.	Phenelzine: 60 mg	n.i.	n.i.	+ less frequent traumatic dreams and flashbacks	n.i.	n.i.	n.i.
Davidson *et al.*, 1987 [168]	6-week open prospective trial, monotherapy	N = 11 entered, 10 completed, 4 weeks		Not allowed	Phenelzine: 45 mg (45-60 mg)	n.i.	+ Improvement of sleep disturbance, 41%	+ 42% improvement	36.7% The aggravation of sleep disturbance was especially troublesome	9.1%	n.i.
Lerer *et al.*, 1987 [169]	At least 4-week duration, open prospective trial, monotherapy	N = 25, 22 completed at least 4 weeks of treatment		n.i.	Phenelzine: 60 mg (30-90 mg)	+ (35.7% improvement of initial insomnia, 27.3% improvement of maintenance insomnia, but no improvement of early morning awakening)	+ Sleep disturbance was the most severe symptom and was most consistently improved (in 36.4%)	+ Traumatic dreams (18.2%) were moderately reduced	n.i.	(3 drop-outs)	n.i.
Milanes *et al.*, 1984 [170]	8-week open trial	N = 10		n.i.	Phenelzine: 1 mg/kg	n.i.	n.i.	+ 60%	n.i.	n.i.	n.i.
Hogben *et al.*, 1981 [171]	Five case reports, not monotherapy	N = 5		Allowed	Phenelzine: (45-75 mg)	n.i.	n.i.	+ 100% cessation	n.i.	n.i.	n.i.
Levenson *et al.*, 1982 [172]	Case report	N = 1		n.i.	Phenelzine: 60 mg (15-75 mg)	n.i.	n.i.	+ the nightmares ceased	n.i.	n.i.	n.i.
Shen and Park, 1983 [173]	Case report	N = 1		n.i.	Phenelzine: 105 mg	n.i.	n.i.	+	n.i.	n.i.	n.i.

**Abbreviations:** n.i. = no information, N = number of participants, mg = milligrams.

**Note:** For controlled trials: = no difference.

For observational studies: + improvement compared to baseline.

**Table 23:** Therapeutic and adverse effects of bupropion on sleep-related outcomes in patients with PTSD.

**First ** **Author/** **Year**	**Study ** **Design**	**Population**	**Concurrent Intermittent Rescue Sleep Therapy**	**Dose ** **(Range)**	**Outcomes**					
					**Efficacy**			**Safety**		
					**Total ** **Sleep ** **Time/** **Insomnia**	**Sleep ** **Quality**	**Vivid ** **Dreams/** **Nightmares**	**Insomnia**	**Somnolence**	**Vivid Dreams/** **Nightmares**
**A. Randomised Controlled Trials**										
Becker *et al.*, 2007 [35]	8-week double-blind, randomised placebo-controlled clinical trial, not monotherapy, for smoking cessation	N = 30, analysis on 28 (bup 18; pbo 10)	Allowed	Bupropion 300 mg (100-300 mg)	n.i.	= Significant baseline to endpoint effect, but not a group effect	n.i.	n.i.	n.i.	n.i.
Hertzberg *et al.* 1999 [36]	12-week double-blind, randomised placebo-controlled clinical trial, not monotherapy, for smoking cessation	N = 15 (bup 10; pbo 5)	Allowed	Bupropion 300 mg (100-300 mg)	n.i.	= there were no significant changes in the scores for the PSQI, but no numerical data were provided	n.i.	n.i.	n.i.	n.i.
**B. Observational Studies**					*Compared to Baseline*					
Cañive *et al.*, 1998 [175]	6-week open prospective trial, monotherapy	N = 11 entered, 10 completed 4 weeks	Not allowed	Bupropion: 295 (200-400 mg)	n.i.	- “The aggravation of sleep disturbance was especially troublesome”	n.i.	“Five patients were prescribed chloral hydrate or lorazepam for bedtime sedation”	“Three discontinued bupropion treatment because of side effects, including loss of sleep, and sedation”	n.i.

**Abbreviations:** n.i. = no information, N = number of participants, mg = milligrams.

**Note:** For controlled trials: = no difference.

For observational studies: - deterioration compared to baseline.

**Table 24:** Therapeutic and adverse effects of vilazodone on sleep-related outcomes in patients with PTSD.

**First ** **Author/** **Year**	**Study Design**	**Population**	**Concurrent Intermittent Rescue Sleep Therapy**	**Dose ** **(Range)**	**Outcomes**					
					**Efficacy**			**Safety**		
					**Total Sleep Time/** **Insomnia**	**Sleep ** **Quality**	**Vivid ** **Dreams/** **Nightmares**	**Insomnia**	**Somnolence**	**Vivid Dreams/** **Nightmares**
**A. Randomised Controlled Trials**					*Vilazodone vs. Placebo*					
Ramaswamy *et al.*, 2017 [181]	12-week double-blind, randomised, placebo-controlled trial, monotherapy	N = 59 (vil 29; pbo 30)	Zolpidem for sleep	Vilazodone: n.i. (10-40 mg)	n.i.	= (CAPS-item 13; BDI-II-item 16; PSS-SR-item 13)	= (CAPS-item 2)	n.i.	n.i.	n.i.

**Abbreviations:** n.i. = no information, N = number of participants, mg = milligrams, CAPS = Clinician-Administered PTSD Scale, BDI-II = Beck Depression Inventory-II, PSS-SR = PTSD Symptom Scale-Self-Report. **Note:** For controlled trials: = no difference.

## LIST OF ABBREVIATIONS

BDI: Beck’s Depression Inventory
CAPS: Clinician-Administered PTSD Scale
CAPS-SX17: 17-item Clinician-Administered PTSD Scale
CBT: Cognitive Behavioural Therapy
DAT: Dopamine Transporter
DSM: Diagnostic and Statistical Manual of Mental Disorders
DTS: Davidson Trauma Scale
EMM: Enhanced Medication Management
ER: Extended Release
FDA: Food and Drug Administration
HDS: Hamilton Depression Scale
HSLEE*P*: Hours of Sleep
IES-R: Impact of Event Scale Revised
MADRS: Montgomery-Åsberg Depression Rating Scale
MAOI: Monoamine Oxidase Inhibitor
MD: Mean Differences
N: Number of Patients Included
NARD: Norepinephrine-Dopamine Reuptake Inhibitors (NARDs)
NaRI: Noradrenergic Reuptake Inhibitor
NaSSA: Noradrenergic and Specific Serotonergic Antidepressant
NET: Norepinephrine Transporter
NICE: National Institute for Health and Care Excellence
NNT: Number Needed to Treat
NREM: Non-rapid Eye Movement Sleep
*p*: *p*-value
PET: Prolonged Exposure Therapy
PSQI: Pittsburgh Sleep Quality Index
PSQI-A: Pittsburgh Sleep Quality Index-Addendum for PTSD
PTSD: Post Traumatic Stress Disorder
RCT: Randomised Controlled Trial
REM: Rapid Eye Movement Sleep
RR: Relative Risk
SARI: Serotonin Antagonist and Reuptake Inhibitors
SD: Standard Deviation
SERT: Serotonin Transporter
SI*P*: Structured Interview for PTSD
SMD: Standardised Mean Differences
SNRI: Serotonin-noradrenaline Reuptake Inhibitor
SPARI: Serotonin Partial Agonist Reuptake Inhibitor
SRRS: Stress Response Rating Scale
SSRI: Selective Serotonin Reuptake Inhibitor
TCA: Tricyclic Antidepressant
TES: Treatment-emergent Symptoms
UK: United Kingdom
US: United States of America

## References

[1] Goldstein R.B., Smith S.M., Chou S.P., Saha T.D., Jung J., Zhang H., Pickering R.P., Ruan W.J., Huang B., Grant B.F. (2016). The epidemiology of DSM-5 posttraumatic stress disorder in the United States: Results from the National Epidemiologic Survey on Alcohol and Related Conditions-III.. Soc. Psychiatry Psychiatr. Epidemiol..

[2] Murthy R.S., Christodoulou G.N., Mezzich J.E., Christodoulou N.G., Lecic-Tosevski D. (2016). Conflict situations and mental health care in developing countries.. Disasters: mental health context and responses 1st ed.

[3] de Jong J.T.V.M., Komproe I.H., Van Ommeren M., El Masri M., Araya M., Khaled N., van De Put W., Somasundaram D. (2001). Lifetime events and posttraumatic stress disorder in 4 postconflict settings.. JAMA.

[4] Kessler R.C., Sonnega A., Bromet E., Hughes M., Nelson C.B. (1995). Posttraumatic stress disorder in the national comorbidity survey.. Arch. Gen. Psychiatry.

[5] Kessler R.C., Chiu W.T., Demler O., Walters E.E., Walters E.E. (2005). Prevalence, severity, and comorbidity of 12-month DSM-IV disorders in the National Comorbidity Survey Replication.. Arch. Gen. Psychiatry.

[6] Ozer E.J., Best S.R., Lipsey T.L., Weiss D.S. (2003). Predictors of posttraumatic stress disorder and symptoms in adults: A meta-analysis.. Psychol. Bull..

[7] Solomon S.D., Davidson J.R. (1997). Trauma: prevalence, impairment, service use, and cost.. J. Clin. Psychiatry.

[8] McFARLANE (2010). A.C. The long-term costs of traumatic stress: intertwined physical and psychological consequences.. World Psychiatry.

[9] Qi W., Gevonden M., Shalev A. (2016). Prevention of post-traumatic stress disorder after trauma: current evidence and future directions.. Curr. Psychiatry Rep..

[10] Milanak M.E., Zuromski K.L., Cero I., Wilkerson A.K., Resnick H.S., Kilpatrick D.G. (2019). Traumatic event exposure, posttraumatic stress disorder, and sleep disturbances in a national sample of U.S. adults.. J. Trauma. Stress.

[11] Maguire D.G., Ruddock M.W., Milanak M.E., Moore T., Cobice D., Armour C. (2020). Sleep, a governor of morbidity in PTSD: a systematic review of biological markers in PTSD-related sleep disturbances.. Nat. Sci. Sleep.

[12] Cox R.C., Tuck B.M., Olatunji B.O. (2017). Sleep disturbance in posttraumatic stress disorder: epiphenomenon or causal factor?. Curr. Psychiatry Rep..

[13] Krakow B.J., Ulibarri V.A., Moore B.A., McIver N.D. (2015). Posttraumatic stress disorder and sleep-disordered breathing: a review of comorbidity research.. Sleep Med. Rev..

[14] Barden N., Reul J.M.H.M., Holsboer F. (1995). Do antidepressants stabilize mood through actions on the hypothalamic-pituitary-adrenocortical system?. Trends Neurosci..

[15] Nikisch G. (2009). Involvement and role of antidepressant drugs of the hypothalamic-pituitary-adrenal axis and glucocorticoid receptor function.. Neuroendocrinol. Lett..

[16] Barden N. (1996). Modulation of glucocorticoid receptor gene expression by antidepressant drugs.. Pharmacopsychiatry.

[17] Heydendael W., Jacobson L. (2010). Widespread hypothalamic-pituitary-adrenocortical axis-relevant and mood-relevant effects of chronic fluoxetine treatment on glucocorticoid receptor gene expression in mice.. Eur. J. Neurosci..

[18] De Crescenzo F., D’Alò G.L., Ostinelli E.G., Ciabattini M., Di Franco V., Watanabe N., Kurtulmus A., Tomlinson A., Mitrova Z., Foti F., Del Giovane C., Quested D.J., Cowen P.J., Barbui C., Amato L., Efthimiou O., Cipriani A. (2022). Comparative effects of pharmacological interventions for the acute and long-term management of insomnia disorder in adults: a systematic review and network meta-analysis.. Lancet.

[19] Samara M.T. (2022). What is the right drug for insomnia disorder?. Lancet.

[20] Ressler K.J. (2018). Alpha-Adrenergic Receptors in PTSD — Failure or time for precision medicine?. N. Engl. J. Med..

[21] Williams T., Phillips N.J., Stein D.J., Ipser J.C. (2022). Pharmacotherapy for post traumatic stress disorder (PTSD).. Cochrane Database Syst. Rev..

[22] DerSimonian R., Laird N. (1986). Meta-analysis in clinical trials.. Control. Clin. Trials.

[23] Deeks J.J., Higgins J.P.T., Altman D.J., Higgins J.P.T., Green S. (2008). Analyzing data and undertaking meta-analyses.. Cochrane handbook for systematic reviews of interventions 1st ed.

[24] Martenyi F., Brown E.B., Zhang H., Koke S.C., Prakash A. (2002). Fluoxetine v. placebo in prevention of relapse in post-traumatic stress disorder.. Br. J. Psychiatry.

[25] Davidson J.R.T., Connor K.M., Hertzberg M.A., Weisler R.H., Wilson W.H., Payne V.M. (2005). Maintenance therapy with fluoxetine in post-traumatic stress disorder: a placebo-controlled discontinuation study.. J. Clin. Psychopharmacol..

[26] (2000). A study of flexible dose brexpiprazole as monotherapy or combination therapy in the treatment of adults with post-traumatic stress disorder (PTSD).. ClinicalTrials.gov Identifier: NCT03033069.

[27] (2001). Attachment to FDA approval letter NDA 20-031/S-029. Food and Drug Administration (FDA).. https://www.accessdata.fda.gov/drugsatfda_docs/label/2001/20031s29lbl.pdf.

[28] Tucker P., Potter-Kimball R., Wyatt D.B., Parker D.E., Burgin C., Jones D.E., Masters B.K. (2003). Can physiologic assessment and side effects tease out differences in PTSD trials? A double-blind comparison of citalopram, sertraline, and placebo.. Psychopharmacol. Bull..

[29] Davidson J., Rothbaum B.O., Tucker P., Asnis G., Benattia I., Musgnung J.J. (2006). Venlafaxine extended release in posttraumatic stress disorder: a sertraline- and placebo-controlled study.. J. Clin. Psychopharmacol..

[30] Önder E., Tural Ü., Aker T. (2006). A comparative study of fluoxetine, moclobemide, and tianeptine in the treatment of posttraumatic stress disorder following an earthquake.. Eur. Psychiatry.

[31] Spivak B., Strous R.D., Shaked G., Shabash E., Kotler M., Weizman A. (2006). Reboxetine versus fluvoxamine in the treatment of motor vehicle accident-related posttraumatic stress disorder: a double-blind, fixed-dosage, controlled trial.. J. Clin. Psychopharmacol..

[32] Chung M.Y., Min K.H., Jun Y.J., Kim S.S., Kim W.C., Jun E.M. (2004). Efficacy and tolerability of mirtazapine and sertraline in Korean veterans with posttraumatic stress disorder: A randomized open label trial.. Hum. Psychopharmacol..

[33] McRae A.L., Brady K.T., Mellman T.A., Sonne S.C., Killeen T.K., Timmerman M.A., Bayles-Dazet W. (2004). Comparison of nefazodone and sertraline for the treatment of posttraumatic stress disorder.. Depress. Anxiety.

[34] Seo H.J., Jung Y.E., Bahk W.M., Jun T.Y., Chae J.H. (2010). A comparison of mirtazapine and paroxetine for the treatment of patients with posttraumatic stress disorder: a randomized open-label trial.. Clin. Psychopharmacol. Neurosci..

[35] Becker M.E., Hertzberg M.A., Moore S.D., Dennis M.F., Bukenya D.S., Beckham J.C. (2007). A placebo-controlled trial of bupropion SR in the treatment of chronic posttraumatic stress disorder.. J. Clin. Psychopharmacol..

[36] Hertzberg M.A., Moore S.D., Feldman M.E., Beckham J.C. (2001). A preliminary study of bupropion sustained-release for smoking cessation in patients with chronic posttraumatic stress disorder.. J. Clin. Psychopharmacol..

[37] Schneier F.R., Campeas R., Carcamo J., Glass A., Lewis-Fernandez R., Neria Y., Sanchez-Lacay A., Vermes D., Wall M.M. (2015). Combined mirtazapine and SSRI treatment of PTSD: a placebo-controlled trial.. Depress. Anxiety.

[38] Schneier F.R., Neria Y., Pavlicova M., Hembree E., Suh E.J., Amsel L., Marshall R.D. (2012). Combined prolonged exposure therapy and paroxetine for PTSD related to the World Trade Center attack: a randomized controlled trial.. Am. J. Psychiatry.

[39] Simon N.M., Connor K.M., Lang A.J., Rauch S., Krulewicz S., LeBeau R.T., Davidson J.R.T., Stein M.B., Otto M.W., Foa E.B., Pollack M.H. (2008). Paroxetine CR augmentation for posttraumatic stress disorder refractory to prolonged exposure therapy.. J. Clin. Psychiatry.

[40] Tripp J.C., Norman S.B., Kim H.M., Venners M.R., Martis B., Simon N.M., Stein M.B., Allard C.B. (2020). Rauch. S.A.M. PROGrESS study team. Residual symptoms of PTSD following Sertraline plus enhanced medication management, Sertraline plus PE, and PE plus placebo.. Psychiatry Res..

[41] (2000). Serotonin selective reuptake inhibitor treatment of dual diagnosis post-traumatic stress disorder and alcohol problems (DDx). ClinicalTrials. gov Identifier: NCT02504931.

[42] Trivedi M.H., Rush A.J., Wisniewski S.R., Nierenberg A.A., Warden D., Ritz L., Norquist G., Howland R.H., Lebowitz B., McGrath P.J., Shores-Wilson K., Biggs M.M., Balasubramani G.K., Fava M. (2006). Evaluation of outcomes with citalopram for depression using measurement-based care in STAR*D: implications for clinical practice.. Am. J. Psychiatry.

[43] Cipriani A., Furukawa T.A., Salanti G., Chaimani A., Atkinson L.Z., Ogawa Y., Leucht S., Ruhe H.G., Turner E.H., Higgins J.P.T., Egger M., Takeshima N., Hayasaka Y., Imai H., Shinohara K., Tajika A., Ioannidis J.P.A., Geddes J.R. (2018). Comparative efficacy and acceptability of 21 antidepressant drugs for the acute treatment of adults with major depressive disorder: a systematic review and network meta-analysis.. Lancet.

[44] Cipriani A., Williams T., Nikolakopoulou A., Salanti G., Chaimani A., Ipser J., Cowen P.J., Geddes J.R., Stein D.J. (2018). Comparative efficacy and acceptability of pharmacological treatments for post-traumatic stress disorder in adults: a network meta-analysis.. Psychol. Med..

[45] Hoskins M., Pearce J., Bethell A., Dankova L., Barbui C., Tol W.A., van Ommeren M., de Jong J., Seedat S., Chen H., Bisson J.I. (2015). Pharmacotherapy for post-traumatic stress disorder: Systematic review and meta-analysis.. Br. J. Psychiatry.

[46] English B.A., Jewell M., Jewell G., Ambrose S., Davis L.L. (2006). Treatment of chronic posttraumatic stress disorder in combat veterans with citalopram: an open trial.. J. Clin. Psychopharmacol..

[47] Seedat S., Stein D.J., Emsley R.A. (2000). Open trial of citalopram in adults with post-traumatic stress disorder.. Int. J. Neuropsychopharmacol..

[48] Khouzam H.R., El-Gabalawi F., Donnelly N.J. (2001). The clinical experience of citalopram in the treatment of post-traumatic stress disorder: a report of two Persian Gulf War veterans.. Mil. Med..

[49] Jakubovski E., Varigonda A.L., Freemantle N., Taylor M.J., Bloch M.H. (2016). Systematic review and meta-analysis: dose-response relationship of selective serotonin reuptake inhibitors in major depressive disorder.. Am. J. Psychiatry.

[50] Burke W.J. (2002). Escitalopram.. Expert Opin. Investig. Drugs.

[51] Funk K.A., Bostwick J.R. (2013). A comparison of the risk of QT prolongation among SSRIs.. Ann. Pharmacother..

[52] Nakatani Y., Amano T. (2021). Contributions of S- and R-citalopram to the citalopram-induced modulation of the function of Nav1.5 voltage-gated sodium channels.. Eur. J. Pharmacol..

[53] Pastoor D., Gobburu J. (2014). Clinical pharmacology review of escitalopram for the treatment of depression.. Expert Opin. Drug Metab. Toxicol..

[54] Robert S., Hamner M.B., Ulmer H.G., Lorberbaum J.P., Durkalsk V.L. (2006). Open-label trial of escitalopram in the treatment of posttraumatic stress disorder.. J. Clin. Psychiatry.

[55] Ramaswamy S., Selvaraj V., Driscoll D., Madabushi J.S., Bhatia S.C., Yeragani V. (2015). Effects of escitalopram on autonomic function in posttraumatic stress disorder among veterans of operations enduring freedom and iraqi freedom (OEF/OIF).. Innov. Clin. Neurosci..

[56] Qi W., Gevonden M., Shalev A. (2017). Efficacy and tolerability of high-dose escitalopram in posttraumatic stress disorder.. J. Clin. Psychopharmacol..

[57] Magni L.R., Purgato M., Gastaldon C., Papola D., Furukawa T.A., Cipriani A., Barbui C. (2013). Fluoxetine versus other types of pharmacotherapy for depression.. Cochrane Libr..

[58] Connor K.M., Sutherland S.M., Tupler L.A., Malik M.L., Jonathan R., Davidson T. (1999). Fluoxetine in post-traumatic stress disorder.. Br. J. Psychiatry.

[59] Cohen H., Kotler M., Matar M., Kaplan Z. (2000). Normalization of heart rate variability in post-traumatic stress disorder patients following fluoxetine treatment: preliminary results.. Isr. Med. Assoc. J..

[60] Hertzberg M., Feldman M., Beckham J., Kudler H., Davidson J. (2000). Lack of efficacy for fluoxetine in PTSD: a placebo controlled trial in combat veterans.. Ann. Clin. Psychiatry.

[61] Fernandez M., Pissiota A., Frans Ö., von Knorring L., Fischer H., Fredrikson M. (2001). Brain function in a patient with torture related post-traumatic stress disorder before and after fluoxetine treatment: a positron emission tomography provocation study.. Neurosci. Lett..

[62] Barnett S.D., Tharwani H.M., Hertzberg M.A., Sutherland S.M., Connor K.M., Davidson J.R.T. (2002). Tolerability of fluoxetine in posttraumatic stress disorder.. Prog. Neuropsychopharmacol. Biol. Psychiatry.

[63] Martenyi F., Brown E.B., Zhang H., Prakash A., Koke S.C. (2002). Fluoxetine versus placebo in posttraumatic stress disorder.. J. Clin. Psychiatry.

[64] van der Kolk B.A., Spinazzola J., Blaustein M.E. (2007). Eye movement desensitisation and reprocessing reduces PTSD symptoms compared with fluoxetine at six months post-treatment.. J. Clin. Psychiatry.

[65] McDougle C.J., Southwick S.M., Charney D.S., St James R.L. (1991). An open trial of fluoxetine in the treatment of posttraumatic stress disorder.. J. Clin. Psychopharmacol..

[66] Davidson J., Roth S., Newman E. (1991). Fluoxetine in post-traumatic stress disorder.. J. Trauma. Stress.

[67] Shay J. (1992). Fluoxetine reduces explosiveness and elevates mood of vietnam combat vets with PTSD.. J. Trauma. Stress.

[68] March J.S. (1992). Fluoxetine and fluvoxamine in PTSD.. Am. J. Psychiatry.

[69] Nagy L.M., Morgan C.A., Southwick S.M., Charney D.S. (1993). Open prospective trial of fluoxetine for posttraumatic stress disorder.. J. Clin. Psychopharmacol..

[70] van der Kolk B.A., Dreyfuss D., Michaels M., Shera D., Berkowitz R., Fisler R., Saxe G. (1994). Fluoxetine in posttraumatic stress disorder.. J. Clin. Psychiatry.

[71] Folnegović-Šmalc V., Folnegović Z., Henigsberg N., Jernej B., Makarić G., Mimica N. (1997). Efficacy of fluoxetine in PTSD patients.. Abstr 6th World Congr Biol Psychiatry.

[72] Meltzer-Brody S., Connor K.M., Churchill E., Davidson J.R.T. (2000). Symptom-specific effects of fluoxetine in post-traumatic stress disorder.. Int. Clin. Psychopharmacol..

[73] Martenyi F., Brown E.B., Caldwell C.D. (2007). Failed efficacy of fluoxetine in the treatment of posttraumatic stress disorder: results of a fixed-dose, placebo-controlled study.. J. Clin. Psychopharmacol..

[74] De Boer M., Op den Velde W., Falger P.J.R., Hovens J.E., De Groen J.H.M., Van Duijn H. (1992). Fluvoxamine treatment for chronic PTSD: a pilot study.. Psychother. Psychosom..

[75] Davidson J.R.T., Weisler R.H., Malik M., Tupler L.A. (1998). Fluvoxamine in civilians with posttraumatic stress disorder.. J. Clin. Psychopharmacol..

[76] Neylan T.C., Metzler T.J., Schoenfeld F.B., Weiss D.S., Lenoci M., Best S.R., Lipsey T.L., Marmar C.R. (2001). Fluvoxamine and sleep disturbances in posttraumatic stress disorder.. J. Trauma. Stress.

[77] Marmar C.R., Schoenfeld F., Weiss D.S., Metzler T., Zatzick D., Wu R., Smiga S., Tecott L., Neylan T. (1996). Open trial of fluvoxamine treatment for combat-related posttraumatic stress disorder.. J. Clin. Psychiatry.

[78] Escalona R., Canive J.M., Calais L.A., Davidson J.R.T. (2002). Fluvoxamine treatment in veterans with combat-related post-traumatic stress disorder.. Depress. Anxiety.

[79] Schoenfeld F.B., DeViva J.C., Manber R. (2012). Treatment of sleep disturbances in posttraumatic stress disorder: A review.. J. Rehabil. Res. Dev..

[80] (2006). Reboxetine an effective alternative for PTSD.. Inpharma Wkly.

[81] (2018). Post-traumatic stress disorder: NICE guideline [NG116]. National Institute for Health and Care Excellence (NICE).. https://www.nice.org.uk/guidance/ng116/resources/posttraumaticstress-disorder-pdf-66141601777861.

[82] Marshall R.D., Beebe K.L., Oldham M., Zaninelli R. (2001). Efficacy and safety of paroxetine treatment for chronic PTSD: a fixed-dose, placebo-controlled study.. Am. J. Psychiatry.

[83] Tucker P., Zaninelli R., Yehuda R., Ruggiero L., Dillingham K., Pitts C.D. (2001). Paroxetine in the treatment of chronic posttraumatic stress disorder: results of a placebo-controlled, flexible-dosage trial.. J. Clin. Psychiatry.

[84] Stein D.J., Davidson J., Seedat S., Beebe K. (2003). Paroxetine in the treatment of post-traumatic stress disorder: pooled analysis of placebo-controlled studies.. Expert Opin. Pharmacother..

[85] Sheehan D., Beebe K.L., Dube E.M. (2002). Paroxetine for the treatment of sleep disturbance in posttraumatic stress disorder.. Eur. Neuropsychopharmacol..

[86] de Moraes Costa G., Ziegelmann P.K., Zanatta F.B., Martins C.C., de Moraes Costa P., Mello C.F. (2022). Efficacy, acceptability, and tolerability of antidepressants for sleep quality disturbances in post-traumatic stress disorder: A systematic review and network meta-analysis.. Prog. Neuropsychopharmacol. Biol. Psychiatry.

[87] Prazosin vs (2000). paroxetine in combat stress-related post-traumatic stress disorder (PTSD) nightmares & sleep disturbance.. ClinicalTrials.gov Identifier: NCT00202449.

[88] Combination Treatment for Posttraumatic Stress Disorder (PTSD) After the World Trade Center (WTC) Attack (2000). ClinicalTrials.gov Identifier: NCT01130103.

[89] Tucker P., Beebe K.L., Burgin C., Wyatt D.B., Parker D.E., Masters B.K., Nawar O. (2004). Paroxetine treatment of depression with posttraumatic stress disorder: effects on autonomic reactivity and cortisol secretion.. J. Clin. Psychopharmacol..

[90] O’Connor C.M., Jiang W., Kuchibhatla M., Silva S.G., Cuffe M.S., Callwood D.D., Zakhary B., Stough W.G., Arias R.M., Rivelli S.K., Krishnan R. (2010). Safety and efficacy of sertraline for depression in patients with heart failure: results of the SADHARTCHF (Sertraline Against Depression and Heart Disease in Chronic Heart Failure) trial.. J. Am. Coll. Cardiol..

[91] Alexander W. (2012). Pharmacotherapy for post-traumatic stress disorder in combat veterans: focus on antidepressants and atypical antipsychotic agents.. P&T.

[92] Sharpley A.L., Cowen P.J. (1995). Effect of pharmacologic treatments on the sleep of depressed patients.. Biol. Psychiatry.

[93] Mellman T.A., Pigeon W.R., Nowell P.D., Nolan B. (2007). Relationships between REM sleep findings and PTSD symptoms during the early aftermath of trauma.. J. Trauma. Stress.

[94] Brady K., Pearlstein T., Asnis G.M., Baker D., Rothbaum B., Sikes C.R., Farfel G.M. (2000). Efficacy and safety of sertraline treatment of posttraumatic stress disorder: a randomized controlled trial.. JAMA.

[95] Davidson J.R.T., Rothbaum B.O., van der Kolk B.A., Sikes C.R., Farfel G.M. (2001). Multicenter, double-blind comparison of sertraline and placebo in the treatment of posttraumatic stress disorder.. Arch. Gen. Psychiatry.

[96] Davidson J.R.T., Landerman L.R., Farfel G.M., Clary C.M. (2002). Characterizing the effects of sertraline in post-traumatic stress disorder.. Psychol. Med..

[97] Friedman M.J., Marmar C.R., Baker D.G., Sikes C.R., Farfel G.M. (2007). Randomized, double-blind comparison of sertraline and placebo for posttraumatic stress disorder in a Department of Veterans Affairs setting.. J. Clin. Psychiatry.

[98] Davidson J., Baldwin D., Stein D.J., Kuper E., Benattia I., Ahmed S., Pedersen R., Musgnung J. (2006). Treatment of posttraumatic stress disorder with venlafaxine extended release: a 6-month randomized controlled trial.. Arch. Gen. Psychiatry.

[99] Li W., Ma Y.B., Yang Q., Li B., Meng Q.G., Zhang Y. (2017). Effect and safety of sertraline for treat posttraumatic stress disorder: a multicenter randomised controlled study.. Int. J. Psychiatry Clin. Pract..

[100] Panahi Y., Moghaddam B.R., Sahebkar A., Nazari M.A., Beiraghdar F., Karami G., Saadat A.R. (2011). A randomized, double-blind, placebo-controlled trial on the efficacy and tolerability of sertraline in Iranian veterans with post-traumatic stress disorder.. Psychol. Med..

[101] Zohar J., Amital D., Miodownik C., Kotler M., Bleich A., Lane R.M., Austin C. (2002). Double-blind placebo-controlled pilot study of sertraline in military veterans with posttraumatic stress disorder.. J. Clin. Psychopharmacol..

[102] Robb A.S., Cueva J.E., Sporn J., Yang R., Vanderburg D.G. (2010). Sertraline treatment of children and adolescents with posttraumatic stress disorder: a double-blind, placebo-controlled trial.. J. Child Adolesc. Psychopharmacol..

[103] Kamo T., Maeda M., Oe M., Kato H., Shigemura J., Kuribayashi K., Hoshino Y. (2016). Dosage, effectiveness, and safety of sertraline treatment for posttraumatic stress disorder in a Japanese clinical setting: a retrospective study.. BMC Psychiatry.

[104] Detke M.J., Wiltse C.G., Mallinckrodt C.H., McNamara R.K., Demitrack M.A., Bitter I. (2004). Duloxetine in the acute and long-term treatment of major depressive disorder: a placebo- and paroxetine-controlled trial.. Eur. Neuropsychopharmacol..

[105] Walderhaug E., Kasserman S., Aikins D., Vojvoda D., Nishimura C., Neumeister A. (2010). Effects of duloxetine in treatment-refractory men with posttraumatic stress disorder.. Pharmacopsychiatry.

[106] Villarreal G., Cañive J.M., Calais L.A., Toney G., Smith A.K. (2010). Duloxetine in military posttraumatic stress disorder.. Psychopharmacol. Bull..

[107] Deneys M.L., Ahearn E.P. (2006). Exacerbation of PTSD symptoms with use of duloxetine.. J. Clin. Psychiatry.

[108] Hanretta A.T., Malek-Ahmadi P. (2006). Combined use of ECT with duloxetine and olanzapine: a case report.. J. ECT.

[109] Coutens B., Yrondi A., Rampon C., Guiard B.P. (2022). Psychopharmacological properties and therapeutic profile of the antidepressant venlafaxine.. Psychopharmacology (Berl.).

[110] Stein D.J., Pedersen R., Rothbaum B.O., Baldwin D.S., Ahmed S., Musgnung J., Davidson J. (2009). Onset of activity and time to response on individual CAPS-SX17 items in patients treated for post-traumatic stress disorder with venlafaxine ER: a pooled analysis.. Int. J. Neuropsychopharmacol..

[111] Larrey D., Ripault M.P., Kaplowitz N., DeLeve L.D. (2013). Hepatotoxicity of psychotropic drugs and drugs of abuse.. Drug-induced liver disease, 3rd ed;.

[112] Spigset O., Hägg S., Bate A. (2003). Hepatic injury and pancreatitis during treatment with serotonin reuptake inhibitors.. Int. Clin. Psychopharmacol..

[113] Hidalgo R., Hertzberg M.A., Mellman T., Petty F., Tucker P., Weisler R., Zisook S., Chen S., Churchill E., Davidson J. (1999). Nefazodone in post-traumatic stress disorder: results from six open-label trials.. Int. Clin. Psychopharmacol..

[114] Cohn C.K., Robinson D.S., Roberts D.L., Schwiderski U.E., O’Brien K., Ieni J.R. (1996). Responders to antidepressant drug treatment: a study comparing nefazodone, imipramine, and placebo in patients with major depression.. J. Clin. Psychiatry.

[115] Armitage R., Yonkers K., Cole D., Rush A.J. (1997). A multicenter, double-blind comparison of the effects of nefazodone and fluoxetine on sleep architecture and quality of sleep in depressed outpatients.. J. Clin. Psychopharmacol..

[116] Davidson J.R.T., Weisler R.H., Malik M.L., Connor K.M. (1998). Treatment of posttraumatic stress disorder with nefazodone.. Int. Clin. Psychopharmacol..

[117] Gillin J.C., Smith-Vaniz A., Schnierow B., Rapaport M.H., Kelsoe J., Raimo E., Marler M.R., Goyette L.M., Stein M.B., Zisook S. (2001). An open-label, 12-week clinical and sleep EEG study of nefazodone in chronic combat-related posttraumatic stress disorder.. J. Clin. Psychiatry.

[118] Hertzberg M.A., Feldman M.E., Beckham J.C., Moore S.D., Davidson J.R.T. (1998). Open trial of nefazodone for combat-related posttraumatic stress disorder.. J. Clin. Psychiatry.

[119] Hertzberg M., Feldman M., Beckham J., Moore S., Davidson J. (2002). Three- to four-year follow-up to an open trial of nefazodone for combat-related posttraumatic stress disorder.. Ann. Clin. Psychiatry.

[120] Mellman T.A., David D., Barza L. (1999). Nefazodone treatment and dream reports in chronic PTSD.. Depress. Anxiety.

[121] Neylan T.C., Lenoci M., Maglione M.L., Rosenlicht N.Z., Leykin Y., Metzler T.J., Schoenfeld F.B., Marmar C.R. (2003). The effect of nefazodone on subjective and objective sleep quality in posttraumatic stress disorder.. J. Clin. Psychiatry.

[122] Zisook S., Chentsova-Dutton Y.E., Smith-Vaniz A., Kline N.A., Ellenor G.L., Kodsi A.B., Gillin J.C. (2000). Nefazodone in patients with treatment-refractory posttraumatic stress disorder.. J. Clin. Psychiatry.

[123] Montalbano A., Mlinar B., Bonfiglio F., Polenzani L., Magnani M., Corradetti R. (2019). Dual inhibitory action of trazodone on dorsal raphe serotonergic neurons through 5-HT1A receptor partial agonism and α1-adrenoceptor antagonism.. PLoS One.

[124] Atkin T., Comai S., Gobbi G. (2018). Drugs for insomnia beyond benzodiazepines: pharmacology, clinical applications, and discovery.. Pharmacol. Rev..

[125] Wichniak A., Wierzbicka A., Jarema M. (2021). Treatment of insomnia – effect of trazodone and hypnotics on sleep.. Psychiatr. Pol..

[126] Ashford J.W., Miller T.W. (1996). Effects of trazodone on sleep in patients diagnosed with post-traumatic stress disorder (PTSD).. J. Contemp. Psychother..

[127] Hertzberg M.A., Feldman M.E., Beckham J.C., Davidson J.R.T. (1996). Trial of trazodone for posttraumatic stress disorder using a multiple baseline group design.. J. Clin. Psychopharmacol..

[128] Warner M.D., Dorn M.R., Peabody C.A. (2001). Survey on the usefulness of trazodone in patients with PTSD with insomnia or nightmares.. Pharmacopsychiatry.

[129] Sepede G., Corbo M., Fiori F., Martinotti G. (2012). Reboxetine in clinical practice: a review.. Clin. Ter..

[130] McClure E.W., Daniels R.N. (2021). Classics in chemical neuroscience: amitriptyline.. ACS Chem. Neurosci..

[131] Everitt H., McDermott L., Leydon G., Yules H., Baldwin D., Little P. (2014). GPs’ management strategies for patients with insomnia: a survey and qualitative interview study.. Br. J. Gen. Pract..

[132] Everitt H., Baldwin D.S., Stuart B., Lipinska G., Mayers A., Malizia A.L., Manson C.C., Wilson S. (2018). Antidepressants for insomnia in adults.. Cochrane Database Syst. Rev..

[133] Davidson J., Kudler H., Smith R., Mahorney S.L., Lipper S., Hammett E., Saunders W.B., Cavenar J.O. (1990). Treatment of posttraumatic stress disorder with amitriptyline and placebo.. Arch. Gen. Psychiatry.

[134] Davidson J.R.T., Kudler H.S., Saunders W.B., Erickson L., Smith R.D., Stein R.M., Lipper S., Hammett E.B., Mahorney S.L., Cavenar J.O. (1993). Jr Predicting response to amitriptyline in posttraumatic stress disorder.. Am. J. Psychiatry.

[135] Falcon S., Ryan C., Chamberlain K., Curtis G. (1985). Tricyclics: possible treatment for posttraumatic stress disorder.. J. Clin. Psychiatry.

[136] Bleich A., Siegel B., Garb R., Lerer B. (1986). Post-traumatic stress disorder following combat exposure: clinical features and psychopharmacological treatment.. Br. J. Psychiatry.

[137] Başoǧlu M., Marks I.M., Sengün S. (1992). Amitriptyline for PTSD in a torture survivor: a case study.. J. Trauma. Stress.

[138] Gillman P.K. (2007). Tricyclic antidepressant pharmacology and therapeutic drug interactions updated.. Br. J. Pharmacol..

[139] Chen C.J. (1991). The obsessive quality and clomipramine treatment in PTSD.. Am. J. Psychiatry.

[140] Maan J.S., Rosani A., Saadabadi A. (2022). Desipramine.. StatPearls..

[141] Shimamura T., Shiroishi M., Weyand S., Tsujimoto H., Winter G., Katritch V., Abagyan R., Cherezov V., Liu W., Han G.W., Kobayashi T., Stevens R.C., Iwata S. (2011). Structure of the human histamine H1 receptor complex with doxepin.. Nature.

[142] Boehnlein J.K., Kinzie J.D., Ben R., Fleck J. (1985). One-year follow-up study of posttraumatic stress disorder among survivors of Cambodian concentration camps.. Am. J. Psychiatry.

[143] White N.S. (1983). Posttraumatic stress disorder.. Hosp. Community Psychiatry.

[144] Burstein A., Burstein A. (1983). Treatment of night terrors with imipramine.. J. Clin. Psychiatry.

[145] Burstein A. (1984). Treatment of post-traumatic stress disorder with imipramine.. Psychosomatics.

[146] Kinzie J.D., Leung P. (1989). Clonidine in Cambodian patients with posttraumatic stress disorder.. J. Nerv. Ment. Dis..

[147] Alamo C., García-Garcia P., Lopez-Muñoz F., Zaragozá C. (2019). Tianeptine, an atypical pharmacological approach to depression.. Rev. Psiquiatr y Salud Ment..

[148] Gassaway M.M., Rives M-L., Kruegel A.C., Javitch J.A., Sames D. (2014). The atypical antidepressant and neurorestorative agent tianeptine is a μ-opioid receptor agonist.. Transl. Psychiatry.

[149] Svenningsson P., Bateup H., Qi H., Takamiya K., Huganir R.L., Spedding M., Roth B.L., McEwen B.S., Greengard P. (2007). Involvement of AMPA receptor phosphorylation in antidepressant actions with special reference to tianeptine.. Eur. J. Neurosci..

[150] Wilde M.I., Benfield P. (1995). Tianeptine.. Drugs.

[151] Vuković O., Marić N.P., Britvić D., Cvetić T., Damjanović A., Prostran M., Jasović-Gasić M. (2009). Efficacy, tolerability and safety of tianeptine in special populations of depressive patients.. Psychiatr. Danub..

[152] Jilani T.N., Gibbons J.R., Faizy R.M. (2022). Mirtazapine.. StatPearls..

[153] Lewis J.D. (2002). Mirtazapine for PTSD Nightmares.. Am. J. Psychiatry.

[154] Davis L.L., Pilkinton P., Lin C., Parker P., Estes S., Bartolucci A. (2020). A randomized, placebo-controlled trial of mirtazapine for the treatment of posttraumatic stress disorder in veterans.. J. Clin. Psychiatry.

[155] Davidson J.R.T., Weisler R.H., Butterfield M.I., Casat C.D., Connor K.M., Barnett S., van Meter S. (2003). Mirtazapine vs. placebo in post-traumatic stress disorder: a pilot trial.. Biol. Psychiatry.

[156] Connor K.M., Davidson J.R.T., Weisler R.H., Ahearn E. (1999). A pilot study of mirtazapine in post-traumatic stress disorder.. Int. Clin. Psychopharmacol..

[157] Lotufo-Neto F., Trivedi M., Thase M.E. (1999). Meta-analysis of the reversible inhibitors of monoamine oxidase type A moclobemide and brofaromine for the treatment of depression.. Neuropsychopharmacology.

[158] Suchting R., Tirumalaraju V., Gareeb R., Bockmann T., de Dios C., Aickareth J., Pinjari O., Soares J.C., Cowen P.J., Selvaraj S. (2021). Revisiting monoamine oxidase inhibitors for the treatment of depressive disorders: A systematic review and network meta-analysis.. J. Affect. Disord..

[159] Katz R.J., Lott M.H., Arbus P., Crocq L., Herlobsen P., Lingjaerde O., Lopez G., Loughrey G.C., Macfarlane D.J., McIvor R., Mehlum L., Nugent D., Turner S.W., Weisaeth L., Yule W. (1994-1995). Pharmacotherapy of post-traumatic stress disorder with a novel psychotropic.. Anxiety.

[160] Baker D.G., Diamond B.I., Gillette G., Hamner M., Katzelnick D., Keller T., Mellman T.A., Pontius E., Rosenthal M., Tucker P. (1995). vander Kolk, B.A.; Katz, R. A double-blind, randomized, placebo-controlled, multi-center study of brofaromine in the treatment of post-traumatic stress disorder.. Psychopharmacology.

[161] Sub Laban T., Saadabadi A. (2022). Monoamine oxidase inhibitors (MAOI).. StatPearls..

[162] Thase M.E. (2012). MAOIs and depression treatment guidelines.. J. Clin. Psychiatry.

[163] Neal L.A., Shapland W., Fox C. (1997). An open trial of moclobemide in the treatment of post-traumatic stress disorder.. Int. Clin. Psychopharmacol..

[164] Sidhu G., Marwaha R. (2022). Phenelzine.. StatPearls..

[165] Chamberlain S.R., Baldwin D.S. (2021). Monoamine oxidase inhibitors (MAOIs) in psychiatric practice: how to use them safely and effectively.. CNS Drugs.

[166] Walker J.I. (1982). Chemotherapy of traumatic war stress.. Mil. Med..

[167] Shestatzky M., Greenberg D., Lerer B. (1988). A controlled trial of phenelzine in posttraumatic stress disorder.. Psychiatry Res..

[168] Davidson J., Walker J.I., Kilts C. (1987). A pilot study of phenelzine in the treatment of post-traumatic stress disorder.. Br. J. Psychiatry.

[169] Lerer B., Bleich A., Kotler M., Garb R., Hertzberg M., Levin B. (1987). Posttraumatic stress disorder in Israeli combat veterans. Effect of phenelzine treatment.. Arch. Gen. Psychiatry.

[170] Milanes F.J., Mack C.N., Dennison J., Slater V.L. (1984). Phenelzine treatment of post-Vietnam stress syndome.. VA Pract..

[171] Hogben G.L., Cornfield R.B. (1981). Treatment of traumatic war neurosis with phenelzine.. Arch. Gen. Psychiatry.

[172] Lanman R., Lanman R., Rankin M. (1982). Traumatic war neurosis and phenelzine.. Arch. Gen. Psychiatry.

[173] Shen W.W., Park S. (1983). The use of monoamine oxidase inhibitors in the treatment of traumatic war neurosis: case report.. Mil. Med..

[174] Pandhare A., Pappu A.S., Wilms H., Blanton M.P., Jansen M. (2017). The antidepressant bupropion is a negative allosteric modulator of serotonin type 3A receptors.. Neuropharmacology.

[175] Cañive J.M., Clark R.D., Calais L.A., Qualls C., Tuason V.B. (1998). Bupropion treatment in veterans with posttraumatic stress disorder: an open study.. J. Clin. Psychopharmacol..

[176] Kojima G., Tamai A., Karino S., Yuasa M., Epure J., Tsuzaki B., Tanabe M. (2013). Bupropion-related visual hallucinations in a veteran with posttraumatic stress disorder and multiple sclerosis.. J. Clin. Psychopharmacol..

[177] Dagan Y., Yager J. (2018). Severe bupropion XR abuse in a patient with long‐standing bulimia nervosa and complex PTSD.. Int. J. Eat. Disord..

[178] Wang S.M., Han C., Lee S.J., Patkar A.A., Masand P.S., Pae C.U. (2016). Vilazodone for the treatment of depression: an update.. Chonnam Med. J..

[179] Laughren T.P., Gobburu J., Temple R.J., Unger E.F., Bhattaram A., Dinh P.V., Fossom L., Hung H.M.J., Klimek V., Lee J.E., Levin R.L., Lindberg C.Y., Mathis M., Rosloff B.N., Wang S.J., Wang Y., Yang P., Yu B., Zhang H., Zhang L., Zineh I. (2011). Vilazodone.. J. Clin. Psychiatry.

[180] (2000). Vilazodone for the treatment of posttraumatic stress disorder.. ClinicalTrials. gov Identifier: NCT01715519.

[181] Ramaswamy S, Driscoll D, Reist C (2017). A double-blind, placebo-controlled randomized trial of vilazodone in the treatment of posttraumatic stress disorder and comorbid depression.. Prim Care Companion CNS Disord..

[182] (2000). Vortioxetine for posttraumatic stress disorder. ClinicalTrials.gov. Identifier: NCT02637895.

[183] Dunlop B.W., Rakofsky J.J., Newport D.J., Mletzko-Crowe T., Barone K., Nemeroff C.B., Harvey P.D. (2021). Efficacy of vortioxetine monotherapy for posttraumatic stress disorder: a randomized, placebo-controlled Trial.. J. Clin. Psychopharmacol..

[184] Durand D., Calcagno T.M., Wong A., Newport D.J., Nemeroff C.B., Dunlop B.W., Harvey P.D. (2021). Effects of vortioxetine versus placebo on cognition and functional capacity in adults with posttraumatic stress disorder.. J. Clin. Psychopharmacol..

[185] Imel Z.E., Laska K., Jakupcak M., Simpson T.L. (2013). Meta-analysis of dropout in treatments for posttraumatic stress disorder.. J. Consult. Clin. Psychol..

[186] Abdallah C.G., Averill L.A., Akiki T.J., Raza M., Averill C.L., Gomaa H., Adikey A., Krystal J.H. (2019). The neurobiology and pharmacotherapy of posttraumatic stress disorder.. Annu. Rev. Pharmacol. Toxicol..

[187] Quinones M.M., Gallegos A.M., Lin F.V., Heffner K. (2020). Dysregulation of inflammation, neurobiology, and cognitive function in PTSD: an integrative review.. Cogn. Affect. Behav. Neurosci..

[188] Baird T., Theal R., Gleeson S., McLeay S., O’Sullivan R., McLeay S., Harvey W., Romaniuk M., Crawford D., Colquhoun D., McD Young R. (2018). Dwyer, M.; Gibson, J.; O’Sullivan, R.; Cooksley, G.; Strakosch, C.; Thomson, R.; Voisey, J.; Lawford, B. Detailed poly-somnography in Australian Vietnam veterans with and without posttraumatic stress disorder.. J. Clin. Sleep Med..

[189] Brownlow J.A., Miller K.E., Gehrman P.R. (2020). Treatment of sleep comorbidities in posttraumatic stress disorder.. Curr. Treat. Options Psychiatry.

[190] Lancel M., van Marle H.J.F., Van Veen M.M., van Schagen A.M. (2021). Disturbed sleep in PTSD: thinking beyond nightmares.. Front. Psychiatry.

[191] Zhang Y., Ren R., Sanford L.D., Yang L., Ni Y., Zhou J., Zhang J., Wing Y.K., Shi J., Lu L., Tang X. (2020). The effects of prazosin on sleep disturbances in post-traumatic stress disorder: a systematic review and meta-analysis.. Sleep Med..

[192] Germain A., Hall M., Krakow B., Katherine Shear M., Buysse D.J. (2005). A brief sleep scale for posttraumatic stress disorder: Pittsburgh sleep quality index addendum for PTSD.. J. Anxiety Disord..

[193] Samara M.T., Huhn M., Chiocchia V., Schneider-Thoma J., Wiegand M., Salanti G., Leucht S. (2020). Efficacy, acceptability, and tolerability of all available treatments for insomnia in the elderly: a systematic review and network meta‐analysis.. Acta Psychiatr. Scand..

[194] Tribl G.G., Wetter T.C., Schredl M. (2013). Dreaming under antidepressants: A systematic review on evidence in depressive patients and healthy volunteers.. Sleep Med. Rev..

[195] Leucht S., Leucht C., Huhn M., Chaimani A., Mavridis D., Helfer B., Samara M., Rabaioli M., Bächer S., Cipriani A., Geddes J.R., Salanti G., Davis J.M. (2017). Sixty years of placebo-controlled antipsychotic drug trials in acute schizophrenia: systematic review, bayesian meta-analysis, and meta-regression of efficacy predictors.. Am. J. Psychiatry.

[196] Gieselmann A., Ait Aoudia M., Carr M., Germain A., Gorzka R., Holzinger B., Kleim B., Krakow B., Kunze A.E., Lancee J., Nadorff M.R., Nielsen T., Riemann D., Sandahl H., Schlarb A.A., Schmid C., Schredl M., Spoormaker V.I., Steil R., van Schagen A.M., Wittmann L., Zschoche M., Pietrowsky R. (2019). Aetiology and treatment of nightmare disorder: State of the art and future perspectives.. J. Sleep Res..

[197] Marken P.A., Munro J.S. (2000). Selecting a selective serotonin reuptake inhibitor: clinically important distinguishing features.. Prim. Care Companion J. Clin. Psychiatry.

[198] Murata Y., Kamishioiri Y., Tanaka K., Sugimoto H., Sakamoto S., Kobayashi D., Mine K. (2013). Severe sleepiness and excess sleep duration induced by paroxetine treatment is a beneficial pharmacological effect, not an adverse reaction.. J. Affect. Disord..

[199] Zhang B., Wang C., Cui L., Gao J., Wang C., Tan X., Fang S. (2020). Short-term efficacy and tolerability of paroxetine versus placebo for panic disorder: a meta-analysis of randomized controlled trials.. Front. Pharmacol..

[200] Nevels R.M., Gontkovsky S.T., Williams B.E. (2016). Paroxetine—the antidepressant from hell? Probably not, but caution required.. Psychopharmacol. Bull..

