# Supplementary material for: Effects of Antidepressants on Sleep in Post-traumatic Stress Disorder: An Overview of Reviews
Source: Curr Neuropharmacol. 2023 Aug 15;22(4):749–805. doi: 10.2174/1570159X21666230801144328 (PMC10845105; doi:10.2174/1570159X21666230801144328)
Supplement: Supplementary file 1 — Supplementary material is available on the publisher’s website along with the published article. [file CN-22-749_SD1.pdf]

Supplementary Material

Effects of Antidepressants on Sleep in Post-traumatic Stress Disorder: An Overview of Reviews

Andreas S. Lappas[1,2], Zoi A. Polyzopoulou[3], Nikos Christodoulou[1,4], Vasilios-Panteleimon Bozikas[5] and Myrto T. Samara[1,*]

[1]Department of Psychiatry, Medical School, General University Hospital of Larissa, University of Thessaly, Larissa, Greece; [2]Department of Geriatric Liaison Psychiatry, Royal Gwent Hospital, Newport, United Kingdom; [3]Department of Psychology, University of Western Macedonia, Florina, 53100, Greece; [4]School of Medicine, University of Nottingham, Nottingham, England, United Kingdom; [5]II Department of Psychiatry, School of Medicine, Aristotle University of Thessaloniki, Lagkada Str. 196, 56430 Thessaloniki, Greece

1875-6190/24 $65.00+.00 **© 2024 Bentham Science Publishers**

Supplement 1: Studies awaiting assessment

AMITRIPTYLINE - IMIPRAMINE

- Falcon, S., Ryan, C., Chamberlain, K., & Curtis, G. (1985). Tricyclics: possible treatment for posttraumatic stress disorder. The Journal of clinical psychiatry, 46(9), 385–388.

DESIPRAMINE

- Kauffman, C. D., Reist, C., Djenderedjian, A., Nelson, J. N., Haier, R. J. (1987). Biological markers of affective disorders and posttraumatic stress disorder: a pilot study with desipramine. J Clin Psychiatry, 48(9), 366-367. PMID: 3114242.

NEFAZODONE

- Clark ED, Canive, JM, Calais, LA et al. Nefazodone in post-traumatic stress disorder: a retrospective chart review. Psych-line 1998; 21-28

PHENELZINE

- FJ, Milanes Mack CN, Dennison J, Slater VL: Phenelzine treatment of post-Vietnam syndrome. Practitioner, stress VA June 1984, 40-49 40-49.
